# Plasticity of Airway Epithelial Cell Transcriptome in Response to Flagellin

**DOI:** 10.1371/journal.pone.0115486

**Published:** 2015-02-10

**Authors:** Joan G. Clark, Kyoung-Hee Kim, Ryan S. Basom, Sina A. Gharib

**Affiliations:** 1 Division of Pulmonary and Critical Care Medicine, Department of Medicine, University of Washington, Seattle, Washington, United States of America; 2 Fred Hutchinson Cancer Research Center, Seattle, Washington, United States of America; 3 Computational Medicine Core, Center for Lung Biology, University of Washington, Seattle, Washington, United States of America; University of Pittsburgh, UNITED STATES

## Abstract

Airway epithelial cells (AEC) are critical components of the inflammatory and immune response during exposure to pathogens. AECs in monolayer culture and differentiated epithelial cells in air-liquid interface (ALI) represent two distinct and commonly used *in vitro* models, yet differences in their response to pathogens have not been investigated. In this study, we compared the transcriptional effects of flagellin on AECs in monolayer culture versus ALI culture using whole-genome microarrays and RNA sequencing. We exposed monolayer and ALI AEC cultures to flagellin *in vitro* and analyzed the transcriptional response by microarray and RNA-sequencing. ELISA and RT-PCR were used to validate changes in select candidates. We found that AECs cultured in monolayer and ALI have strikingly different transcriptional states at baseline. When challenged with flagellin, monolayer AEC cultures greatly increased transcription of numerous genes mapping to wounding response, immunity and inflammatory response. In contrast, AECs in ALI culture had an unexpectedly muted response to flagellin, both in number of genes expressed and relative enrichment of inflammatory and immune pathways. We conclude that *in vitro* culturing methods have a dramatic effect on the transcriptional profile of AECs at baseline and after stimulation with flagellin. These differences suggest that epithelial responses to pathogen challenges are distinctly different in culture models of intact and injured epithelium.

## Introduction

The paradigm of the lung epithelium as a physical barrier to injurious substances and infectious agents has evolved with the recognition that airway epithelial cells (AECs) are important modulators of the host’s acute inflammatory and immune response to pathogens [1]. Moreover, AECs are not just targets of lung injury but also contribute to repair processes by proliferating, restoring intact cell barriers and facilitating extracellular matrix remodeling [2,3].

Evidence for the pleotropic role of human epithelial cells in lung defense, immune and inflammatory processes and responses to lung injury has been based, in large part, from *in vitro* studies, including cell lines and primary lung epithelial cell cultures in both submerged monolayer and air liquid interface (ALI) systems [4]. Murine models, especially those involving genetically modified mice, have supported biological inferences derived from studies of AECs in culture [5–8]. ALI-cultured human AEC and large airway AECs freshly obtained from human subjects may share similar transcriptional profiles [9,10], although we recently reported significant differences in microRNA expression between primary cells and ALI cultures [11]. Nevertheless, previous reports using microarray analysis indicated that AEC gene expression profiles representing numerous biological processes undergo extensive changes during differentiation in ALI culture [12,13].

These studies support the fidelity of ALI culture of human AEC as a reasonable *in vitro* model of intact epithelium. The *in vitro* culture process is similar to the morphological changes observed *in vivo* after lung injury [14] in which cells differentiate to basal, goblet and ciliated epithelial cells. The plasticity of epithelial cells raises the question of how AECs cultured under different *in vitro* conditions respond to external challenges such as pathogens.

Flagellin is the primary component of flagella [15] and is recognized by and activates several pathogen recognition receptors, including TLR5 and TLR2 [16–20]. In the lungs of mice, flagellin can induce neutrophils accumulation, an effect that is dependent on TLR5 expression by lung structural cells rather than bone marrow derived cells [21]. Moreover, flagellin stimulates protective immunity to *P. aerugenosa* in a lethal murine model of pneumonia [8]. A direct stimulatory effect of flagellin on lung epithelial cells is supported by *in vitro* expression of AEC proteins such as cytokines and matrix metalloproteinases [3,19,22,23]. Thus, flagellin may induce a broad range of responses in airway epithelial cells and serves as a prototypical external challenge to lung defense and homeostasis.

In this study, we examined the transcriptional state of two widely used human AEC models—submerged monolayer and ALI cultures—with the assumption that these *in vitro* systems represent the spectrum of AEC cell phenotypes present in injured and intact lung. We compared global gene expression between monolayer and ALI cultures at baseline and in response to flagellin. In each condition, the AEC transcriptome was interrogated using whole-genome microarrays and RNA sequencing (RNA-seq).

## Materials and Methods

Detailed Methods are available as supporting information (S1 Text).

### Ethics statement

The Institutional Review Board at Fred Hutchinson Cancer Research Center determined that since the tissue source was anonymous, it was not human research (NHR) and therefore waived the need for ethical review and informed consent. This policy was in accordance with Office for Human Research Protections guidelines (www.hhs.gov/ohrp/policy/cdebiol.html).

### Human tracheal epithelial cell cultures and stimulation with flagellin

Human tracheal tissue was obtained from an anonymized lung transplant donor at University of Washington Medical Center and subjected to enzymatic digestion as described by Fulcher *et al*. [24] to isolate tracheal epithelial cells. Monolayer cells were grown on human placental collagen coated tissue culture plates. Cells were grown until 90% confluent and then subcultured or harvested for cryopreservation. To create ALI cultures, AECs were plated at 2.5 × 10^5^ cells per cm^2^ directly onto collagen-coated semipermeable supports. Approximately 4 weeks after plating, cells became well differentiated, containing both ciliated and mucous producing goblet cells with a transepithelial resistance > 500 Ω•cm^2^.

When monolayer cultures reached ~ 90% confluency or the ALI cultures reached transepithelial electrical resistance > 500 Ω•cm^2^, the AECs were stimulated with 1 μg/ml ultrapure flagellin (endotoxin contamination < 0.05 EU/μg) isolated from *Pseudomonas aeruginosa* (InvivoGen, San Diego, CA) for 4 hours at 37°C. The flagellin concentration of 1 μg/ml was based on previous reports [19,20], and our dose response experiments (0.1, 1, and 10 μg/ml) on ALI and monolayer cultures using an ELISA readout of IL8, a known flageIlin-induced inflammatory cytokine, and CCL20, a differentially up-regulated product identified from the current study (S1 Fig.). Conditioned media were collected for ELISA, and the epithelial cells were lysed in TRIzol and frozen at −80°C for RNA isolation. For all ELISA and RNA analyses, conditioned media and RNA were derived from one donor. The same RNA samples were used for PCR, microarrays and RNA-seq. All RNA-seq experiments were performed in duplicate, whereas the microarray experiments were either in triplicate (ALI cultures with and without flagellin exposure) or duplicate (monolayer cultures with and without flagellin exposure).

### RNA isolation, qPCR and ELISA

Total RNA was isolated by TRIzol reagent (Invitrogen) and assessed for quality by Bioanalyzer (Agilent Technologies, Fort Worth, TX). Real time PCR was performed using TaqMan primer probe sets in an ABI 7900 HT PCR system. The 18S component of rRNA was used as the endogenous control and expression levels of the tested genes were normalized to its expression using the delta-delta approach [25]. Protein levels of CCL20 and IL8 in monolayer and ALI culture medium at baseline and following stimulation with flagellin were measured using ELISA kits (R&D System, Minneapolis, MN). The *P*-values for CCL20 and IL8 protein expression differences across samples was calculated using one-way ANOVA (GraphPad Software, La Jolla, CA).

### Microarray

Total RNA (100 ng) from stimulated (flagellin 1 μg/ml) and unstimulated tracheal epithelial cells in either monolayer or ALI cultures were labeled hybridized to Human Gene ST 1.0 microarrays (Affymetrix) following the manufacturer’s protocols. Image acquisition was performed using the GeneChip Operating System (GCOS). Probe intensities were normalized using the RMA procedure. Differential gene expression was determined using a Bayesian implementation of the parametric *t*-test [26]. Correction for multiple testing was performed using Benjamini-Hochberg’s method [27], with an adjusted *P*-value ≤ 0.01 designated as significant.

### RNA-seq

Total RNA (1 μg) from stimulated (flagellin 1 μg/ml) and unstimulated tracheal epithelial cells in either monolayer or ALI cultures was used for library generation using Illumina’s TruSeq RNA protocol (Illumina, San Diego, CA). Image analysis and base calling were performed with Illumina’s RTA (v1.12) software, followed by demultiplexing and FASTQ format file generation with Illumina’s CASAVA (v1.8) and alignment to UCSC’s hg19 genome build using TopHat (v1.3.1) [28]. The average number of alignments per run was approximately 20.5 million. Gene counts were generated using htseq-count (v0.5.1, http://www-huber.embl.de/users/anders/HTSeq/doc/count.html). For gene expression analysis, we initially removed all genes that had less than 1 count/million in at least half the samples. Differential gene expression analysis was performed using edgeR [29]. Correction for multiple testing was implemented using Benjamini-Hochberg’s method [27], with an adjusted *P*-value ≤ 0.01 designated as significant.

### Correspondence and enrichment analyses

Multidimensional scaling using correspondence analysis was applied to compare variability in global gene expression across all samples as assessed by microarrays and RNA-seq [30,31]. This analysis was based on unique genes identified commonly between the microarray platform and RNA-seq.

Functional enrichment analysis of differentially expressed AEC genes in (i) unstimulated monolayer vs. unstimulated ALI, (ii) flagellin-exposed monolayer cultures, and (iii) flagellin-exposed ALI cultures, was performed using the DAVID online software tool and based on Gene Ontology annotations [32]. Since the number of differentially expressed genes in condition (i) was very large, we limited the enrichment analysis to the top 1000 most significant genes. Enrichment *P*-values were corrected for multiple testing using Benjamini-Hochberg’s method [27].

### Data availability

Detailed microarray and RNA-seq information including access to all raw data, meeting Minimum Information About a Microarray Experiment (MIAME) requirements, has been deposited at Gene Expression Omnibus (http://www.ncbi.nlm.nih.gov/geo, GSE55460).

## Results

### Variability in AEC transcriptome is strongly influenced by culturing method

**Fig 1:**
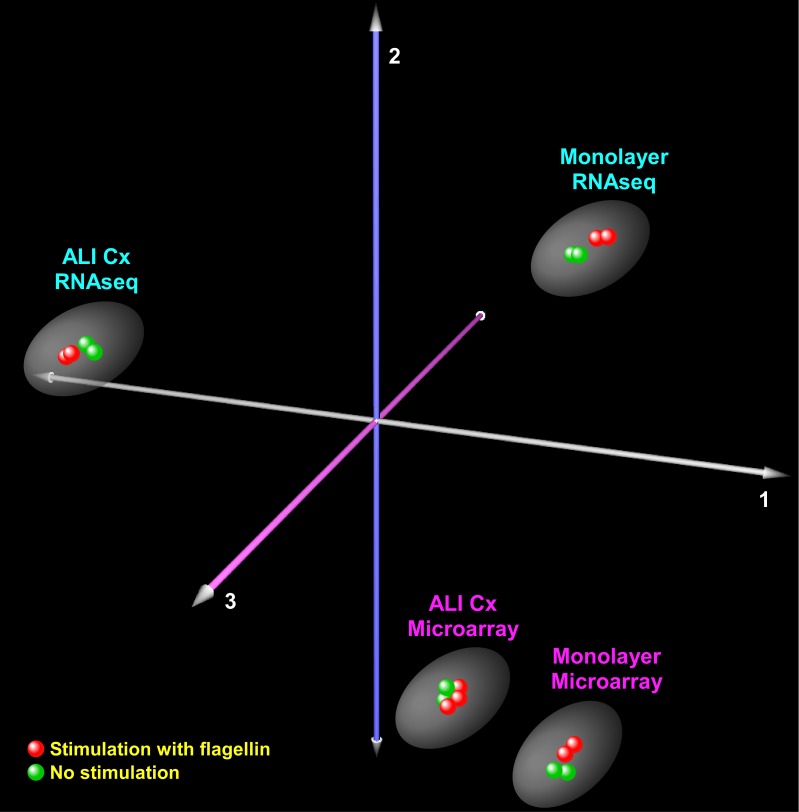
Correspondence analysis of AEC transcriptome. In this analysis, the contributions of: (i) culture methodology (monolayer, ALI); (ii) whole-genome transcription profiling platform (RNA-seq, microarray); and (iii) stimulation with flagellin on gene expression variability are shown. The three depicted axes capture more than 95% of the total transcriptional variability and the distance among samples reflects differences in gene expression. Note the large distance between monolayer vs. ALI cultured AECs as assessed by RNA-seq, implying that these samples account for a significant proportion of total gene expression variability. In contrast, exposure to flagellin (as assessed by RNA-seq or microarray) has a much more modest contribution.

We applied correspondence analysis to segregate the AEC samples based on culture system (i.e., monolayer vs. ALI), gene expression interrogation method (i.e., microarray vs. RNA-seq), and exposure (i.e., unstimulated vs. flagellin). This analysis was based on 15,238 unique genes common to both platforms (Fig. 1). The first three orthogonal components shown in Fig. 1 captured over 95% of the observed gene expression variability, with axis 1 alone explaining 52% of the transcriptional differences. The most significant determinant of sample segregation was the culturing method used. However, RNA-seq captured substantially more transcriptional variability associated with monolayer vs. ALI culture systems compared to microarray, as demonstrated by the wider distance between samples (Fig. 1). In this context, the transcriptional effects of flagellin stimulation were mild compared to the differences due to culture methodology for both the microarray experiments and RNA-seq.

### Unstimulated monolayer and ALI AEC cultures are characterized by profound differences in gene expression

**Fig 2:**
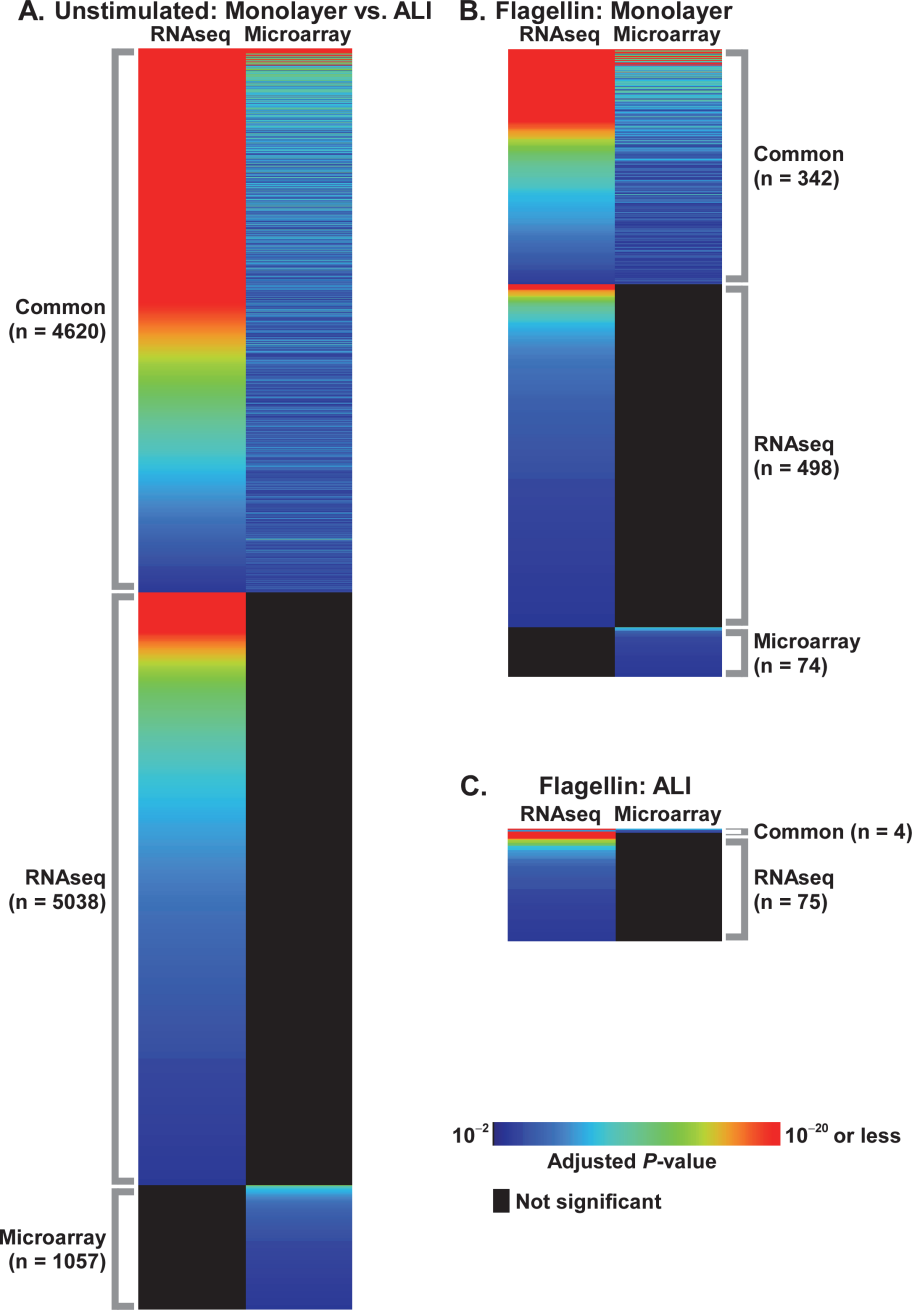
Heatmap depiction of differentially expressed genes as determined via RNA-seq and microarrays under multiple experimental conditions: (A) unstimulated AECs cultured in monolayer vs. ALI; (B) unstimulated vs. flagellin-exposed AECs cultured in monolayer; (C) unstimulated vs. flagellin-exposed AECs cultured in ALI. Note the very large number of differentially expressed genes between ALI vs. monolayer AEC cultures at baseline. When stimulated with flagellin, AECs grown in ALI displayed a blunted transcriptional response when compared to the robust response of epithelial cells cultured in monolayer. In each condition, RNA-seq identified many more differentially expressed genes compared to microarrays while capturing the majority of genes deemed significant by microarrays. Heatmaps are based on adjusted *P*-values of differentially expressed genes, and for purposes of clarity, are not drawn to the same scale in height across conditions.

**Fig 3:**
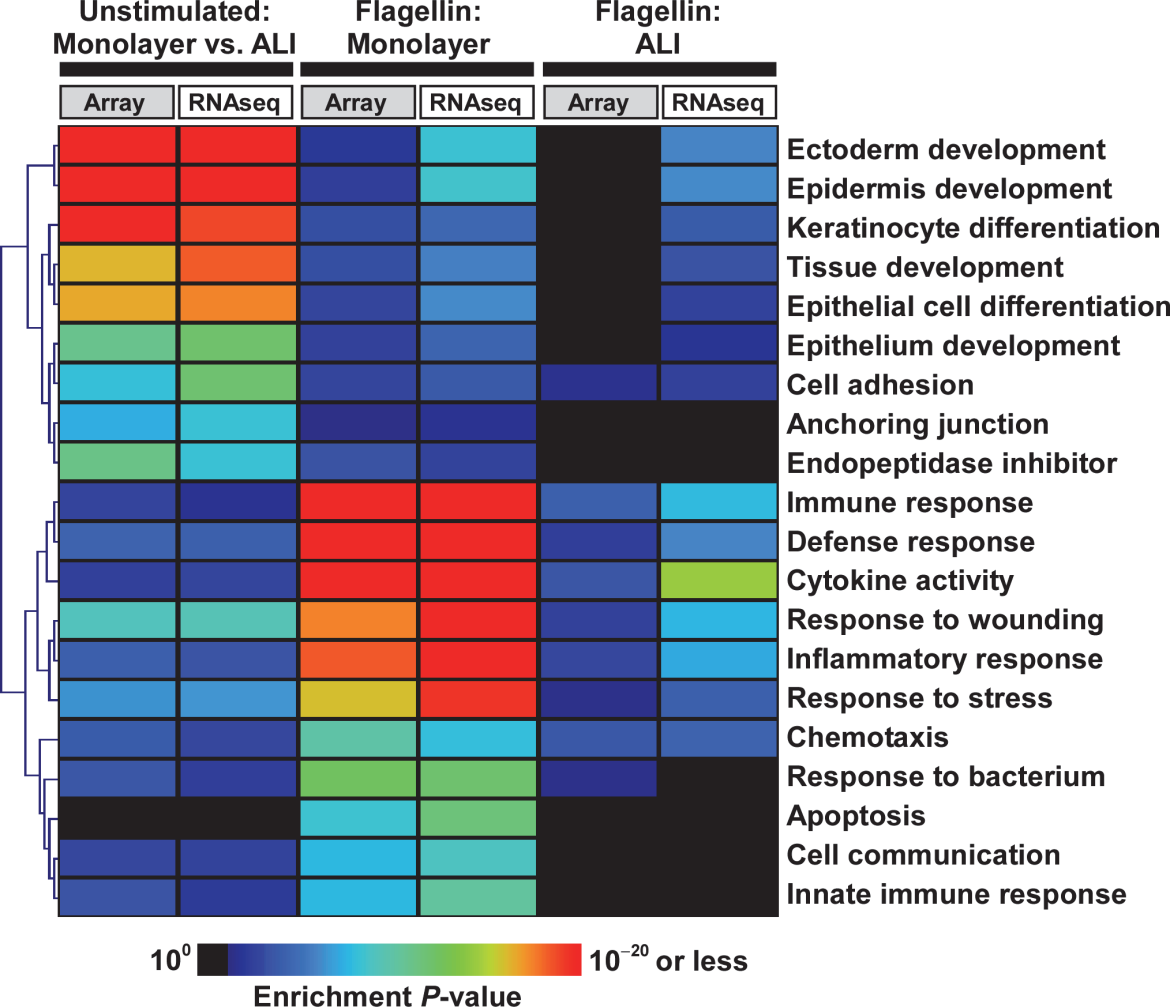
Heatmap representation of select, highly enriched GO categories among differentially expressed genes under multiple conditions: (i) unstimulated AECs cultured in monolayer vs. ALI; (ii) unstimulated vs. flagellin-exposed AECs cultured in monolayer; and (iii) unstimulated vs. flagellin-exposed AECs cultured in ALI. Note the enrichment of developmental programs among differentially expressed genes in monolayer vs. ALI cultured AECs. When stimulated with flagellin, AECs grown in monolayer are characterized by activation of pro-inflammatory and immune responses, whereas ALI-cultured AECs display a muted transcriptional response. Despite differences in the number of differentially expressed genes identified by RNA-seq vs. microarrays, similar patterns of functional enrichment were seen between the two platforms. Complete lists of enriched GO categories are provided in S1–S6 Tables.

Since correspondence analysis attributed significant transcriptional effects to the culturing methodology, we proceeded to identify differentially expressed genes between unstimulated monolayer and ALI samples. Using strict statistical criteria, we found dramatic differences in gene expression between AECs grown in monolayer versus those cultured in ALI (Fig. 2A). Although this effect was demonstrated using both microarrays and RNA-seq, whole-transcriptome sequencing identified many more differentially expressed genes (9,658 vs. 5,677). Despite differences in the number of genes identified between microarrays and RNA-seq, functional enrichment analysis of these differentially expressed genes revealed that similar overrepresented biological categories were captured by both approaches. Prominently, these shared processes mapped to developmental and remodeling programs such as ectoderm/epidermis development, epithelial cell differentiation/development, cell adhesion, and response to wounding (Fig. 3, S1, S2 Tables).

### Exposure to flagellin induces a significant transcriptional response in monolayer AECs and a blunted response in ALI AECs

**Fig 4:**
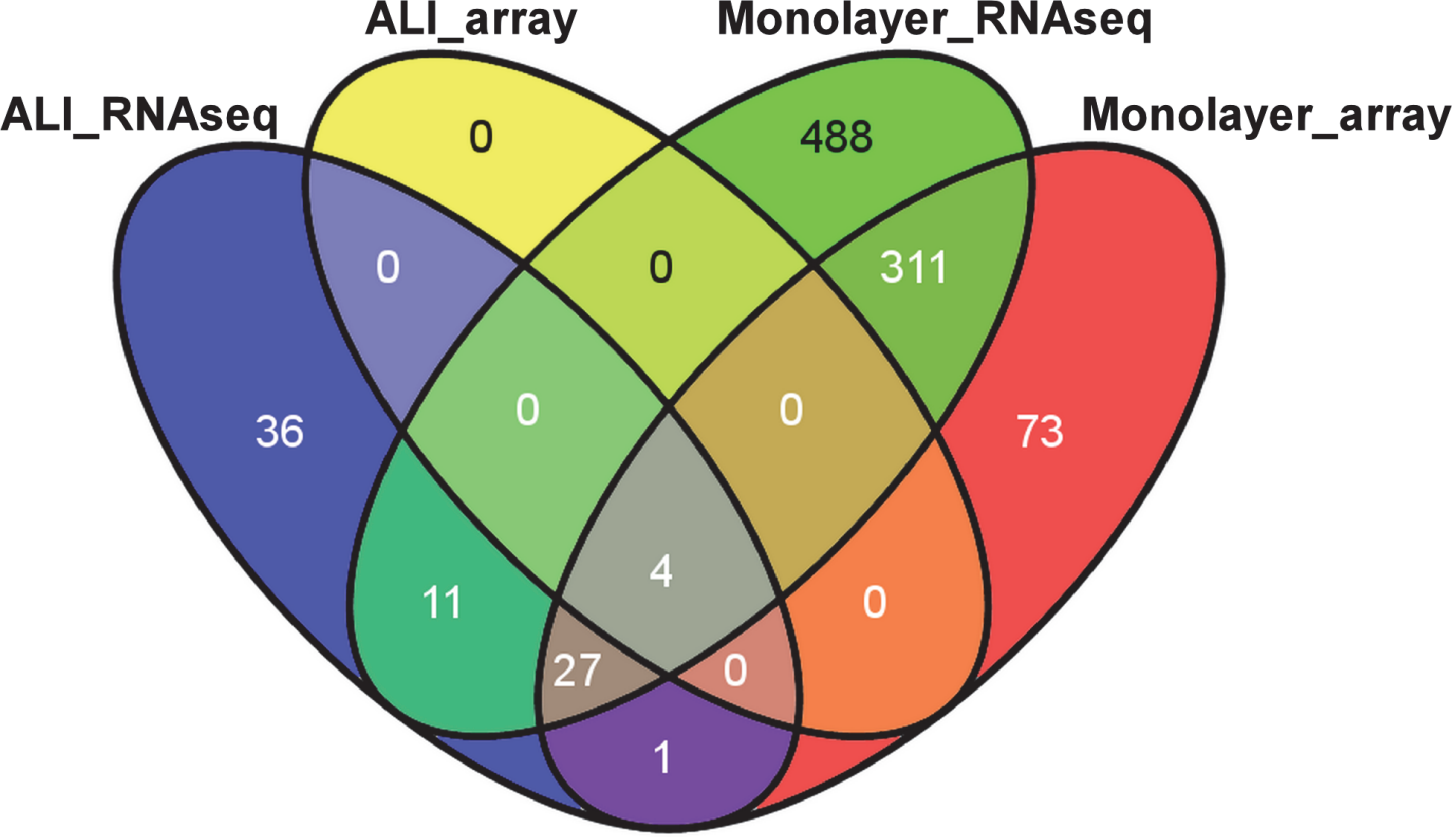
A Venn diagram summarizing overlaps among differentially expressed genes across multiple conditions and measurements: (i) flagellin-induced genes in AECs cultured in ALI as measured by RNA-seq (ALI_RNAseq); (ii) flagellin-induced genes in AECs cultured in ALI measured by microarrays (ALI_array); (iii) flagellin-induced genes in AECs cultured in monolayer as measured by RNA-seq (Monolayer_RNAseq); (iv) flagellin-induced genes in AECs cultured in monolayer as measured by microarrays (Monolayer_array). Note the widespread transcriptional response of AECs to flagellin in monolayer cultures compared to cells grown in ALI. For a given exposure condition, RNA-seq identified a much larger set of differentially expressed genes compared to microarrays. Venn diagram was generated using online tool Venny (http://bioinfogp.cnb.csic.es/tools/venny).

AECs cultured in monolayer demonstrated widespread differential gene expression when stimulated with flagellin. RNA-seq identified 841 and microarrays identified 416 unique differentially expressed genes in response to flagellin (Fig. 2B). In sharp contrast, when ALI cultures were exposed to the same dose of flagellin, the expression level of a very limited set of genes was changed (79 by RNA-seq, 4 by microarray, Fig. 2C). Overlapping genes in the various conditions are summarized in Fig. 4. Note that only 4 genes were differentially expressed in response to flagellin across all conditions and measurements (CCL20, ICAM1, CCL5, and IL8).

In monolayer cultures, flagellin activated highly enriched processes involved in inflammation, immunity, chemotaxis and apoptosis, whereas ALI cultures demonstrated a much more restricted repertoire of modestly enriched functional categories (Fig. 3, S3–S6 Tables). The widely variable range of differentially expressed genes and their corresponding pathway enrichment implied that AECs display profoundly different responses to a prototypical pathogenic challenge depending on whether they were cultured as monolayers or allowed to differentiate under an air-liquid interface system.

### The fidelity of RNA-seq in assessing AEC gene expression is superior to highly sensitive whole-genome microarrays

**Fig 5:**
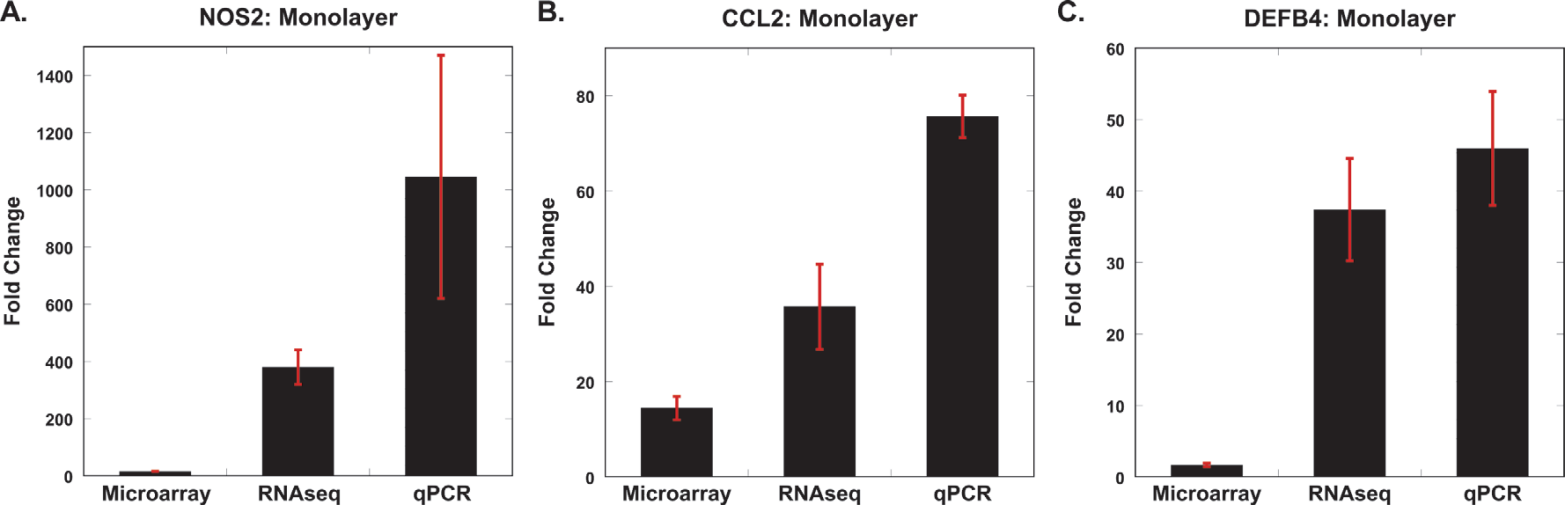
Comparison between microarray, RNA-seq, and qPCR for several statistically significantly differentially expressed candidate genes. Three highly up-regulated genes in response to flagellin in monolayer-cultured AECs (NOS2, CCL2, DEFB4) were evaluated by qPCR. While the “gold standard” method for quantitative gene expression analysis is debatable, RNA-seq and qPCR results were more closely matched (see also Fig. 6). For each condition, mean value ± standard deviation is shown.

**Fig 6:**
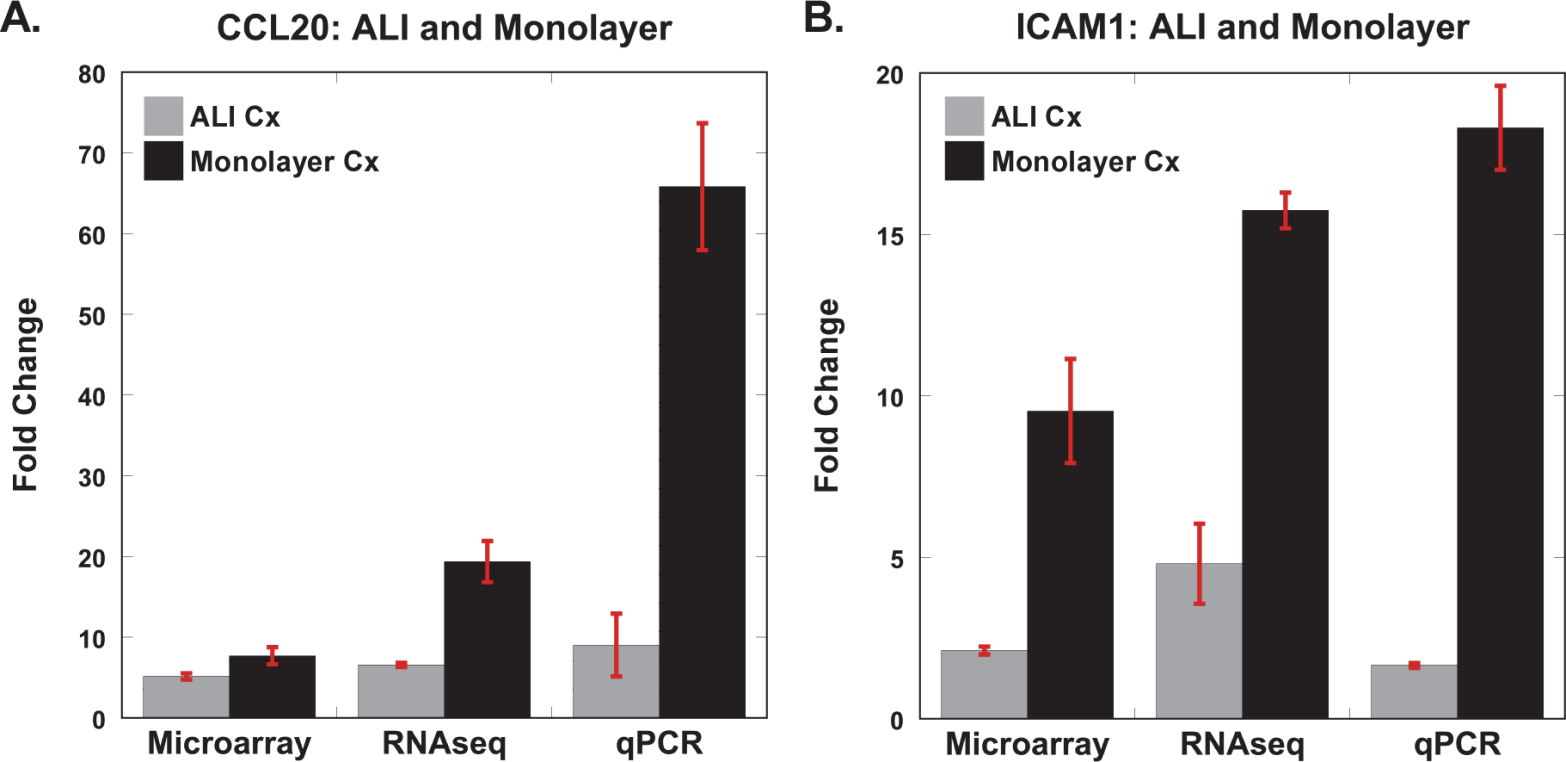
Comparison of flagellin-induced differential gene expression in ALI cultures of AECs. Although only a few genes were statistically significantly differentially expressed in ALI-cultured AECs in response to flagellin, we evaluated two top candidates (CCL20, ICAM1) using qPCR. However, note that even these two candidate genes were more significantly up-regulated following flagellin exposure in the monolayer culture system compared to ALI culture. For each condition, mean value ± standard deviation is shown.

**Fig 7:**
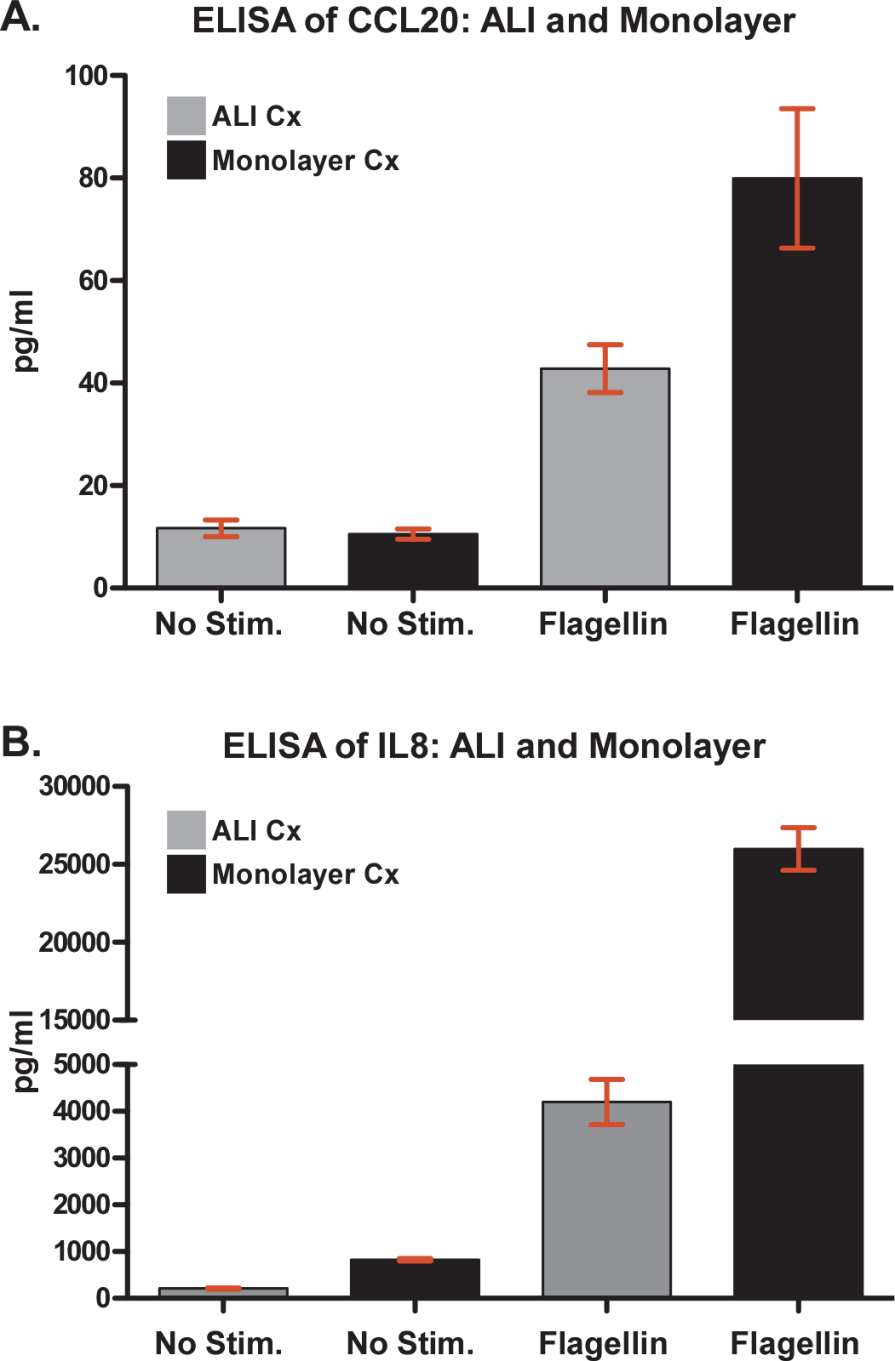
Validation of differential protein expression for up-regulated candidate genes. We used ELISA to assess secreted protein levels of (A) CCL20 and (B) IL8 at baseline and following stimulation with flagellin. CCL20 and IL8 was selected because they were transcriptionally up-regulated in both ALI and monolayer AEC cultures in response to flagellin. For each condition, mean value ± standard deviation is shown. One-way ANOVA *P*-values across conditions: *P* < 0.005 for CCL20 and *P* < 0.0001 for IL8.

Our study design, whereby AEC samples from the same subject were interrogated via microarrays and RNA-seq, provided us the opportunity to compare the performance of the two platforms. We identified 16,369 unique genes across all samples using RNA-seq, with gene counts ranging from 1 count/million to 520,967 counts/million—a dynamic range > 5×10^5^. In comparison, the dynamic range of microarrays (based on normalized transcript intensity across samples) was less than 10^4^. Consistent with these observations, the maximum fold-change in gene expression between unstimulated and flagellin-exposed samples was ~400-fold (for NOS2 in monolayer) using RNA-seq, but even though NOS2 was also identified as the most up-regulated gene in the microarray experiments, it was substantially less changed (~15-fold). To confirm these findings, we compared differential expression of multiple candidates using qPCR (Fig. 5). We observed that NOS2 expression in monolayer cultures was up-regulated ~1000-fold in response to flagellin based on qPCR, more closely matching the RNA-seq results. Similar expression patterns were seen for the other candidates (e.g., CCL2, DEFB4), with RNA-seq and qPCR reporting the largest fold-changes (Fig. 5). Although only a limited number of genes were differentially expressed in ALI cultures exposed to flagellin, we validated two of the most significantly up-regulated genes in the ALI system—CCL20 and ICAM1—using qPCR (Fig. 6). We observed that even for these genes, exposure to flagellin induced a higher transcriptional response in monolayer AECs compared to ALI cultures, and that RNA-seq was more sensitive than microarrays in capturing the fidelity of gene expression. This response pattern was corroborated at the protein level for CCL20 and IL8 (Fig. 7).

## Discussion

Airway epithelial cells play a central role in airway homeostasis and response to lung injury [33]. Here we show markedly different transcriptional states between two commonly used *in vitro* models of AECs: monolayer and ALI cultures. We also report widespread differences between the transcriptional responses of these culture systems to flagellin, suggesting that they model fundamentally different aspects of lung epithelial cell biology.

The dramatically altered AEC transcriptome between unstimulated monolayer and ALI cultures was highly enriched in processes mapping to various developmental programs. This observation makes sense given the profound morphological differences observed between confluent monolayer epithelial cells and those differentiating under an air-liquid interface. Our results are also consistent with previous studies reporting large transcriptional differences between monolayers of airway-derived cells and differentiated airway epithelial cells in ALI culture based on microarray analysis [10], however, our study further extends this observation using RNA-seq. Accordingly, AECs in monolayer cultures may recapitulate an epithelium undergoing repair, whereas AECs in mature ALI cultures represent an intact airway epithelium [13]. Collectively, these findings highlight the importance of recognizing the significant consequences of culture methodology on baseline epithelial gene expression.

The strikingly different response between the two culture systems when exposed to flagellin has important implications for *in vitro* modeling of airway injury and repair. Notably, AECs in monolayer responded to this bacterial product with increases in a wide range of gene transcripts involved in immunity, wound repair, and inflammation. In contrast, cells in ALI culture had a decidedly muted response both in number of genes with increased transcriptional activity, the magnitude of these changes, and the relative enrichment of functional pathways. Thus, in the presence of an intact epithelial barrier (as modeled by ALI), exposure to a bacterial product results in a tempered response from AECs. Such a measured effect may serve to regulate the activation of excessive pro-inflammatory programs. However, AEC response to bacterial products in the setting of lung injury or disease states might not be restrained to the same degree. This primed state of the lung epithelium may contribute to the pathogenesis of pulmonary disorders characterized by epithelial injury such as ventilator-associated pneumonia, acute lung injury, post-viral bacterial pneumonia, and cystic fibrosis.

The AEC culture models used in this study are operationally defined according to widely used methods reported in the literature. Although variations of culture methods might influence gene expression, the profound differences that we report are likely to be generalizable. However, it is important to note that *in vitro* culture systems do not fully capture the *in vivo* state of AECs at baseline or following injury. We recently reported that microRNA profiles are significantly different between AECs cultured in ALI and primary epithelial cells harvested *in vivo* [11].

In this study, we had the opportunity to compare the robustness of whole-genome microarrays and RNA-seq in interrogating the transcriptional state of AECs under tightly controlled conditions. We found that consistent with previous reports [34,35], RNA-seq possessed higher sensitivity and fidelity in measuring changes in gene expression, but we also observed that there was substantial overlap between both approaches in identifying similar enriched biological processes populated by differentially expressed genes.

While our transcriptional analyses provide a relatively comprehensive overview of epithelial cell biology, there are important limitations in our approach. All the cultured AEC samples were derived from a single donor, therefore we didn’t capture inter-individual variability in gene expression. Other investigators have used this approach to study the transcriptional profile of AECs [36]. Furthermore, this design is similar to the common usage of human cell culture lines that have been originally harvested from single individuals. In our case, this experimental design allowed us to directly compare the technical performance of microarray and RNA-seq-based profiling of the AEC transcriptome. The number experimental replicates (n = 2–3) was low, but we applied statistical methods specifically designed for limited replicates, and observed very significant changes in gene expression despite this limitation. It is well recognized that transcriptional activity does not equate biological effects due to post-transcriptional modifications of gene products. We only investigated the transcriptional consequences of flagellin on monolayer and ALI cultures; other pathogen-associated molecular pattern molecules may elicit different responses. Although we observed significant differences in transcriptional activity between monolayer and ALI AECs after 4 hours of exposure to flagellin, we did not assess changes in gene expression at additional time points between the two culture methods. While hypothesis generating, our analyses do not delineate mechanisms regulating the observed transcriptional differences. Future studies are needed to define these mechanisms that will likely involve multiple molecular pathways. However, our microarray and RNA-seq data have been made publicly available as a resource for further analysis of epithelial cell biology and its response to injury and pathogens.

## Conclusions

Collectively, our findings imply that different *in vitro* culture systems modeling *in vivo* AEC biology are characterized by significant differences in baseline gene expression and transcriptional responses to flagellin. While such systems are useful research tools and capture certain features of intact and injured airway epithelium, results obtained from *in vitro* models should be interpreted with an appreciation of their distinctly altered expression profiles.

## Supporting Information

S1 Fig Dose response characteristic of monolayer and ALI cultures of AECs to flagellin. (PDF)

S1 Table Functional enrichment of differentially expressed genes between monolayer vs. ALI AEC cultures as identified by exon microarrays. (PDF)

S2 Table Functional enrichment of differentially expressed genes between monolayer vs. ALI AEC cultures as identified by RNA-seq. (PDF)

S3 Table Functional enrichment of differentially expressed genes following exposure to flagellin in monolayer AEC cultures as identified by exon microarrays. (PDF)

S4 Table Functional enrichment of differentially expressed genes following exposure to flagellin in monolayer AEC cultures as identified by RNA-seq. (PDF)

S5 Table Functional enrichment of differentially expressed genes following exposure to flagellin in ALI AEC cultures as identified by exon microarrays. (PDF)

S6 Table Functional enrichment of differentially expressed genes following exposure to flagellin in ALI AEC cultures as identified by RNA-seq. (PDF)

S1 Text Detailed Materials and Methods. (PDF)
