# Supplementary material for: Plasticity of Airway Epithelial Cell Transcriptome in Response to Flagellin
Source: PLoS One. 2015 Feb 10;10(2):e0115486. doi: 10.1371/journal.pone.0115486 (PMC4323341; doi:10.1371/journal.pone.0115486)

Supplementary Figure 1. Dose response characteristic of monolayer and ALI cultures of airway epithelial cells to flagellin. Protein levels of IL8 (panels A, B) and CCL20 (panels C, D) were measured in culture medium after 4 hours of stimulation with flagellin at 0.1, 1, and 10 µg/ml using ELISA. A flagellin concentration of 1 µg/ml was chosen for subsequent experiments given the robust response at this dose. ELISAs were performed in duplicate and for each condition mean value ± standard deviation is shown.

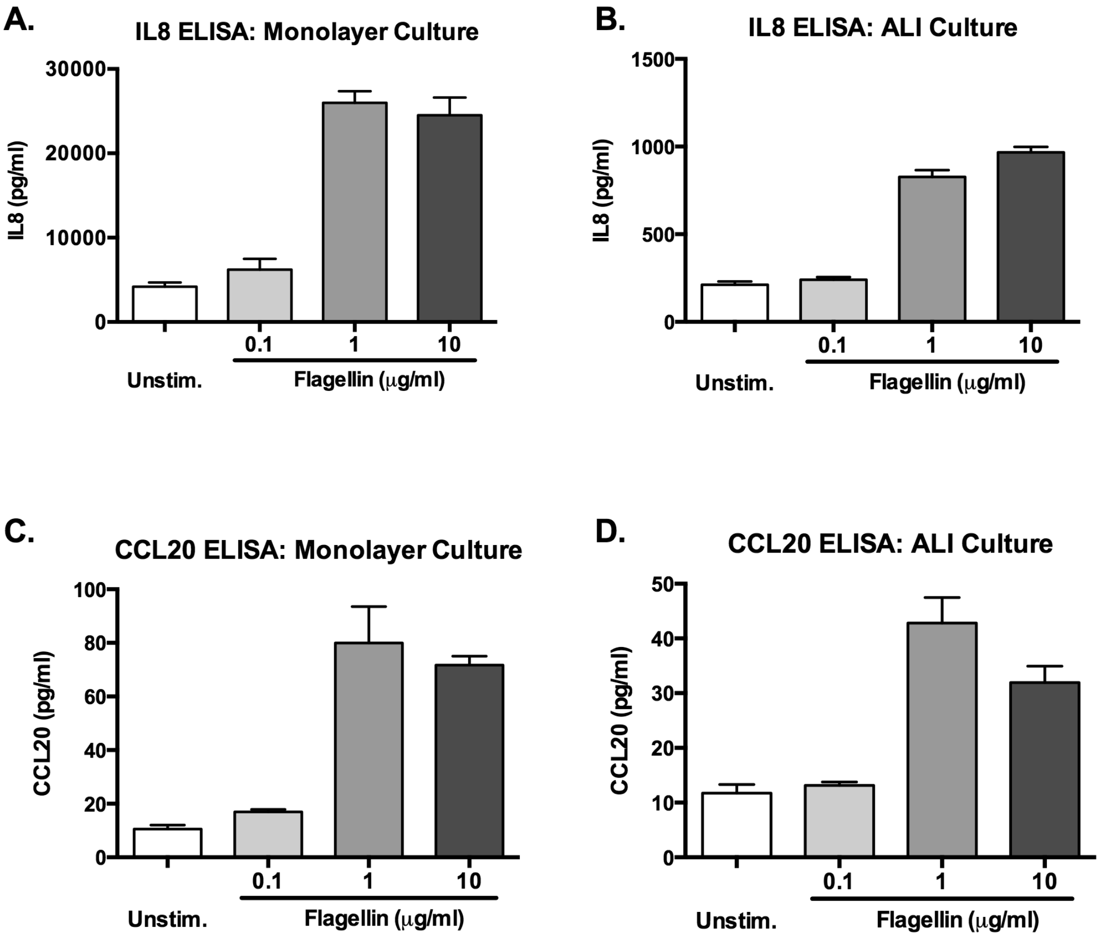

Supplement: S1 Fig — (PDF) [file pone.0115486.s001.pdf]
