# Supplementary material for: Plasticity of Airway Epithelial Cell Transcriptome in Response to Flagellin
Source: PLoS One. 2015 Feb 10;10(2):e0115486. doi: 10.1371/journal.pone.0115486 (PMC4323341; doi:10.1371/journal.pone.0115486)
Supplement: S1 Table — (PDF) [file pone.0115486.s002.pdf]

Supplementary Table 1. Complete results from functional enrichment analysis of differentially expressed genes between monolayer vs. ALI AEC cultures as identified by exon microarrays.

Gene Ontology Annotation	Fold Enrichment	P-value	Adjusted P-value
GO:0007398~ectoderm development	5.52	1.51E-28	4.99E-25
GO:0008544~epidermis development	5.68	7.52E-28	1.24E-24
GO:0009913~epidermal cell differentiation	8.00	1.54E-20	1.70E-17
GO:0030216~keratinocyte differentiation	8.18	1.34E-19	1.11E-16
GO:0031424~keratinization	9.63	5.79E-17	7.35E-14
GO:0030855~epithelial cell differentiation	4.99	2.47E-16	1.22E-13
GO:0009888~tissue development	2.49	5.58E-16	2.62E-13
GO:0005576~extracellular region	1.74	6.38E-15	2.79E-12
GO:0001533~cornified envelope	12.60	3.50E-13	7.73E-11
GO:0048513~organ development	1.70	3.03E-12	1.25E-09
GO:0004866~endopeptidase inhibitor activity	4.05	3.75E-11	3.83E-08
GO:0060429~epithelium development	3.25	6.05E-11	2.22E-08
GO:0004867~serine-type endopeptidase inhibitor activity	4.99	7.72E-11	3.95E-08
GO:0030414~peptidase inhibitor activity	3.84	1.59E-10	5.40E-08
GO:0048731~system development	1.52	2.44E-10	8.06E-08
GO:0042221~response to chemical stimulus	1.76	3.05E-10	9.16E-08
GO:0048856~anatomical structure development	1.48	6.56E-10	1.81E-07
GO:0009605~response to external stimulus	1.89	1.38E-09	3.50E-07
GO:0044421~extracellular region part	1.87	2.56E-09	3.76E-07
GO:0009611~response to wounding	2.21	2.91E-09	6.87E-07
GO:0006629~lipid metabolic process	1.93	3.89E-09	8.59E-07
GO:0005737~cytoplasm	1.21	6.92E-09	7.63E-07
GO:0007155~cell adhesion	1.95	2.45E-08	5.06E-06
GO:0022610~biological adhesion	1.95	2.59E-08	5.04E-06
GO:0007275~multicellular organismal development	1.39	4.76E-08	8.74E-06
GO:0032502~developmental process	1.36	6.10E-08	1.06E-05
GO:0070161~anchoring junction	3.12	1.57E-07	1.39E-05
GO:0004857~enzyme inhibitor activity	2.58	1.99E-07	5.09E-05
GO:0005509~calcium ion binding	1.74	4.62E-07	9.44E-05
GO:0016323~basolateral plasma membrane	2.82	5.02E-07	3.69E-05
GO:0005615~extracellular space	1.86	8.21E-07	5.17E-05
GO:0006950~response to stress	1.49	1.08E-06	1.79E-04
GO:0042127~regulation of cell proliferation	1.76	1.30E-06	2.05E-04
GO:0044459~plasma membrane part	1.42	1.30E-06	7.18E-05
GO:0031012~extracellular matrix	2.25	1.77E-06	8.68E-05
GO:0005925~focal adhesion	3.62	1.96E-06	8.63E-05
GO:0070482~response to oxygen levels	3.06	2.90E-06	4.36E-04
GO:0005912~adherens junction	2.98	2.98E-06	1.19E-04
GO:0040011~locomotion	2.05	3.06E-06	4.40E-04
GO:0005924~cell-substrate adherens junction	3.49	3.56E-06	1.31E-04
GO:0005783~endoplasmic reticulum	1.66	3.99E-06	1.35E-04
GO:0044255~cellular lipid metabolic process	1.92	4.09E-06	5.63E-04
GO:0001666~response to hypoxia	3.09	4.26E-06	5.63E-04
GO:0005578~proteinaceous extracellular matrix	2.25	4.29E-06	1.35E-04
GO:0010033~response to organic substance	1.75	5.81E-06	7.39E-04
GO:0018149~peptide cross-linking	6.93	6.31E-06	7.73E-04
GO:0031100~organ regeneration	6.93	6.31E-06	7.73E-04
GO:0005886~plasma membrane	1.27	6.34E-06	1.86E-04
GO:0042060~wound healing	2.64	6.89E-06	8.14E-04
GO:0030055~cell-substrate junction	3.30	8.23E-06	2.27E-04

GO:0006928~cell motion	1.93	9.42E-06	1.07E-03
GO:0008610~lipid biosynthetic process	2.17	9.50E-06	1.05E-03
GO:0030154~cell differentiation	1.44	1.12E-05	1.19E-03
GO:0048519~negative regulation of biological process	1.41	1.28E-05	1.33E-03
GO:0016020~membrane	1.15	1.87E-05	4.86E-04
GO:0030155~regulation of cell adhesion	2.89	2.07E-05	2.08E-03
GO:0031099~regeneration	3.91	2.08E-05	2.02E-03
GO:0042598~vesicular fraction	2.35	2.21E-05	5.40E-04
GO:0051270~regulation of cell motion	2.52	2.41E-05	2.28E-03
GO:0005856~cytoskeleton	1.47	3.21E-05	7.44E-04
GO:0005792~microsome	2.34	3.32E-05	7.33E-04
GO:0051259~protein oligomerization	2.59	3.33E-05	3.05E-03
GO:0009725~response to hormone stimulus	2.01	3.34E-05	2.99E-03
GO:0048869~cellular developmental process	1.40	3.43E-05	2.98E-03
GO:0016705~oxidoreductase activity, acting on paired donors, with incorporatic	2.86	4.11E-05	6.98E-03
GO:0003824~catalytic activity	1.19	4.20E-05	6.12E-03
GO:0016477~cell migration	2.15	6.27E-05	5.30E-03
GO:0032787~monocarboxylic acid metabolic process	2.09	6.31E-05	5.20E-03
GO:0006952~defense response	1.70	9.13E-05	7.34E-03
GO:0045785~positive regulation of cell adhesion	3.90	9.29E-05	7.29E-03
GO:0006631~fatty acid metabolic process	2.36	1.01E-04	7.74E-03
GO:0006979~response to oxidative stress	2.53	1.06E-04	7.97E-03
GO:0008285~negative regulation of cell proliferation	1.95	1.10E-04	8.05E-03
GO:0065008~regulation of biological quality	1.41	1.13E-04	8.10E-03
GO:0016717~oxidoreductase activity, acting on paired donors, with oxidation of	15.30	1.20E-04	1.52E-02
GO:0048523~negative regulation of cellular process	1.38	1.23E-04	8.65E-03
GO:0006954~inflammatory response	1.99	1.30E-04	8.92E-03
GO:0032879~regulation of localization	1.68	1.33E-04	8.95E-03
GO:0005102~receptor binding	1.55	1.37E-04	1.54E-02
GO:0030036~actin cytoskeleton organization	2.23	1.39E-04	9.19E-03
GO:0010647~positive regulation of cell communication	1.97	1.65E-04	1.07E-02
GO:0031091~platelet alpha granule	3.96	1.72E-04	3.60E-03
GO:0042330~taxis	2.48	2.06E-04	1.30E-02
GO:0006935~chemotaxis	2.48	2.06E-04	1.30E-02
GO:0048870~cell motility	1.99	2.08E-04	1.29E-02
GO:0051674~localization of cell	1.99	2.08E-04	1.29E-02
GO:0016746~transferase activity, transferring acyl groups	2.26	2.08E-04	2.11E-02
GO:0005515~protein binding	1.11	2.16E-04	1.98E-02
GO:0016042~lipid catabolic process	2.39	2.33E-04	1.42E-02
GO:0033273~response to vitamin	3.55	2.41E-04	1.44E-02
GO:0031982~vesicle	1.63	2.55E-04	5.10E-03
GO:0005887~integral to plasma membrane	1.45	2.57E-04	4.91E-03
GO:0004252~serine-type endopeptidase activity	2.50	2.62E-04	2.20E-02
GO:0043436~oxoacid metabolic process	1.68	2.75E-04	1.61E-02
GO:0019752~carboxylic acid metabolic process	1.68	2.75E-04	1.61E-02
GO:0031093~platelet alpha granule lumen	4.51	2.77E-04	5.08E-03
GO:0009719~response to endogenous stimulus	1.82	2.81E-04	1.62E-02
GO:0017171~serine hydrolase activity	2.35	3.17E-04	2.46E-02
GO:0006082~organic acid metabolic process	1.67	3.25E-04	1.84E-02
GO:0030234~enzyme regulator activity	1.53	3.76E-04	2.71E-02
GO:0030029~actin filament-based process	2.09	3.98E-04	2.21E-02
GO:0040012~regulation of locomotion	2.25	4.08E-04	2.23E-02
GO:0030334~regulation of cell migration	2.34	4.39E-04	2.36E-02
GO:0042180~cellular ketone metabolic process	1.65	4.40E-04	2.32E-02

GO:0009617~response to bacterium	2.24	4.40E-04	2.28E-02
GO:0055114~oxidation reduction	1.61	4.42E-04	2.26E-02
GO:0001558~regulation of cell growth	2.23	4.74E-04	2.38E-02
GO:0031410~cytoplasmic vesicle	1.61	4.74E-04	8.33E-03
GO:0060205~cytoplasmic membrane-bounded vesicle lumen	4.20	4.85E-04	8.19E-03
GO:0019838~growth factor binding	2.80	5.44E-04	3.64E-02
GO:0031226~intrinsic to plasma membrane	1.41	5.65E-04	9.18E-03
GO:0008415~acyltransferase activity	2.19	6.04E-04	3.79E-02
GO:0005200~structural constituent of cytoskeleton	3.22	6.05E-04	3.57E-02
GO:0045087~innate immune response	2.48	6.17E-04	3.05E-02
GO:0043627~response to estrogen stimulus	2.74	6.61E-04	3.21E-02
GO:0031983~vesicle lumen	4.02	6.84E-04	1.07E-02
GO:0008236~serine-type peptidase activity	2.27	6.88E-04	3.83E-02
GO:0016747~transferase activity, transferring acyl groups other than amino-acyl	2.17	6.94E-04	3.66E-02
GO:0016023~cytoplasmic membrane-bounded vesicle	1.65	7.14E-04	1.08E-02
GO:0044425~membrane part	1.13	7.20E-04	1.05E-02
GO:0008305~integrin complex	5.10	7.21E-04	1.02E-02
GO:0032501~multicellular organismal process	1.17	7.41E-04	3.54E-02
GO:0043122~regulation of I-kappaB kinase/NF-kappaB cascade	2.69	8.08E-04	3.80E-02
GO:0006665~sphingolipid metabolic process	3.12	8.09E-04	3.75E-02
GO:0005626~insoluble fraction	1.50	8.26E-04	1.13E-02
GO:0044444~cytoplasmic part	1.16	8.33E-04	1.11E-02
GO:0043123~positive regulation of I-kappaB kinase/NF-kappaB cascade	2.78	8.91E-04	4.07E-02
GO:0003779~actin binding	1.86	9.02E-04	4.50E-02
GO:0048545~response to steroid hormone stimulus	2.16	9.86E-04	4.43E-02
GO:0009395~phospholipid catabolic process	5.73	9.91E-04	4.39E-02
GO:0016491~oxidoreductase activity	1.55	1.02E-03	4.84E-02
GO:0016740~transferase activity	1.31	1.07E-03	4.85E-02
GO:0022603~regulation of anatomical structure morphogenesis	2.06	1.12E-03	4.87E-02
GO:0051704~multi-organism process	1.53	1.19E-03	5.13E-02
GO:0044433~cytoplasmic vesicle part	2.17	1.21E-03	1.55E-02
GO:0051051~negative regulation of transport	2.40	1.29E-03	5.45E-02
GO:0032101~regulation of response to external stimulus	2.26	1.30E-03	5.42E-02
GO:0031667~response to nutrient levels	2.10	1.38E-03	5.70E-02
GO:0031988~membrane-bounded vesicle	1.59	1.41E-03	1.76E-02
GO:0005794~Golgi apparatus	1.46	1.42E-03	1.73E-02
GO:0005506~iron ion binding	1.85	1.45E-03	6.25E-02
GO:0051707~response to other organism	1.86	1.55E-03	6.27E-02
GO:0006685~sphingomyelin catabolic process	14.40	1.56E-03	6.26E-02
GO:0010646~regulation of cell communication	1.41	1.60E-03	6.31E-02
GO:0006643~membrane lipid metabolic process	2.89	1.62E-03	6.32E-02
GO:0045177~apical part of cell	2.17	1.66E-03	1.96E-02
GO:0022607~cellular component assembly	1.44	1.66E-03	6.40E-02
GO:0005975~carbohydrate metabolic process	1.60	1.70E-03	6.49E-02
GO:0033559~unsaturated fatty acid metabolic process	3.53	1.77E-03	6.65E-02
GO:0030198~extracellular matrix organization	2.60	1.77E-03	6.60E-02
GO:0030149~sphingolipid catabolic process	6.35	1.83E-03	6.74E-02
GO:0046466~membrane lipid catabolic process	6.35	1.83E-03	6.74E-02
GO:0009967~positive regulation of signal transduction	1.83	2.00E-03	7.25E-02
GO:0005624~membrane fraction	1.46	2.13E-03	2.44E-02
GO:0007010~cytoskeleton organization	1.65	2.19E-03	7.83E-02
GO:0016324~apical plasma membrane	2.36	2.20E-03	2.46E-02
GO:0032432~actin filament bundle	4.97	2.22E-03	2.42E-02
GO:0045765~regulation of angiogenesis	3.14	2.30E-03	8.10E-02

GO:0051271~negative regulation of cell motion	3.14	2.30E-03	8.10E-02
GO:0010740~positive regulation of protein kinase cascade	2.16	2.30E-03	8.04E-02
GO:0004175~endopeptidase activity	1.71	2.40E-03	9.71E-02
GO:0006916~anti-apoptosis	2.01	2.44E-03	8.42E-02
GO:0040008~regulation of growth	1.74	2.54E-03	8.64E-02
GO:0009991~response to extracellular stimulus	1.96	2.60E-03	8.74E-02
GO:0006955~immune response	1.49	2.62E-03	8.72E-02
GO:0009607~response to biotic stimulus	1.69	2.66E-03	8.78E-02
GO:0007626~locomotory behavior	1.84	2.71E-03	8.85E-02
GO:0042641~actomyosin	4.79	2.73E-03	2.90E-02
GO:0031589~cell-substrate adhesion	2.57	2.91E-03	9.36E-02
GO:0051272~positive regulation of cell motion	2.57	2.91E-03	9.36E-02
GO:0040017~positive regulation of locomotion	2.57	2.91E-03	9.36E-02
GO:0043277~apoptotic cell clearance	12.00	3.00E-03	9.54E-02
GO:0005604~basement membrane	2.84	3.05E-03	3.15E-02
GO:0016043~cellular component organization	1.22	3.14E-03	9.88E-02
GO:0008233~peptidase activity	1.53	3.31E-03	1.27E-01
GO:0042742~defense response to bacterium	2.41	3.56E-03	1.10E-01
GO:0030335~positive regulation of cell migration	2.63	3.62E-03	1.11E-01
GO:0000267~cell fraction	1.36	3.75E-03	3.78E-02
GO:0034330~cell junction organization	3.16	3.89E-03	1.18E-01
GO:0008092~cytoskeletal protein binding	1.57	3.93E-03	1.43E-01
GO:0005539~glycosaminoglycan binding	2.23	3.93E-03	1.39E-01
GO:0043067~regulation of programmed cell death	1.42	4.05E-03	1.21E-01
GO:0030141~secretory granule	2.05	4.05E-03	3.99E-02
GO:0006633~fatty acid biosynthetic process	2.73	4.08E-03	1.21E-01
GO:0043062~extracellular structure organization	2.10	4.08E-03	1.20E-01
GO:0007596~blood coagulation	2.47	4.13E-03	1.20E-01
GO:0050817~coagulation	2.47	4.13E-03	1.20E-01
GO:0044420~extracellular matrix part	2.37	4.24E-03	4.08E-02
GO:0042493~response to drug	1.92	4.36E-03	1.25E-01
GO:0046906~tetrapyrrole binding	2.28	4.37E-03	1.48E-01
GO:0001503~ossification	2.35	4.53E-03	1.29E-01
GO:0009966~regulation of signal transduction	1.39	4.63E-03	1.30E-01
GO:0010941~regulation of cell death	1.41	4.64E-03	1.29E-01
GO:0019842~vitamin binding	2.26	4.70E-03	1.53E-01
GO:0007584~response to nutrient	2.19	4.71E-03	1.30E-01
GO:0004767~sphingomyelin phosphodiesterase activity	10.49	4.76E-03	1.50E-01
GO:0005125~cytokine activity	1.98	4.81E-03	1.47E-01
GO:0022804~active transmembrane transporter activity	1.67	4.96E-03	1.47E-01
GO:0006024~glycosaminoglycan biosynthetic process	5.14	5.01E-03	1.37E-01
GO:0034446~substrate adhesion-dependent cell spreading	10.29	5.03E-03	1.36E-01
GO:0007229~integrin-mediated signaling pathway	2.83	5.03E-03	1.35E-01
GO:0042981~regulation of apoptosis	1.41	5.17E-03	1.37E-01
GO:0048518~positive regulation of biological process	1.23	5.44E-03	1.43E-01
GO:0046394~carboxylic acid biosynthetic process	2.09	5.54E-03	1.44E-01
GO:0016053~organic acid biosynthetic process	2.09	5.54E-03	1.44E-01
GO:0022892~substrate-specific transporter activity	1.37	5.73E-03	1.63E-01
GO:0031347~regulation of defense response	2.14	5.79E-03	1.49E-01
GO:0002683~negative regulation of immune system process	2.60	5.97E-03	1.52E-01
GO:0020037~heme binding	2.28	6.05E-03	1.67E-01
GO:0051260~protein homooligomerization	2.46	6.17E-03	1.56E-01
GO:0016051~carbohydrate biosynthetic process	2.36	6.21E-03	1.56E-01
GO:0042802~identical protein binding	1.46	6.24E-03	1.67E-01

GO:0022891~substrate-specific transmembrane transporter activity	1.40	6.26E-03	1.63E-01
GO:0030148~sphingolipid biosynthetic process	4.07	6.39E-03	1.58E-01
GO:0007599~hemostasis	2.33	6.72E-03	1.65E-01
GO:0031349~positive regulation of defense response	2.71	6.79E-03	1.65E-01
GO:0050896~response to stimulus	1.16	6.91E-03	1.66E-01
GO:0016504~peptidase activator activity	4.79	6.99E-03	1.76E-01
GO:0014070~response to organic cyclic substance	2.23	7.11E-03	1.70E-01
GO:0009653~anatomical structure morphogenesis	1.31	7.31E-03	1.73E-01
GO:0004372~glycine hydroxymethyltransferase activity	9.18	7.31E-03	1.79E-01
GO:0030228~lipoprotein receptor activity	6.12	7.34E-03	1.75E-01
GO:0044432~endoplasmic reticulum part	1.65	7.54E-03	7.00E-02
GO:0007044~cell-substrate junction assembly	4.70	7.56E-03	1.77E-01
GO:0006023~aminoglycan biosynthetic process	4.70	7.56E-03	1.77E-01
GO:0031252~cell leading edge	2.14	7.69E-03	6.99E-02
GO:0019637~organophosphate metabolic process	1.89	7.77E-03	1.80E-01
GO:0052547~regulation of peptidase activity	2.51	7.79E-03	1.79E-01
GO:0043066~negative regulation of apoptosis	1.63	8.00E-03	1.82E-01
GO:0016298~lipase activity	2.39	8.00E-03	1.85E-01
GO:0060348~bone development	2.20	8.19E-03	1.85E-01
GO:0005198~structural molecule activity	1.45	8.20E-03	1.85E-01
GO:0001725~stress fiber	4.62	8.21E-03	7.30E-02
GO:0032103~positive regulation of response to external stimulus	2.81	8.44E-03	1.89E-01
GO:0004768~stearoyl-CoA 9-desaturase activity	18.36	8.55E-03	1.89E-01
GO:0005905~coated pit	3.36	8.76E-03	7.61E-02
GO:0009308~amine metabolic process	1.58	8.79E-03	1.95E-01
GO:0034599~cellular response to oxidative stress	3.35	8.83E-03	1.94E-01
GO:0030054~cell junction	1.50	9.17E-03	7.80E-02
GO:0022857~transmembrane transporter activity	1.36	9.17E-03	1.97E-01
GO:0006644~phospholipid metabolic process	1.90	9.32E-03	2.02E-01
GO:0006022~aminoglycan metabolic process	2.77	9.32E-03	2.01E-01
GO:0044431~Golgi apparatus part	1.70	9.48E-03	7.90E-02
GO:0043069~negative regulation of programmed cell death	1.60	9.57E-03	2.05E-01
GO:0001871~pattern binding	2.03	9.74E-03	2.03E-01
GO:0030247~polysaccharide binding	2.03	9.74E-03	2.03E-01
GO:0050793~regulation of developmental process	1.42	9.85E-03	2.09E-01
GO:0008201~heparin binding	2.32	1.00E-02	2.05E-01
GO:0060548~negative regulation of cell death	1.60	1.00E-02	2.11E-01
GO:0005520~insulin-like growth factor binding	4.41	1.01E-02	2.02E-01
GO:0010035~response to inorganic substance	1.84	1.01E-02	2.10E-01
GO:0006636~unsaturated fatty acid biosynthetic process	3.71	1.01E-02	2.10E-01
GO:0046467~membrane lipid biosynthetic process	3.71	1.01E-02	2.10E-01
GO:0043256~laminin complex	8.21	1.03E-02	8.44E-02
GO:0030203~glycosaminoglycan metabolic process	2.95	1.04E-02	2.14E-01
GO:0070011~peptidase activity, acting on L-amino acid peptides	1.47	1.05E-02	2.04E-01
GO:0003810~protein-glutamine gamma-glutamyltransferase activity	8.16	1.05E-02	2.02E-01
GO:0008361~regulation of cell size	1.84	1.06E-02	2.16E-01
GO:0008034~lipoprotein binding	3.67	1.07E-02	2.01E-01
GO:0048730~epidermis morphogenesis	4.32	1.09E-02	2.20E-01
GO:0008283~cell proliferation	1.53	1.10E-02	2.21E-01
GO:0042447~hormone catabolic process	8.00	1.11E-02	2.21E-01
GO:0000271~polysaccharide biosynthetic process	3.20	1.13E-02	2.23E-01
GO:0005605~basal lamina	5.43	1.15E-02	9.15E-02
GO:0050878~regulation of body fluid levels	2.04	1.16E-02	2.26E-01
GO:0009408~response to heat	2.89	1.16E-02	2.25E-01

GO:0007586~digestion	2.37	1.18E-02	2.27E-01
GO:0002376~immune system process	1.32	1.21E-02	2.32E-01
GO:0030308~negative regulation of cell growth	2.35	1.27E-02	2.40E-01
GO:0048660~regulation of smooth muscle cell proliferation	3.13	1.27E-02	2.39E-01
GO:0010810~regulation of cell-substrate adhesion	3.13	1.27E-02	2.39E-01
GO:0045787~positive regulation of cell cycle	2.84	1.28E-02	2.40E-01
GO:0050900~leukocyte migration	2.84	1.28E-02	2.40E-01
GO:0044085~cellular component biogenesis	1.31	1.31E-02	2.43E-01
GO:0009628~response to abiotic stimulus	1.57	1.34E-02	2.47E-01
GO:0007517~muscle organ development	1.79	1.36E-02	2.47E-01
GO:0009986~cell surface	1.59	1.37E-02	1.06E-01
GO:0016877~ligase activity, forming carbon-sulfur bonds	4.08	1.40E-02	2.51E-01
GO:0006690~icosanoid metabolic process	3.06	1.42E-02	2.57E-01
GO:0008284~positive regulation of cell proliferation	1.52	1.43E-02	2.56E-01
GO:0042445~hormone metabolic process	2.21	1.43E-02	2.55E-01
GO:0004653~polypeptide N-acetylgalactosaminyltransferase activity	5.10	1.44E-02	2.53E-01
GO:0004467~long-chain-fatty-acid-CoA ligase activity	7.34	1.44E-02	2.49E-01
GO:0009101~glycoprotein biosynthetic process	1.94	1.45E-02	2.56E-01
GO:0006461~protein complex assembly	1.46	1.46E-02	2.57E-01
GO:0070271~protein complex biogenesis	1.46	1.46E-02	2.57E-01
GO:0019221~cytokine-mediated signaling pathway	2.57	1.49E-02	2.59E-01
GO:0001952~regulation of cell-matrix adhesion	4.00	1.51E-02	2.62E-01
GO:0007167~enzyme linked receptor protein signaling pathway	1.58	1.52E-02	2.62E-01
GO:0033138~positive regulation of peptidyl-serine phosphorylation	7.20	1.52E-02	2.61E-01
GO:0045745~positive regulation of G-protein coupled receptor protein signaling	7.20	1.52E-02	2.61E-01
GO:0005215~transporter activity	1.28	1.53E-02	2.57E-01
GO:0033993~response to lipid	5.00	1.54E-02	2.62E-01
GO:0007492~endoderm development	5.00	1.54E-02	2.62E-01
GO:0044262~cellular carbohydrate metabolic process	1.54	1.56E-02	2.64E-01
GO:0048522~positive regulation of cellular process	1.21	1.57E-02	2.64E-01
GO:0005254~chloride channel activity	2.55	1.58E-02	2.60E-01
GO:0051346~negative regulation of hydrolase activity	3.00	1.59E-02	2.65E-01
GO:0004497~monooxygenase activity	2.27	1.62E-02	2.61E-01
GO:0050678~regulation of epithelial cell proliferation	2.54	1.62E-02	2.68E-01
GO:0009266~response to temperature stimulus	2.39	1.62E-02	2.68E-01
GO:0030017~sarcomere	2.26	1.66E-02	1.26E-01
GO:0030016~myofibril	2.16	1.67E-02	1.24E-01
GO:0009100~glycoprotein metabolic process	1.78	1.71E-02	2.78E-01
GO:0002020~protease binding	4.83	1.75E-02	2.76E-01
GO:0048732~gland development	2.00	1.77E-02	2.85E-01
GO:0051604~protein maturation	2.07	1.77E-02	2.84E-01
GO:0019898~extrinsic to membrane	1.46	1.79E-02	1.30E-01
GO:0080134~regulation of response to stress	1.64	1.80E-02	2.87E-01
GO:0030246~carbohydrate binding	1.56	1.82E-02	2.81E-01
GO:0012505~endomembrane system	1.35	1.85E-02	1.32E-01
GO:0000038~very-long-chain fatty acid metabolic process	4.74	1.87E-02	2.94E-01
GO:0007565~female pregnancy	2.13	1.88E-02	2.94E-01
GO:0044449~contractile fiber part	2.13	1.90E-02	1.34E-01
GO:0005041~low-density lipoprotein receptor activity	6.67	1.91E-02	2.88E-01
GO:0015645~fatty-acid ligase activity	6.67	1.91E-02	2.88E-01
GO:0015629~actin cytoskeleton	1.65	1.99E-02	1.37E-01
GO:0006684~sphingomyelin metabolic process	6.55	2.01E-02	3.10E-01
GO:0007567~parturition	6.55	2.01E-02	3.10E-01
GO:0050921~positive regulation of chemotaxis	3.73	2.03E-02	3.11E-01

GO:0010817~regulation of hormone levels	1.91	2.05E-02	3.13E-01
GO:0004364~glutathione transferase activity	4.59	2.10E-02	3.07E-01
GO:0045792~negative regulation of cell size	2.18	2.11E-02	3.18E-01
GO:0016485~protein processing	2.09	2.13E-02	3.20E-01
GO:0048646~anatomical structure formation involved in morphogenesis	1.53	2.16E-02	3.21E-01
GO:0016712~oxidoreductase activity, acting on paired donors, with incorporatic	3.67	2.16E-02	3.11E-01
GO:0050830~defense response to Gram-positive bacterium	4.50	2.23E-02	3.30E-01
GO:0030879~mammary gland development	2.57	2.25E-02	3.30E-01
GO:0043235~receptor complex	2.07	2.29E-02	1.54E-01
GO:0016757~transferase activity, transferring glycosyl groups	1.65	2.31E-02	3.24E-01
GO:0051716~cellular response to stimulus	1.32	2.32E-02	3.36E-01
GO:0007566~embryo implantation	3.60	2.33E-02	3.37E-01
GO:0050729~positive regulation of inflammatory response	3.60	2.33E-02	3.37E-01
GO:0043434~response to peptide hormone stimulus	1.87	2.41E-02	3.44E-01
GO:0050727~regulation of inflammatory response	2.37	2.43E-02	3.46E-01
GO:0034329~cell junction assembly	3.07	2.44E-02	3.46E-01
GO:0015296~anion:cation symporter activity	3.55	2.47E-02	3.38E-01
GO:0030169~low-density lipoprotein binding	4.37	2.48E-02	3.35E-01
GO:0031406~carboxylic acid binding	1.91	2.52E-02	3.35E-01
GO:0007160~cell-matrix adhesion	2.23	2.53E-02	3.54E-01
GO:0005253~anion channel activity	2.35	2.54E-02	3.33E-01
GO:0010888~negative regulation of lipid storage	6.00	2.57E-02	3.57E-01
GO:0008654~phospholipid biosynthetic process	2.12	2.57E-02	3.56E-01
GO:0019835~cytolysis	4.29	2.64E-02	3.62E-01
GO:0010883~regulation of lipid storage	4.29	2.64E-02	3.62E-01
GO:0010466~negative regulation of peptidase activity	4.29	2.64E-02	3.62E-01
GO:0048661~positive regulation of smooth muscle cell proliferation	3.48	2.65E-02	3.62E-01
GO:0050920~regulation of chemotaxis	3.48	2.65E-02	3.62E-01
GO:0033189~response to vitamin A	3.00	2.72E-02	3.68E-01
GO:0030307~positive regulation of cell growth	3.00	2.72E-02	3.68E-01
GO:0014812~muscle cell migration	10.80	2.75E-02	3.69E-01
GO:0032695~negative regulation of interleukin-12 production	10.80	2.75E-02	3.69E-01
GO:0032535~regulation of cellular component size	1.59	2.81E-02	3.74E-01
GO:0005788~endoplasmic reticulum lumen	2.31	2.83E-02	1.85E-01
GO:0032355~response to estradiol stimulus	2.67	2.88E-02	3.80E-01
GO:0042476~odontogenesis	2.67	2.88E-02	3.80E-01
GO:0008219~cell death	1.33	2.92E-02	3.83E-01
GO:0016787~hydrolase activity	1.17	2.92E-02	3.68E-01
GO:0008509~anion transmembrane transporter activity	1.87	2.94E-02	3.66E-01
GO:0007610~behavior	1.42	3.01E-02	3.91E-01
GO:0042303~molting cycle	2.93	3.02E-02	3.91E-01
GO:0007162~negative regulation of cell adhesion	2.93	3.02E-02	3.91E-01
GO:0042633~hair cycle	2.93	3.02E-02	3.91E-01
GO:0034097~response to cytokine stimulus	2.28	3.04E-02	3.91E-01
GO:0043292~contractile fiber	1.99	3.06E-02	1.96E-01
GO:0016021~integral to membrane	1.09	3.09E-02	1.94E-01
GO:0045596~negative regulation of cell differentiation	1.67	3.16E-02	4.02E-01
GO:0051093~negative regulation of developmental process	1.60	3.16E-02	4.00E-01
GO:0033674~positive regulation of kinase activity	1.64	3.24E-02	4.06E-01
GO:0016265~death	1.32	3.27E-02	4.08E-01
GO:0032870~cellular response to hormone stimulus	1.90	3.33E-02	4.12E-01
GO:0031669~cellular response to nutrient levels	2.86	3.34E-02	4.12E-01
GO:0015294~solute:cation symporter activity	2.13	3.36E-02	4.02E-01
GO:0015291~secondary active transmembrane transporter activity	1.68	3.36E-02	3.98E-01

GO:0016337~cell-cell adhesion	1.57	3.38E-02	4.13E-01
GO:0031224~intrinsic to membrane	1.09	3.41E-02	2.10E-01
GO:0048771~tissue remodeling	2.57	3.43E-02	4.17E-01
GO:0030027~lamellipodium	2.38	3.46E-02	2.09E-01
GO:0051179~localization	1.13	3.48E-02	4.21E-01
GO:0051336~regulation of hydrolase activity	1.50	3.51E-02	4.22E-01
GO:0051239~regulation of multicellular organismal process	1.27	3.54E-02	4.23E-01
GO:0001525~angiogenesis	1.82	3.55E-02	4.22E-01
GO:0006066~alcohol metabolic process	1.43	3.56E-02	4.21E-01
GO:0015293~symporter activity	1.88	3.59E-02	4.14E-01
GO:0004620~phospholipase activity	2.21	3.61E-02	4.11E-01
GO:0065003~macromolecular complex assembly	1.33	3.66E-02	4.29E-01
GO:0010627~regulation of protein kinase cascade	1.59	3.68E-02	4.29E-01
GO:0043433~negative regulation of transcription factor activity	2.80	3.68E-02	4.28E-01
GO:0010324~membrane invagination	1.64	3.71E-02	4.29E-01
GO:0006897~endocytosis	1.64	3.71E-02	4.29E-01
GO:0030336~negative regulation of cell migration	2.53	3.73E-02	4.30E-01
GO:0052548~regulation of endopeptidase activity	2.20	3.74E-02	4.29E-01
GO:0044057~regulation of system process	1.51	3.76E-02	4.29E-01
GO:0002682~regulation of immune system process	1.45	3.78E-02	4.30E-01
GO:0022600~digestive system process	3.18	3.80E-02	4.30E-01
GO:0048520~positive regulation of behavior	3.18	3.80E-02	4.30E-01
GO:0050291~sphingosine N-acyltransferase activity	9.18	3.83E-02	4.26E-01
GO:0033135~regulation of peptidyl-serine phosphorylation	5.14	3.91E-02	4.38E-01
GO:0001944~vasculature development	1.58	3.96E-02	4.41E-01
GO:0019369~arachidonic acid metabolic process	9.00	3.97E-02	4.40E-01
GO:0043549~regulation of kinase activity	1.46	4.08E-02	4.48E-01
GO:0044430~cytoskeletal part	1.26	4.08E-02	2.40E-01
GO:0045926~negative regulation of growth	1.96	4.15E-02	4.52E-01
GO:0045860~positive regulation of protein kinase activity	1.61	4.17E-02	4.52E-01
GO:0042803~protein homodimerization activity	1.48	4.18E-02	4.50E-01
GO:0042327~positive regulation of phosphorylation	2.04	4.24E-02	4.56E-01
GO:0034754~cellular hormone metabolic process	2.44	4.38E-02	4.67E-01
GO:0005976~polysaccharide metabolic process	1.95	4.38E-02	4.65E-01
GO:0005518~collagen binding	3.06	4.39E-02	4.62E-01
GO:0070330~aromatase activity	3.67	4.43E-02	4.60E-01
GO:0005355~glucose transmembrane transporter activity	4.89	4.47E-02	4.60E-01
GO:0045859~regulation of protein kinase activity	1.46	4.49E-02	4.72E-01
GO:0000139~Golgi membrane	1.69	4.49E-02	2.58E-01
GO:0051347~positive regulation of transferase activity	1.58	4.54E-02	4.74E-01
GO:0048514~blood vessel morphogenesis	1.62	4.60E-02	4.78E-01
GO:0001893~maternal placenta development	4.80	4.69E-02	4.83E-01
GO:0050777~negative regulation of immune response	3.60	4.69E-02	4.82E-01
GO:0006493~protein amino acid O-linked glycosylation	3.60	4.69E-02	4.82E-01
GO:0043933~macromolecular complex subunit organization	1.29	4.78E-02	4.87E-01
GO:0045793~positive regulation of cell size	2.63	4.83E-02	4.89E-01
GO:0002237~response to molecule of bacterial origin	2.09	4.84E-02	4.89E-01
GO:0042379~chemokine receptor binding	2.62	4.87E-02	4.85E-01
GO:0016765~transferase activity, transferring alkyl or aryl (other than methyl) gr	2.62	4.87E-02	4.85E-01
GO:0042995~cell projection	1.30	4.90E-02	2.75E-01
GO:0045937~positive regulation of phosphate metabolic process	1.98	5.04E-02	5.01E-01
GO:0010562~positive regulation of phosphorus metabolic process	1.98	5.04E-02	5.01E-01
GO:0040013~negative regulation of locomotion	2.36	5.10E-02	5.04E-01
GO:0005606~laminin-1 complex	7.92	5.11E-02	2.82E-01

GO:0004908~interleukin-1 receptor activity	7.87	5.17E-02	5.02E-01
GO:0001968~fibronectin binding	7.87	5.17E-02	5.02E-01
GO:0017166~vinculin binding	7.87	5.17E-02	5.02E-01
GO:0030595~leukocyte chemotaxis	2.92	5.20E-02	5.09E-01
GO:0051971~positive regulation of transmission of nerve impulse	2.92	5.20E-02	5.09E-01
GO:0051240~positive regulation of multicellular organismal process	1.55	5.21E-02	5.09E-01
GO:0005911~cell-cell junction	1.65	5.26E-02	2.85E-01
GO:0005149~interleukin-1 receptor binding	4.59	5.29E-02	5.05E-01
GO:0045595~regulation of cell differentiation	1.35	5.33E-02	5.15E-01
GO:0005504~fatty acid binding	2.90	5.35E-02	5.05E-01
GO:0032494~response to peptidoglycan	7.72	5.36E-02	5.16E-01
GO:0007435~salivary gland morphogenesis	7.72	5.36E-02	5.16E-01
GO:0001568~blood vessel development	1.54	5.36E-02	5.15E-01
GO:0000302~response to reactive oxygen species	2.16	5.54E-02	5.25E-01
GO:0002698~negative regulation of immune effector process	4.50	5.55E-02	5.25E-01
GO:0031532~actin cytoskeleton reorganization	4.50	5.55E-02	5.25E-01
GO:0022612~gland morphogenesis	4.50	5.55E-02	5.25E-01
GO:0050840~extracellular matrix binding	3.40	5.64E-02	5.19E-01
GO:0015718~monocarboxylic acid transport	2.52	5.71E-02	5.34E-01
GO:0009055~electron carrier activity	1.58	5.73E-02	5.20E-01
GO:0043167~ion binding	1.09	5.82E-02	5.22E-01
GO:0008374~O-acyltransferase activity	2.82	5.88E-02	5.21E-01
GO:0015082~di-, tri-valent inorganic cation transmembrane transporter activity	2.82	5.88E-02	5.21E-01
GO:0044242~cellular lipid catabolic process	2.13	5.90E-02	5.45E-01
GO:0051090~regulation of transcription factor activity	1.92	5.94E-02	5.46E-01
GO:0007178~transmembrane receptor protein serine/threonine kinase signaling	1.92	5.94E-02	5.46E-01
GO:0007050~cell cycle arrest	1.92	5.94E-02	5.46E-01
GO:0022604~regulation of cell morphogenesis	1.79	5.99E-02	5.47E-01
GO:0042325~regulation of phosphorylation	1.35	6.06E-02	5.50E-01
GO:0043392~negative regulation of DNA binding	2.47	6.18E-02	5.56E-01
GO:0042612~MHC class I protein complex	3.30	6.19E-02	3.24E-01
GO:0051338~regulation of transferase activity	1.40	6.21E-02	5.56E-01
GO:0043168~anion binding	2.00	6.24E-02	5.39E-01
GO:0051049~regulation of transport	1.37	6.26E-02	5.58E-01
GO:0017015~regulation of transforming growth factor beta receptor signaling pa	2.77	6.28E-02	5.58E-01
GO:0060326~cell chemotaxis	2.77	6.28E-02	5.58E-01
GO:0031646~positive regulation of neurological system process	2.77	6.28E-02	5.58E-01
GO:0032496~response to lipopolysaccharide	2.10	6.28E-02	5.57E-01
GO:0031668~cellular response to extracellular stimulus	2.25	6.32E-02	5.57E-01
GO:0048167~regulation of synaptic plasticity	2.25	6.32E-02	5.57E-01
GO:0046983~protein dimerization activity	1.32	6.46E-02	5.48E-01
GO:0042177~negative regulation of protein catabolic process	4.24	6.47E-02	5.65E-01
GO:0051969~regulation of transmission of nerve impulse	1.71	6.48E-02	5.64E-01
GO:0046942~carboxylic acid transport	1.71	6.48E-02	5.64E-01
GO:0048584~positive regulation of response to stimulus	1.53	6.61E-02	5.70E-01
GO:0005381~iron ion transmembrane transporter activity	6.88	6.66E-02	5.55E-01
GO:0016702~oxidoreductase activity, acting on single donors with incorporation	2.22	6.66E-02	5.51E-01
GO:0051213~dioxygenase activity	2.22	6.66E-02	5.51E-01
GO:0010811~positive regulation of cell-substrate adhesion	3.22	6.68E-02	5.73E-01
GO:0032963~collagen metabolic process	3.22	6.68E-02	5.73E-01
GO:0051099~positive regulation of binding	2.08	6.68E-02	5.71E-01
GO:0051015~actin filament binding	2.42	6.68E-02	5.48E-01
GO:0019220~regulation of phosphate metabolic process	1.34	6.75E-02	5.74E-01
GO:0051174~regulation of phosphorus metabolic process	1.34	6.75E-02	5.74E-01

GO:0042063~gliogenesis	2.22	6.76E-02	5.73E-01
GO:0015849~organic acid transport	1.70	6.76E-02	5.72E-01
GO:0022890~inorganic cation transmembrane transporter activity	1.70	6.82E-02	5.52E-01
GO:0005793~ER-Golgi intermediate compartment	2.70	6.86E-02	3.49E-01
GO:0005884~actin filament	2.70	6.86E-02	3.49E-01
GO:0055093~response to hyperoxia	6.75	6.89E-02	5.78E-01
GO:0032026~response to magnesium ion	6.75	6.89E-02	5.78E-01
GO:0008347~glial cell migration	6.75	6.89E-02	5.78E-01
GO:0001919~regulation of receptor recycling	6.75	6.89E-02	5.78E-01
GO:0050746~regulation of lipoprotein metabolic process	6.75	6.89E-02	5.78E-01
GO:0045737~positive regulation of cyclin-dependent protein kinase activity	6.75	6.89E-02	5.78E-01
GO:0007184~SMAD protein nuclear translocation	6.75	6.89E-02	5.78E-01
GO:0001726~ruffle	2.21	6.92E-02	3.48E-01
GO:0043281~regulation of caspase activity	2.05	7.09E-02	5.87E-01
GO:0016701~oxidoreductase activity, acting on single donors with incorporation	2.19	7.11E-02	5.63E-01
GO:0004714~transmembrane receptor protein tyrosine kinase activity	2.19	7.11E-02	5.63E-01
GO:0015149~hexose transmembrane transporter activity	4.08	7.12E-02	5.60E-01
GO:0051241~negative regulation of multicellular organismal process	1.65	7.18E-02	5.91E-01
GO:0050790~regulation of catalytic activity	1.23	7.23E-02	5.92E-01
GO:0005829~cytosol	1.18	7.24E-02	3.57E-01
GO:0015698~inorganic anion transport	1.94	7.25E-02	5.92E-01
GO:0051101~regulation of DNA binding	1.79	7.26E-02	5.91E-01
GO:0042692~muscle cell differentiation	1.79	7.26E-02	5.91E-01
GO:0015758~glucose transport	3.10	7.42E-02	5.98E-01
GO:0043588~skin development	3.10	7.42E-02	5.98E-01
GO:0009595~detection of biotic stimulus	4.00	7.45E-02	5.98E-01
GO:0050679~positive regulation of epithelial cell proliferation	2.63	7.48E-02	5.98E-01
GO:0050818~regulation of coagulation	2.63	7.48E-02	5.98E-01
GO:0051092~positive regulation of NF-kappaB transcription factor activity	2.63	7.48E-02	5.98E-01
GO:0045017~glycerolipid biosynthetic process	2.03	7.51E-02	5.99E-01
GO:0031674~I band	2.35	7.53E-02	3.65E-01
GO:0045178~basal part of cell	3.08	7.62E-02	3.65E-01
GO:0060541~respiratory system development	1.83	7.66E-02	6.05E-01
GO:0009897~external side of plasma membrane	1.63	7.70E-02	3.64E-01
GO:0004896~cytokine receptor activity	2.34	7.72E-02	5.86E-01
GO:0015144~carbohydrate transmembrane transporter activity	3.06	7.77E-02	5.85E-01
GO:0016769~transferase activity, transferring nitrogenous groups	3.06	7.77E-02	5.85E-01
GO:0016044~membrane organization	1.37	7.82E-02	6.11E-01
GO:0001664~G-protein-coupled receptor binding	1.82	8.01E-02	5.93E-01
GO:0031090~organelle membrane	1.20	8.03E-02	3.73E-01
GO:0043583~ear development	1.90	8.05E-02	6.21E-01
GO:0030173~integral to Golgi membrane	2.58	8.08E-02	3.71E-01
GO:0065009~regulation of molecular function	1.21	8.11E-02	6.23E-01
GO:0001942~hair follicle development	2.57	8.12E-02	6.22E-01
GO:0022404~molting cycle process	2.57	8.12E-02	6.22E-01
GO:0042594~response to starvation	2.57	8.12E-02	6.22E-01
GO:0006672~ceramide metabolic process	2.57	8.12E-02	6.22E-01
GO:0022405~hair cycle process	2.57	8.12E-02	6.22E-01
GO:0016755~transferase activity, transferring amino-acyl groups	3.86	8.13E-02	5.94E-01
GO:0016628~oxidoreductase activity, acting on the CH-CH group of donors, NAD	3.86	8.13E-02	5.94E-01
GO:0015145~monosaccharide transmembrane transporter activity	3.86	8.13E-02	5.94E-01
GO:0031430~M band	6.16	8.16E-02	3.71E-01
GO:0034614~cellular response to reactive oxygen species	3.00	8.21E-02	6.25E-01
GO:0008645~hexose transport	3.00	8.21E-02	6.25E-01

GO:0045768~positive regulation of anti-apoptosis	3.00	8.21E-02	6.25E-01
GO:0042834~peptidoglycan binding	6.12	8.26E-02	5.97E-01
GO:0051098~regulation of binding	1.65	8.30E-02	6.28E-01
GO:0031644~regulation of neurological system process	1.65	8.30E-02	6.28E-01
GO:0031069~hair follicle morphogenesis	3.79	8.50E-02	6.36E-01
GO:0051402~neuron apoptosis	3.79	8.50E-02	6.36E-01
GO:0002218~activation of innate immune response	3.79	8.50E-02	6.36E-01
GO:0002758~innate immune response-activating signal transduction	3.79	8.50E-02	6.36E-01
GO:0019432~triglyceride biosynthetic process	6.00	8.54E-02	6.36E-01
GO:0031638~zymogen activation	6.00	8.54E-02	6.36E-01
GO:0016045~detection of bacterium	6.00	8.54E-02	6.36E-01
GO:0008376~acetylgalactosaminyltransferase activity	2.96	8.55E-02	6.06E-01
GO:0019899~enzyme binding	1.30	8.59E-02	6.04E-01
GO:0007423~sensory organ development	1.49	8.61E-02	6.38E-01
GO:0008081~phosphoric diester hydrolase activity	1.97	8.61E-02	6.02E-01
GO:0042611~MHC protein complex	2.27	8.63E-02	3.84E-01
GO:0045121~membrane raft	1.68	8.66E-02	3.82E-01
GO:0043169~cation binding	1.08	8.68E-02	6.01E-01
GO:0005768~endosome	1.41	8.70E-02	3.80E-01
GO:0034637~cellular carbohydrate biosynthetic process	2.09	8.70E-02	6.41E-01
GO:0042175~nuclear envelope-endoplasmic reticulum network	1.43	8.91E-02	3.84E-01
GO:0007507~heart development	1.51	8.95E-02	6.51E-01
GO:0030424~axon	1.63	8.98E-02	3.83E-01
GO:0015749~monosaccharide transport	2.90	9.03E-02	6.53E-01
GO:0043112~receptor metabolic process	2.90	9.03E-02	6.53E-01
GO:0046456~icosanoid biosynthetic process	2.90	9.03E-02	6.53E-01
GO:0044259~multicellular organismal macromolecule metabolic process	2.90	9.03E-02	6.53E-01
GO:0015075~ion transmembrane transporter activity	1.24	9.03E-02	6.13E-01
GO:0030057~desmosome	3.70	9.05E-02	3.82E-01
GO:0005789~endoplasmic reticulum membrane	1.44	9.14E-02	3.81E-01
GO:0008373~sialyltransferase activity	3.67	9.19E-02	6.16E-01
GO:0045637~regulation of myeloid cell differentiation	2.06	9.23E-02	6.60E-01
GO:0043388~positive regulation of DNA binding	2.06	9.23E-02	6.60E-01
GO:0044271~nitrogen compound biosynthetic process	1.39	9.34E-02	6.63E-01
GO:0005201~extracellular matrix structural constituent	1.92	9.56E-02	6.28E-01
GO:0051129~negative regulation of cellular component organization	1.65	9.60E-02	6.73E-01
GO:0002685~regulation of leukocyte migration	3.60	9.60E-02	6.72E-01
GO:0032570~response to progesterone stimulus	3.60	9.60E-02	6.72E-01
GO:0051248~negative regulation of protein metabolic process	1.54	9.67E-02	6.73E-01
GO:0005938~cell cortex	1.65	9.74E-02	3.98E-01
GO:0030324~lung development	1.82	9.81E-02	6.77E-01
GO:0006486~protein amino acid glycosylation	1.69	9.86E-02	6.78E-01
GO:0070085~glycosylation	1.69	9.86E-02	6.78E-01
GO:0043413~biopolymer glycosylation	1.69	9.86E-02	6.78E-01
GO:0009267~cellular response to starvation	2.81	9.89E-02	6.78E-01
GO:0046870~cadmium ion binding	5.51	9.96E-02	6.40E-01
GO:0017040~ceramidase activity	5.51	9.96E-02	6.40E-01
GO:0019966~interleukin-1 binding	5.51	9.96E-02	6.40E-01
GO:0030280~structural constituent of epidermis	5.51	9.96E-02	6.40E-01
GO:0006820~anion transport	1.64	9.98E-02	6.80E-01
GO:0044093~positive regulation of molecular function	1.26	9.99E-02	6.79E-01
