# Supplementary material for: Plasticity of Airway Epithelial Cell Transcriptome in Response to Flagellin
Source: PLoS One. 2015 Feb 10;10(2):e0115486. doi: 10.1371/journal.pone.0115486 (PMC4323341; doi:10.1371/journal.pone.0115486)
Supplement: S2 Table — (PDF) [file pone.0115486.s003.pdf]

Supplementary Table 2. Complete results from functional enrichment analysis of differentially expressed genes between monolayer vs. ALI AEC cultures as identified by RNAseq.

Gene Ontology Annotation	Fold Enrichment	P-value	Adjusted P-value
GO:0007398~ectoderm development	5.75	3.93E-31	1.24E-27
GO:0008544~epidermis development	5.93	1.72E-30	2.71E-27
GO:0009913~epidermal cell differentiation	7.70	2.40E-19	2.52E-16
GO:0030216~keratinocyte differentiation	7.86	2.13E-18	1.68E-15
GO:0009888~tissue development	2.58	6.19E-18	3.91E-15
GO:0030855~epithelial cell differentiation	5.09	4.53E-17	2.38E-14
GO:0048731~system development	1.68	2.76E-16	1.00E-13
GO:0048856~anatomical structure development	1.63	1.63E-15	6.57E-13
GO:0048513~organ development	1.78	8.64E-15	3.04E-12
GO:0001533~cornified envelope	13.16	1.42E-14	5.90E-12
GO:0005576~extracellular region	1.71	2.56E-14	5.30E-12
GO:0007275~multicellular organismal development	1.52	3.86E-13	1.22E-10
GO:0032502~developmental process	1.48	7.82E-13	2.24E-10
GO:0044421~extracellular region part	2.04	1.37E-12	1.89E-10
GO:0031424~keratinization	7.91	3.72E-12	9.78E-10
GO:0060429~epithelium development	3.39	4.70E-12	1.14E-09
GO:0007155~cell adhesion	2.20	6.12E-12	1.38E-09
GO:0022610~biological adhesion	2.19	6.65E-12	1.40E-09
GO:0009605~response to external stimulus	2.00	1.65E-11	3.25E-09
GO:0031012~extracellular matrix	2.62	1.02E-09	1.06E-07
GO:0009611~response to wounding	2.23	1.49E-09	2.76E-07
GO:0005578~proteinaceous extracellular matrix	2.66	2.27E-09	1.88E-07
GO:0030154~cell differentiation	1.61	3.14E-09	5.50E-07
GO:0006928~cell motion	2.26	5.48E-09	9.09E-07
GO:0070161~anchoring junction	3.26	1.80E-08	1.24E-06
GO:0004866~endopeptidase inhibitor activity	3.53	1.81E-08	1.73E-05
GO:0040011~locomotion	2.28	1.85E-08	2.91E-06
GO:0048869~cellular developmental process	1.55	2.72E-08	4.08E-06
GO:0022603~regulation of anatomical structure morphogenesis	2.86	5.36E-08	7.69E-06
GO:0018149~peptide cross-linking	8.26	5.46E-08	7.50E-06
GO:0030414~peptidase inhibitor activity	3.34	5.87E-08	2.80E-05
GO:0005509~calcium ion binding	1.79	7.60E-08	2.42E-05
GO:0016477~cell migration	2.59	7.83E-08	1.03E-05
GO:0005615~extracellular space	1.93	9.27E-08	5.49E-06
GO:0042221~response to chemical stimulus	1.62	1.48E-07	1.87E-05
GO:0005198~structural molecule activity	1.93	3.21E-07	7.67E-05
GO:0004867~serine-type endopeptidase inhibitor activity	3.97	4.41E-07	8.42E-05
GO:0051674~localization of cell	2.39	4.80E-07	5.83E-05
GO:0048870~cell motility	2.39	4.80E-07	5.83E-05
GO:0005856~cytoskeleton	1.57	4.96E-07	2.57E-05
GO:0016323~basolateral plasma membrane	2.76	7.74E-07	3.57E-05
GO:0006950~response to stress	1.49	9.21E-07	1.08E-04
GO:0042127~regulation of cell proliferation	1.75	1.68E-06	1.89E-04
GO:0004857~enzyme inhibitor activity	2.44	1.80E-06	2.86E-04
GO:0051270~regulation of cell motion	2.69	3.16E-06	3.44E-04
GO:0005925~focal adhesion	3.37	1.06E-05	4.42E-04
GO:0044459~plasma membrane part	1.37	1.10E-05	4.14E-04
GO:0005912~adherens junction	2.80	1.38E-05	4.76E-04
GO:0048519~negative regulation of biological process	1.40	1.78E-05	1.87E-03

GO:0005924~cell-substrate adherens junction	3.24	1.84E-05	5.88E-04
GO:0030155~regulation of cell adhesion	2.87	2.28E-05	2.32E-03
GO:0005102~receptor binding	1.61	3.19E-05	4.34E-03
GO:0032501~multicellular organismal process	1.22	3.45E-05	3.39E-03
GO:0070482~response to oxygen levels	2.79	3.54E-05	3.38E-03
GO:0022604~regulation of cell morphogenesis	2.87	3.76E-05	3.49E-03
GO:0009725~response to hormone stimulus	2.00	3.85E-05	3.46E-03
GO:0030055~cell-substrate junction	3.07	3.96E-05	1.17E-03
GO:0005200~structural constituent of cytoskeleton	3.70	4.06E-05	4.84E-03
GO:0048523~negative regulation of cellular process	1.40	4.62E-05	4.04E-03
GO:0019838~growth factor binding	3.13	5.19E-05	5.49E-03
GO:0030198~extracellular matrix organization	3.10	5.90E-05	5.02E-03
GO:0006629~lipid metabolic process	1.61	5.94E-05	4.93E-03
GO:0005886~plasma membrane	1.24	6.18E-05	1.71E-03
GO:0050793~regulation of developmental process	1.67	6.70E-05	5.41E-03
GO:0044420~extracellular matrix part	2.94	7.16E-05	1.86E-03
GO:0005515~protein binding	1.12	7.52E-05	7.15E-03
GO:0016020~membrane	1.14	7.56E-05	1.84E-03
GO:0005506~iron ion binding	2.08	7.66E-05	6.63E-03
GO:0005783~endoplasmic reticulum	1.55	7.94E-05	1.83E-03
GO:0005604~basement membrane	3.48	8.25E-05	1.80E-03
GO:0000904~cell morphogenesis involved in differentiation	2.20	9.78E-05	7.69E-03
GO:0007010~cytoskeleton organization	1.85	1.01E-04	7.76E-03
GO:0043062~extracellular structure organization	2.52	1.06E-04	7.97E-03
GO:0006952~defense response	1.69	1.09E-04	7.96E-03
GO:0005737~cytoplasm	1.13	1.29E-04	2.67E-03
GO:0001666~response to hypoxia	2.67	1.61E-04	1.15E-02
GO:0009719~response to endogenous stimulus	1.86	1.61E-04	1.12E-02
GO:0030054~cell junction	1.75	1.62E-04	3.19E-03
GO:0042060~wound healing	2.34	1.64E-04	1.12E-02
GO:0055114~oxidation reduction	1.65	1.65E-04	1.10E-02
GO:0031091~platelet alpha granule	3.88	2.06E-04	3.87E-03
GO:0001558~regulation of cell growth	2.31	2.08E-04	1.36E-02
GO:0016491~oxidoreductase activity	1.62	2.36E-04	1.86E-02
GO:0009653~anatomical structure morphogenesis	1.43	2.58E-04	1.65E-02
GO:0030247~polysaccharide binding	2.49	2.79E-04	2.03E-02
GO:0001871~pattern binding	2.49	2.79E-04	2.03E-02
GO:0010769~regulation of cell morphogenesis involved in differentiation	3.25	3.08E-04	1.92E-02
GO:0050771~negative regulation of axonogenesis	5.73	3.26E-04	2.00E-02
GO:0031589~cell-substrate adhesion	2.92	3.33E-04	2.00E-02
GO:0030036~actin cytoskeleton organization	2.14	3.70E-04	2.18E-02
GO:0031099~regeneration	3.37	3.92E-04	2.27E-02
GO:0016705~oxidoreductase activity, acting on paired donors, with incorporation	2.57	4.00E-04	2.69E-02
GO:0045785~positive regulation of cell adhesion	3.58	4.25E-04	2.41E-02
GO:0031100~organ regeneration	5.50	4.25E-04	2.37E-02
GO:0010033~response to organic substance	1.56	4.34E-04	2.37E-02
GO:0030334~regulation of cell migration	2.33	4.78E-04	2.57E-02
GO:0006916~anti-apoptosis	2.17	5.10E-04	2.69E-02
GO:0030057~desmosome	6.33	5.52E-04	9.91E-03
GO:0051093~negative regulation of developmental process	2.00	6.16E-04	3.19E-02
GO:0005539~glycosaminoglycan binding	2.48	6.24E-04	3.89E-02
GO:0006954~inflammatory response	1.87	6.46E-04	3.29E-02
GO:0045765~regulation of angiogenesis	3.41	6.55E-04	3.28E-02

GO:0030879~mammary gland development	3.41	6.55E-04	3.28E-02
GO:0031345~negative regulation of cell projection organization	5.11	6.93E-04	3.42E-02
GO:0043627~response to estrogen stimulus	2.73	7.05E-04	3.42E-02
GO:0030234~enzyme regulator activity	1.50	7.06E-04	4.13E-02
GO:0032355~response to estradiol stimulus	3.64	7.08E-04	3.38E-02
GO:0043256~laminin complex	10.05	9.29E-04	1.59E-02
GO:0030029~actin filament-based process	2.00	9.81E-04	4.58E-02
GO:0048638~regulation of developmental growth	3.81	1.01E-03	4.64E-02
GO:0040012~regulation of locomotion	2.14	1.07E-03	4.85E-02
GO:0050770~regulation of axonogenesis	3.45	1.10E-03	4.90E-02
GO:0008610~lipid biosynthetic process	1.83	1.17E-03	5.15E-02
GO:0051051~negative regulation of transport	2.39	1.38E-03	5.94E-02
GO:0048732~gland development	2.39	1.38E-03	5.94E-02
GO:0043066~negative regulation of apoptosis	1.77	1.42E-03	6.05E-02
GO:0032879~regulation of localization	1.55	1.49E-03	6.26E-02
GO:0042330~taxis	2.24	1.50E-03	6.22E-02
GO:0006935~chemotaxis	2.24	1.50E-03	6.22E-02
GO:0005794~Golgi apparatus	1.45	1.52E-03	2.50E-02
GO:0031093~platelet alpha granule lumen	3.97	1.57E-03	2.47E-02
GO:0010975~regulation of neuron projection development	3.07	1.61E-03	6.57E-02
GO:0030308~negative regulation of cell growth	2.72	1.73E-03	6.95E-02
GO:0009986~cell surface	1.77	1.73E-03	2.63E-02
GO:0046906~tetrapyrrole binding	2.41	1.79E-03	9.59E-02
GO:0005605~basal lamina	6.39	1.80E-03	2.63E-02
GO:0043069~negative regulation of programmed cell death	1.74	1.80E-03	7.10E-02
GO:0033559~unsaturated fatty acid metabolic process	3.51	1.85E-03	7.20E-02
GO:0060548~negative regulation of cell death	1.74	1.88E-03	7.25E-02
GO:0004252~serine-type endopeptidase activity	2.25	1.89E-03	9.55E-02
GO:0003779~actin binding	1.79	1.94E-03	9.32E-02
GO:0008285~negative regulation of cell proliferation	1.73	1.97E-03	7.48E-02
GO:0017171~serine hydrolase activity	2.13	2.02E-03	9.20E-02
GO:0050680~negative regulation of epithelial cell proliferation	5.01	2.11E-03	7.89E-02
GO:0048730~epidermis morphogenesis	5.01	2.11E-03	7.89E-02
GO:0045596~negative regulation of cell differentiation	1.99	2.22E-03	8.20E-02
GO:0007162~negative regulation of cell adhesion	3.74	2.31E-03	8.42E-02
GO:0042633~hair cycle	3.74	2.31E-03	8.42E-02
GO:0042303~molting cycle	3.74	2.31E-03	8.42E-02
GO:0048640~negative regulation of developmental growth	8.13	2.32E-03	8.35E-02
GO:0005125~cytokine activity	2.06	2.32E-03	1.00E-01
GO:0045595~regulation of cell differentiation	1.60	2.35E-03	8.38E-02
GO:0051271~negative regulation of cell motion	3.12	2.40E-03	8.46E-02
GO:0042981~regulation of apoptosis	1.45	2.43E-03	8.44E-02
GO:0048545~response to steroid hormone stimulus	2.05	2.45E-03	8.42E-02
GO:0010035~response to inorganic substance	2.01	2.49E-03	8.45E-02
GO:0060205~cytoplasmic membrane-bounded vesicle lumen	3.70	2.51E-03	3.53E-02
GO:0030182~neuron differentiation	1.63	2.65E-03	8.88E-02
GO:0016324~apical plasma membrane	2.31	2.71E-03	3.68E-02
GO:0044425~membrane part	1.11	2.74E-03	3.61E-02
GO:0010646~regulation of cell communication	1.38	2.81E-03	9.29E-02
GO:0044255~cellular lipid metabolic process	1.56	2.82E-03	9.24E-02
GO:0016717~oxidoreductase activity, acting on paired donors, with oxidation of a	12.18	2.88E-03	1.18E-01
GO:0016820~hydrolase activity, acting on acid anhydrides, catalyzing transmembı	2.47	2.89E-03	1.13E-01
GO:0007167~enzyme linked receptor protein signaling pathway	1.73	2.92E-03	9.45E-02

GO:0000902~cell morphogenesis	1.71	2.93E-03	9.38E-02
GO:0002020~protease binding	5.77	2.95E-03	1.11E-01
GO:0043296~apical junction complex	2.56	3.06E-03	3.90E-02
GO:0043067~regulation of programmed cell death	1.43	3.10E-03	9.80E-02
GO:0050768~negative regulation of neurogenesis	3.58	3.11E-03	9.75E-02
GO:0005773~vacuole	1.87	3.32E-03	4.09E-02
GO:0031102~neuron projection regeneration	7.45	3.33E-03	1.03E-01
GO:0032101~regulation of response to external stimulus	2.14	3.35E-03	1.02E-01
GO:0031983~vesicle lumen	3.54	3.36E-03	4.02E-02
GO:0045792~negative regulation of cell size	2.53	3.36E-03	1.02E-01
GO:0044444~cytoplasmic part	1.14	3.37E-03	3.92E-02
GO:0033273~response to vitamin	2.98	3.42E-03	1.02E-01
GO:0010941~regulation of cell death	1.43	3.49E-03	1.04E-01
GO:0044431~Golgi apparatus part	1.79	3.53E-03	3.99E-02
GO:0031344~regulation of cell projection organization	2.61	3.81E-03	1.11E-01
GO:0016327~apicolateral plasma membrane	2.48	3.98E-03	4.37E-02
GO:0008201~heparin binding	2.48	3.99E-03	1.42E-01
GO:0008236~serine-type peptidase activity	2.05	4.04E-03	1.38E-01
GO:0034330~cell junction organization	3.14	4.05E-03	1.17E-01
GO:0030516~regulation of axon extension	5.37	4.11E-03	1.18E-01
GO:0005911~cell-cell junction	2.00	4.22E-03	4.51E-02
GO:0008305~integrin complex	4.37	4.42E-03	4.61E-02
GO:0031175~neuron projection development	1.82	4.65E-03	1.31E-01
GO:0006979~response to oxidative stress	2.07	4.65E-03	1.30E-01
GO:0042493~response to drug	1.91	4.70E-03	1.30E-01
GO:0044271~nitrogen compound biosynthetic process	1.71	4.70E-03	1.29E-01
GO:0010721~negative regulation of cell development	3.35	4.71E-03	1.28E-01
GO:0051346~negative regulation of hydrolase activity	3.35	4.71E-03	1.28E-01
GO:0048666~neuron development	1.69	4.73E-03	1.27E-01
GO:0045177~apical part of cell	2.02	4.76E-03	4.83E-02
GO:0017166~vinculin binding	10.44	4.83E-03	1.57E-01
GO:0007584~response to nutrient	2.17	5.01E-03	1.33E-01
GO:0034446~substrate adhesion-dependent cell spreading	10.22	5.12E-03	1.35E-01
GO:0040008~regulation of growth	1.68	5.14E-03	1.34E-01
GO:0006024~glycosaminoglycan biosynthetic process	5.11	5.15E-03	1.33E-01
GO:0008361~regulation of cell size	1.91	5.60E-03	1.43E-01
GO:0051128~regulation of cellular component organization	1.56	5.63E-03	1.42E-01
GO:0009617~response to bacterium	1.95	5.65E-03	1.42E-01
GO:0005882~intermediate filament	1.98	6.03E-03	5.94E-02
GO:0009266~response to temperature stimulus	2.59	6.26E-03	1.55E-01
GO:0016337~cell-cell adhesion	1.75	6.30E-03	1.54E-01
GO:0020037~heme binding	2.26	6.31E-03	1.94E-01
GO:0043542~endothelial cell migration	4.88	6.37E-03	1.55E-01
GO:0042626~ATPase activity, coupled to transmembrane movement of substance	2.35	6.47E-03	1.92E-01
GO:0042625~ATPase activity, coupled to transmembrane movement of ions	2.72	6.79E-03	1.95E-01
GO:0043492~ATPase activity, coupled to movement of substances	2.32	6.98E-03	1.94E-01
GO:0000139~Golgi membrane	1.95	7.15E-03	6.85E-02
GO:0008092~cytoskeletal protein binding	1.52	7.22E-03	1.95E-01
GO:0048468~cell development	1.44	7.23E-03	1.72E-01
GO:0006631~fatty acid metabolic process	1.90	7.46E-03	1.76E-01
GO:0014070~response to organic cyclic substance	2.22	7.50E-03	1.76E-01
GO:0045111~intermediate filament cytoskeleton	1.94	7.57E-03	7.07E-02
GO:0022008~neurogenesis	1.46	7.67E-03	1.78E-01

GO:0005776~autophagic vacuole	6.03	7.71E-03	7.04E-02
GO:0006023~aminoglycan biosynthetic process	4.67	7.77E-03	1.79E-01
GO:0030517~negative regulation of axon extension	8.95	7.85E-03	1.79E-01
GO:0009165~nucleotide biosynthetic process	1.92	7.99E-03	1.81E-01
GO:0022404~molting cycle process	3.41	8.02E-03	1.80E-01
GO:0001942~hair follicle development	3.41	8.02E-03	1.80E-01
GO:0022405~hair cycle process	3.41	8.02E-03	1.80E-01
GO:0048646~anatomical structure formation involved in morphogenesis	1.63	8.03E-03	1.79E-01
GO:0048812~neuron projection morphogenesis	1.85	8.14E-03	1.80E-01
GO:0045926~negative regulation of growth	2.28	8.23E-03	1.80E-01
GO:0051272~positive regulation of cell motion	2.37	8.28E-03	1.80E-01
GO:0040017~positive regulation of locomotion	2.37	8.28E-03	1.80E-01
GO:0004175~endopeptidase activity	1.61	8.49E-03	2.19E-01
GO:0001525~angiogenesis	2.06	8.49E-03	1.83E-01
GO:0032989~cellular component morphogenesis	1.58	8.53E-03	1.83E-01
GO:0030030~cell projection organization	1.60	8.57E-03	1.82E-01
GO:0009201~ribonucleoside triphosphate biosynthetic process	2.35	8.96E-03	1.88E-01
GO:0016043~cellular component organization	1.19	8.99E-03	1.88E-01
GO:0050896~response to stimulus	1.15	9.38E-03	1.94E-01
GO:0008284~positive regulation of cell proliferation	1.56	9.39E-03	1.93E-01
GO:0008233~peptidase activity	1.46	9.64E-03	2.38E-01
GO:0006022~aminoglycan metabolic process	2.75	9.70E-03	1.97E-01
GO:0045087~innate immune response	2.07	1.01E-02	2.04E-01
GO:0032787~monocarboxylic acid metabolic process	1.66	1.02E-02	2.04E-01
GO:0005520~insulin-like growth factor binding	4.38	1.03E-02	2.46E-01
GO:0006636~unsaturated fatty acid biosynthetic process	3.68	1.04E-02	2.07E-01
GO:0021700~developmental maturation	2.30	1.04E-02	2.06E-01
GO:0006754~ATP biosynthetic process	2.41	1.05E-02	2.05E-01
GO:0030335~positive regulation of cell migration	2.41	1.05E-02	2.05E-01
GO:0042834~peptidoglycan binding	8.12	1.07E-02	2.48E-01
GO:0003810~protein-glutamine gamma-glutamyltransferase activity	8.12	1.07E-02	2.48E-01
GO:0030141~secretory granule	1.91	1.07E-02	9.46E-02
GO:0030203~glycosaminoglycan metabolic process	2.93	1.08E-02	2.09E-01
GO:0003824~catalytic activity	1.11	1.10E-02	2.49E-01
GO:0009142~nucleoside triphosphate biosynthetic process	2.28	1.13E-02	2.16E-01
GO:0034404~nucleobase, nucleoside and nucleotide biosynthetic process	1.85	1.16E-02	2.21E-01
GO:0007409~axonogenesis	1.85	1.16E-02	2.21E-01
GO:0034654~nucleobase, nucleoside, nucleotide and nucleic acid biosynthetic pr	1.85	1.16E-02	2.21E-01
GO:0000271~polysaccharide biosynthetic process	3.18	1.17E-02	2.20E-01
GO:0001503~ossification	2.18	1.18E-02	2.20E-01
GO:0048771~tissue remodeling	2.88	1.20E-02	2.23E-01
GO:0007050~cell cycle arrest	2.26	1.21E-02	2.24E-01
GO:0043434~response to peptide hormone stimulus	1.97	1.22E-02	2.24E-01
GO:0005518~collagen binding	3.55	1.25E-02	2.71E-01
GO:0010043~response to zinc ion	5.26	1.28E-02	2.32E-01
GO:0009897~external side of plasma membrane	1.92	1.29E-02	1.11E-01
GO:0051129~negative regulation of cellular component organization	2.02	1.30E-02	2.33E-01
GO:0005764~lysosome	1.80	1.30E-02	1.09E-01
GO:0000323~lytic vacuole	1.80	1.30E-02	1.09E-01
GO:0048660~regulation of smooth muscle cell proliferation	3.11	1.31E-02	2.35E-01
GO:0048667~cell morphogenesis involved in neuron differentiation	1.80	1.31E-02	2.33E-01
GO:0048699~generation of neurons	1.44	1.32E-02	2.33E-01
GO:0045766~positive regulation of angiogenesis	4.13	1.32E-02	2.32E-01

GO:0001944~vasculature development	1.71	1.34E-02	2.33E-01
GO:0007169~transmembrane receptor protein tyrosine kinase signaling pathway	1.76	1.39E-02	2.40E-01
GO:0046034~ATP metabolic process	2.22	1.40E-02	2.40E-01
GO:0031667~response to nutrient levels	1.82	1.42E-02	2.42E-01
GO:0009199~ribonucleoside triphosphate metabolic process	2.12	1.44E-02	2.44E-01
GO:0007517~muscle organ development	1.78	1.45E-02	2.43E-01
GO:0006690~icosanoid metabolic process	3.05	1.47E-02	2.45E-01
GO:0009607~response to biotic stimulus	1.54	1.54E-02	2.54E-01
GO:0007229~integrin-mediated signaling pathway	2.56	1.54E-02	2.53E-01
GO:0031103~axon regeneration	7.16	1.55E-02	2.53E-01
GO:0019898~extrinsic to membrane	1.47	1.55E-02	1.27E-01
GO:0015399~primary active transmembrane transporter activity	2.10	1.60E-02	3.25E-01
GO:0015405~P-P-bond-hydrolysis-driven transmembrane transporter activity	2.10	1.60E-02	3.25E-01
GO:0005254~chloride channel activity	2.54	1.62E-02	3.23E-01
GO:0042598~vesicular fraction	1.71	1.63E-02	1.30E-01
GO:0005626~insoluble fraction	1.34	1.63E-02	1.28E-01
GO:0016787~hydrolase activity	1.18	1.67E-02	3.25E-01
GO:0000041~transition metal ion transport	2.52	1.68E-02	2.70E-01
GO:0050678~regulation of epithelial cell proliferation	2.52	1.68E-02	2.70E-01
GO:0010647~positive regulation of cell communication	1.58	1.73E-02	2.76E-01
GO:0048843~negative regulation of axon extension involved in axon guidance	13.42	1.73E-02	2.74E-01
GO:0048841~regulation of axon extension involved in axon guidance	13.42	1.73E-02	2.74E-01
GO:0009259~ribonucleotide metabolic process	1.95	1.73E-02	2.73E-01
GO:0070011~peptidase activity, acting on L-amino acid peptides	1.43	1.76E-02	3.33E-01
GO:0031982~vesicle	1.38	1.82E-02	1.39E-01
GO:0001568~blood vessel development	1.68	1.89E-02	2.92E-01
GO:0031069~hair follicle morphogenesis	4.71	1.91E-02	2.93E-01
GO:0030246~carbohydrate binding	1.55	1.97E-02	3.57E-01
GO:0060348~bone development	2.04	1.98E-02	3.01E-01
GO:0065008~regulation of biological quality	1.23	1.99E-02	3.02E-01
GO:0017015~regulation of transforming growth factor beta receptor signaling pat	3.21	2.00E-02	3.01E-01
GO:0048518~positive regulation of biological process	1.19	2.00E-02	3.00E-01
GO:0031410~cytoplasmic vesicle	1.38	2.01E-02	1.50E-01
GO:0051707~response to other organism	1.60	2.04E-02	3.04E-01
GO:0019899~enzyme binding	1.43	2.05E-02	3.62E-01
GO:0009206~purine ribonucleoside triphosphate biosynthetic process	2.19	2.05E-02	3.03E-01
GO:0050921~positive regulation of chemotaxis	3.70	2.08E-02	3.06E-01
GO:0060284~regulation of cell development	1.75	2.08E-02	3.04E-01
GO:0009260~ribonucleotide biosynthetic process	2.02	2.10E-02	3.05E-01
GO:0051239~regulation of multicellular organismal process	1.30	2.11E-02	3.06E-01
GO:0052547~regulation of peptidase activity	2.29	2.12E-02	3.05E-01
GO:0010817~regulation of hormone levels	1.90	2.16E-02	3.08E-01
GO:0009991~response to extracellular stimulus	1.71	2.18E-02	3.09E-01
GO:0032403~protein complex binding	1.77	2.18E-02	3.73E-01
GO:0009145~purine nucleoside triphosphate biosynthetic process	2.17	2.20E-02	3.10E-01
GO:0033176~proton-transporting V-type ATPase complex	4.52	2.20E-02	1.60E-01
GO:0009150~purine ribonucleotide metabolic process	1.94	2.20E-02	3.09E-01
GO:0005792~microsome	1.68	2.21E-02	1.58E-01
GO:0042742~defense response to bacterium	2.08	2.23E-02	3.11E-01
GO:0051094~positive regulation of developmental process	1.61	2.25E-02	3.12E-01
GO:0044430~cytoskeletal part	1.29	2.26E-02	1.59E-01
GO:0009968~negative regulation of signal transduction	1.70	2.27E-02	3.13E-01
GO:0032570~response to progesterone stimulus	4.47	2.28E-02	3.13E-01

GO:0045095~keratin filament	2.26	2.29E-02	1.58E-01
GO:0016810~hydrolase activity, acting on carbon-nitrogen (but not peptide) bonc	2.06	2.33E-02	3.87E-01
GO:0006665~sphingolipid metabolic process	2.39	2.33E-02	3.18E-01
GO:0009966~regulation of signal transduction	1.30	2.33E-02	3.16E-01
GO:0005901~caveola	2.78	2.34E-02	1.58E-01
GO:0006955~immune response	1.35	2.35E-02	3.16E-01
GO:0042995~cell projection	1.35	2.37E-02	1.58E-01
GO:0015662~ATPase activity, coupled to transmembrane movement of ions, phos	2.76	2.45E-02	3.96E-01
GO:0015629~actin cytoskeleton	1.61	2.49E-02	1.63E-01
GO:0034329~cell junction assembly	3.05	2.51E-02	3.33E-01
GO:0032103~positive regulation of response to external stimulus	2.52	2.54E-02	3.35E-01
GO:0005253~anion channel activity	2.34	2.61E-02	4.09E-01
GO:0007160~cell-matrix adhesion	2.21	2.63E-02	3.43E-01
GO:0016702~oxidoreductase activity, acting on single donors with incorporation c	2.49	2.69E-02	4.12E-01
GO:0051213~dioxygenase activity	2.49	2.69E-02	4.12E-01
GO:0010466~negative regulation of peptidase activity	4.26	2.70E-02	3.49E-01
GO:0001889~liver development	2.70	2.71E-02	3.49E-01
GO:0048514~blood vessel morphogenesis	1.70	2.71E-02	3.48E-01
GO:0030148~sphingolipid biosynthetic process	3.46	2.72E-02	3.47E-01
GO:0050920~regulation of chemotaxis	3.46	2.72E-02	3.47E-01
GO:0051895~negative regulation of focal adhesion formation	10.73	2.78E-02	3.52E-01
GO:0032695~negative regulation of interleukin-12 production	10.73	2.78E-02	3.52E-01
GO:0003100~regulation of systemic arterial blood pressure by endothelin	10.73	2.78E-02	3.52E-01
GO:0016701~oxidoreductase activity, acting on single donors with incorporation c	2.45	2.91E-02	4.31E-01
GO:0004714~transmembrane receptor protein tyrosine kinase activity	2.45	2.91E-02	4.31E-01
GO:0032535~regulation of cellular component size	1.58	2.99E-02	3.71E-01
GO:0009205~purine ribonucleoside triphosphate metabolic process	1.99	3.01E-02	3.73E-01
GO:0009152~purine ribonucleotide biosynthetic process	1.99	3.01E-02	3.73E-01
GO:0008283~cell proliferation	1.44	3.06E-02	3.76E-01
GO:0048522~positive regulation of cellular process	1.18	3.07E-02	3.75E-01
GO:0051153~regulation of striated muscle cell differentiation	3.35	3.08E-02	3.75E-01
GO:0009141~nucleoside triphosphate metabolic process	1.91	3.13E-02	3.78E-01
GO:0009395~phospholipid catabolic process	4.07	3.16E-02	3.80E-01
GO:0007507~heart development	1.66	3.21E-02	3.83E-01
GO:0048820~hair follicle maturation	5.50	3.26E-02	3.86E-01
GO:0030511~positive regulation of transforming growth factor beta receptor sign	5.50	3.26E-02	3.86E-01
GO:0080134~regulation of response to stress	1.57	3.32E-02	3.91E-01
GO:0048858~cell projection morphogenesis	1.61	3.35E-02	3.92E-01
GO:0009100~glycoprotein metabolic process	1.68	3.38E-02	3.93E-01
GO:0051259~protein oligomerization	1.75	3.41E-02	3.95E-01
GO:0042445~hormone metabolic process	2.03	3.42E-02	3.95E-01
GO:0032870~cellular response to hormone stimulus	1.88	3.48E-02	3.98E-01
GO:0051179~localization	1.13	3.51E-02	4.00E-01
GO:0006643~membrane lipid metabolic process	2.21	3.62E-02	4.08E-01
GO:0016051~carbohydrate biosynthetic process	2.01	3.63E-02	4.08E-01
GO:0002376~immune system process	1.25	3.65E-02	4.08E-01
GO:0007044~cell-substrate junction assembly	3.89	3.66E-02	4.07E-01
GO:0043232~intracellular non-membrane-bounded organelle	1.14	3.69E-02	2.29E-01
GO:0043228~non-membrane-bounded organelle	1.14	3.69E-02	2.29E-01
GO:0006644~phospholipid metabolic process	1.69	3.71E-02	4.10E-01
GO:0006164~purine nucleotide biosynthetic process	1.81	3.72E-02	4.09E-01
GO:0010648~negative regulation of cell communication	1.59	3.76E-02	4.11E-01
GO:0046660~female sex differentiation	2.33	3.77E-02	4.11E-01

GO:0046545~development of primary female sexual characteristics	2.33	3.77E-02	4.11E-01
GO:0016641~oxidoreductase activity, acting on the CH-NH2 group of donors, oxyg	5.22	3.77E-02	5.13E-01
GO:0008329~pattern recognition receptor activity	5.22	3.77E-02	5.13E-01
GO:0030336~negative regulation of cell migration	2.51	3.84E-02	4.16E-01
GO:0050900~leukocyte migration	2.51	3.84E-02	4.16E-01
GO:0046467~membrane lipid biosynthetic process	3.16	3.89E-02	4.18E-01
GO:0048520~positive regulation of behavior	3.16	3.89E-02	4.18E-01
GO:0030728~ovulation	5.11	3.98E-02	4.24E-01
GO:0051604~protein maturation	1.91	3.98E-02	4.23E-01
GO:0009144~purine nucleoside triphosphate metabolic process	1.91	3.98E-02	4.23E-01
GO:0051704~multi-organism process	1.31	4.00E-02	4.23E-01
GO:0019369~arachidonic acid metabolic process	8.95	4.02E-02	4.23E-01
GO:0051797~regulation of hair follicle development	8.95	4.02E-02	4.23E-01
GO:0042634~regulation of hair cycle	8.95	4.02E-02	4.23E-01
GO:0043277~apoptotic cell clearance	8.95	4.02E-02	4.23E-01
GO:0002755~MyD88-dependent toll-like receptor signaling pathway	8.95	4.02E-02	4.23E-01
GO:0008194~UDP-glycosyltransferase activity	1.97	4.03E-02	5.30E-01
GO:0001664~G-protein-coupled receptor binding	1.97	4.03E-02	5.30E-01
GO:0019221~cytokine-mediated signaling pathway	2.30	4.06E-02	4.25E-01
GO:0009967~positive regulation of signal transduction	1.52	4.07E-02	4.25E-01
GO:0016746~transferase activity, transferring acyl groups	1.64	4.11E-02	5.30E-01
GO:0005178~integrin binding	2.48	4.11E-02	5.24E-01
GO:0002683~negative regulation of immune system process	2.16	4.14E-02	4.29E-01
GO:0010810~regulation of cell-substrate adhesion	2.72	4.15E-02	4.29E-01
GO:0016197~endosome transport	2.47	4.17E-02	4.29E-01
GO:0007568~aging	1.95	4.31E-02	4.38E-01
GO:0007565~female pregnancy	1.95	4.31E-02	4.38E-01
GO:0045216~cell-cell junction organization	3.07	4.33E-02	4.39E-01
GO:0005201~extracellular matrix structural constituent	2.12	4.50E-02	5.50E-01
GO:0005355~glucose transmembrane transporter activity	4.87	4.53E-02	5.46E-01
GO:0005976~polysaccharide metabolic process	1.93	4.56E-02	4.54E-01
GO:0005624~membrane fraction	1.28	4.65E-02	2.77E-01
GO:0016298~lipase activity	2.01	4.66E-02	5.50E-01
GO:0005975~carbohydrate metabolic process	1.35	4.67E-02	4.61E-01
GO:0001893~maternal placenta development	4.77	4.77E-02	4.67E-01
GO:0006493~protein amino acid O-linked glycosylation	3.58	4.79E-02	4.67E-01
GO:0050777~negative regulation of immune response	3.58	4.79E-02	4.67E-01
GO:0016485~protein processing	1.92	4.81E-02	4.67E-01
GO:0009887~organ morphogenesis	1.33	4.81E-02	4.66E-01
GO:0009743~response to carbohydrate stimulus	2.39	4.87E-02	4.69E-01
GO:0008415~acyltransferase activity	1.64	4.93E-02	5.65E-01
GO:0004869~cysteine-type endopeptidase inhibitor activity	2.96	4.94E-02	5.60E-01
GO:0042379~chemokine receptor binding	2.61	4.97E-02	5.55E-01
GO:0032990~cell part morphogenesis	1.54	4.97E-02	4.74E-01
GO:0031349~positive regulation of defense response	2.21	5.00E-02	4.75E-01
GO:0051605~protein maturation by peptide bond cleavage	2.08	5.01E-02	4.75E-01
GO:0001968~fibronectin binding	7.83	5.22E-02	5.68E-01
GO:0032868~response to insulin stimulus	1.97	5.22E-02	4.88E-01
GO:0032432~actin filament bundle	3.48	5.24E-02	3.02E-01
GO:0009925~basal plasma membrane	3.48	5.24E-02	3.02E-01
GO:0040013~negative regulation of locomotion	2.35	5.25E-02	4.88E-01
GO:0006821~chloride transport	2.35	5.25E-02	4.88E-01
GO:0005606~laminin-1 complex	7.76	5.31E-02	3.02E-01

GO:0030595~leukocyte chemotaxis	2.90	5.32E-02	4.91E-01
GO:0016747~transferase activity, transferring acyl groups other than amino-acyl g	1.62	5.32E-02	5.69E-01
GO:0005149~interleukin-1 receptor binding	4.57	5.35E-02	5.66E-01
GO:0005247~voltage-gated chloride channel activity	4.57	5.35E-02	5.66E-01
GO:0048511~rhythmic process	1.82	5.40E-02	4.96E-01
GO:0010770~positive regulation of cell morphogenesis involved in differentiation	7.67	5.42E-02	4.96E-01
GO:0051238~sequestering of metal ion	7.67	5.42E-02	4.96E-01
GO:0010718~positive regulation of epithelial to mesenchymal transition	7.67	5.42E-02	4.96E-01
GO:0001501~skeletal system development	1.46	5.43E-02	4.95E-01
GO:0007626~locomotory behavior	1.50	5.49E-02	4.98E-01
GO:0019637~organophosphate metabolic process	1.61	5.55E-02	5.00E-01
GO:0007399~nervous system development	1.22	5.63E-02	5.04E-01
GO:0022612~gland morphogenesis	4.47	5.63E-02	5.03E-01
GO:0050764~regulation of phagocytosis	4.47	5.63E-02	5.03E-01
GO:0031532~actin cytoskeleton reorganization	4.47	5.63E-02	5.03E-01
GO:0043535~regulation of blood vessel endothelial cell migration	4.47	5.63E-02	5.03E-01
GO:0031347~regulation of defense response	1.75	5.66E-02	5.03E-01
GO:0043436~oxoacid metabolic process	1.32	5.66E-02	5.02E-01
GO:0019752~carboxylic acid metabolic process	1.32	5.66E-02	5.02E-01
GO:0010038~response to metal ion	1.80	5.67E-02	5.01E-01
GO:0006163~purine nucleotide metabolic process	1.64	5.67E-02	4.99E-01
GO:0030522~intracellular receptor-mediated signaling pathway	2.15	5.71E-02	5.01E-01
GO:0000302~response to reactive oxygen species	2.15	5.71E-02	5.01E-01
GO:0050840~extracellular matrix binding	3.38	5.72E-02	5.85E-01
GO:0008083~growth factor activity	1.70	5.80E-02	5.84E-01
GO:0008654~phospholipid biosynthetic process	1.93	5.83E-02	5.07E-01
GO:0015718~monocarboxylic acid transport	2.50	5.85E-02	5.07E-01
GO:0009101~glycoprotein biosynthetic process	1.70	5.86E-02	5.06E-01
GO:0042641~actomyosin	3.35	5.89E-02	3.25E-01
GO:0016042~lipid catabolic process	1.65	6.01E-02	5.14E-01
GO:0005938~cell cortex	1.74	6.03E-02	3.28E-01
GO:0001952~regulation of cell-matrix adhesion	3.31	6.09E-02	5.18E-01
GO:0043005~neuron projection	1.43	6.17E-02	3.30E-01
GO:0009117~nucleotide metabolic process	1.47	6.18E-02	5.22E-01
GO:0006753~nucleoside phosphate metabolic process	1.47	6.18E-02	5.22E-01
GO:0006869~lipid transport	1.73	6.18E-02	5.21E-01
GO:0008219~cell death	1.27	6.27E-02	5.24E-01
GO:0048634~regulation of muscle development	2.46	6.33E-02	5.27E-01
GO:0006082~organic acid metabolic process	1.31	6.34E-02	5.26E-01
GO:0016706~oxidoreductase activity, acting on paired donors, with incorporation	3.26	6.40E-02	6.16E-01
GO:0051147~regulation of muscle cell differentiation	2.75	6.42E-02	5.29E-01
GO:0060326~cell chemotaxis	2.75	6.42E-02	5.29E-01
GO:0016023~cytoplasmic membrane-bounded vesicle	1.32	6.45E-02	3.38E-01
GO:0008585~female gonad development	2.24	6.49E-02	5.32E-01
GO:0051235~maintenance of location	2.24	6.49E-02	5.32E-01
GO:0045471~response to ethanol	2.24	6.49E-02	5.32E-01
GO:0002221~pattern recognition receptor signaling pathway	4.21	6.57E-02	5.35E-01
GO:0042177~negative regulation of protein catabolic process	4.21	6.57E-02	5.35E-01
GO:0035295~tube development	1.55	6.71E-02	5.41E-01
GO:0008253~5'-nucleotidase activity	6.85	6.71E-02	6.29E-01
GO:0004029~aldehyde dehydrogenase (NAD) activity	6.85	6.71E-02	6.29E-01
GO:0016812~hydrolase activity, acting on carbon-nitrogen (but not peptide) bonc	6.85	6.71E-02	6.29E-01
GO:0022607~cellular component assembly	1.23	6.79E-02	5.45E-01

GO:0010811~positive regulation of cell-substrate adhesion	3.19	6.80E-02	5.44E-01
GO:0016055~Wnt receptor signaling pathway	1.75	6.82E-02	5.44E-01
GO:0045664~regulation of neuron differentiation	1.75	6.82E-02	5.44E-01
GO:0016265~death	1.26	6.82E-02	5.43E-01
GO:0005768~endosome	1.44	6.86E-02	3.52E-01
GO:0051099~positive regulation of binding	2.06	6.88E-02	5.44E-01
GO:0016757~transferase activity, transferring glycosyl groups	1.50	6.88E-02	6.33E-01
GO:0035313~wound healing, spreading of epidermal cells	6.71	6.96E-02	5.48E-01
GO:0030857~negative regulation of epithelial cell differentiation	6.71	6.96E-02	5.48E-01
GO:0055093~response to hyperoxia	6.71	6.96E-02	5.48E-01
GO:0008347~glial cell migration	6.71	6.96E-02	5.48E-01
GO:0051893~regulation of focal adhesion formation	6.71	6.96E-02	5.48E-01
GO:0007016~cytoskeletal anchoring at plasma membrane	6.71	6.96E-02	5.48E-01
GO:0048675~axon extension	6.71	6.96E-02	5.48E-01
GO:0006527~arginine catabolic process	6.71	6.96E-02	5.48E-01
GO:0031670~cellular response to nutrient	6.71	6.96E-02	5.48E-01
GO:0007033~vacuole organization	2.68	7.02E-02	5.49E-01
GO:0043292~contractile fiber	1.79	7.08E-02	3.57E-01
GO:0030674~protein binding, bridging	1.94	7.13E-02	6.41E-01
GO:0016638~oxidoreductase activity, acting on the CH-NH2 group of donors	4.06	7.20E-02	6.39E-01
GO:0004653~polypeptide N-acetylgalactosaminyltransferase activity	4.06	7.20E-02	6.39E-01
GO:0015149~hexose transmembrane transporter activity	4.06	7.20E-02	6.39E-01
GO:0006633~fatty acid biosynthetic process	2.04	7.30E-02	5.63E-01
GO:0033178~proton-transporting two-sector ATPase complex, catalytic domain	4.02	7.37E-02	3.65E-01
GO:0042180~cellular ketone metabolic process	1.29	7.53E-02	5.73E-01
GO:0043122~regulation of I-kappaB kinase/NF-kappaB cascade	1.84	7.54E-02	5.73E-01
GO:0043588~skin development	3.08	7.56E-02	5.72E-01
GO:0030593~neutrophil chemotaxis	3.98	7.57E-02	5.71E-01
GO:0007492~endoderm development	3.98	7.57E-02	5.71E-01
GO:0033993~response to lipid	3.98	7.57E-02	5.71E-01
GO:0045987~positive regulation of smooth muscle contraction	3.98	7.57E-02	5.71E-01
GO:0042551~neuron maturation	3.98	7.57E-02	5.71E-01
GO:0051092~positive regulation of NF-kappaB transcription factor activity	2.62	7.64E-02	5.74E-01
GO:0015144~carbohydrate transmembrane transporter activity	3.04	7.87E-02	6.68E-01
GO:0042476~odontogenesis	2.32	7.92E-02	5.86E-01
GO:0008360~regulation of cell shape	2.32	7.92E-02	5.86E-01
GO:0031252~cell leading edge	1.70	7.94E-02	3.84E-01
GO:0045178~basal part of cell	3.02	8.09E-02	3.85E-01
GO:0005507~copper ion binding	2.12	8.21E-02	6.79E-01
GO:0015145~monosaccharide transmembrane transporter activity	3.85	8.22E-02	6.74E-01
GO:0016755~transferase activity, transferring amino-acyl groups	3.85	8.22E-02	6.74E-01
GO:0046483~heterocycle metabolic process	1.39	8.26E-02	6.01E-01
GO:0050780~dopamine receptor binding	6.09	8.33E-02	6.74E-01
GO:0001786~phosphatidylserine binding	6.09	8.33E-02	6.74E-01
GO:0008252~nucleotidase activity	6.09	8.33E-02	6.74E-01
GO:0034614~cellular response to reactive oxygen species	2.98	8.36E-02	6.04E-01
GO:0007566~embryo implantation	2.98	8.36E-02	6.04E-01
GO:0010740~positive regulation of protein kinase cascade	1.61	8.39E-02	6.05E-01
GO:0005829~cytosol	1.17	8.49E-02	3.96E-01
GO:0051048~negative regulation of secretion	2.28	8.49E-02	6.08E-01
GO:0002218~activation of innate immune response	3.77	8.63E-02	6.13E-01
GO:0006693~prostaglandin metabolic process	3.77	8.63E-02	6.13E-01
GO:0002758~innate immune response-activating signal transduction	3.77	8.63E-02	6.13E-01

GO:0006692~prostanoid metabolic process	3.77	8.63E-02	6.13E-01
GO:0048008~platelet-derived growth factor receptor signaling pathway	3.77	8.63E-02	6.13E-01
GO:0000038~very-long-chain fatty acid metabolic process	3.77	8.63E-02	6.13E-01
GO:0034405~response to fluid shear stress	5.96	8.63E-02	6.12E-01
GO:0043536~positive regulation of blood vessel endothelial cell migration	5.96	8.63E-02	6.12E-01
GO:0031638~zymogen activation	5.96	8.63E-02	6.12E-01
GO:0048009~insulin-like growth factor receptor signaling pathway	5.96	8.63E-02	6.12E-01
GO:0019432~triglyceride biosynthetic process	5.96	8.63E-02	6.12E-01
GO:0043537~negative regulation of blood vessel endothelial cell migration	5.96	8.63E-02	6.12E-01
GO:0010717~regulation of epithelial to mesenchymal transition	5.96	8.63E-02	6.12E-01
GO:0016045~detection of bacterium	5.96	8.63E-02	6.12E-01
GO:0001738~morphogenesis of a polarized epithelium	5.96	8.63E-02	6.12E-01
GO:0030173~integral to Golgi membrane	2.53	8.65E-02	3.98E-01
GO:0008376~acetylgalactosaminyltransferase activity	2.95	8.67E-02	6.85E-01
GO:0009308~amine metabolic process	1.34	8.87E-02	6.21E-01
GO:0034599~cellular response to oxidative stress	2.50	8.98E-02	6.25E-01
GO:0007423~sensory organ development	1.48	9.01E-02	6.25E-01
GO:0009408~response to heat	2.24	9.08E-02	6.27E-01
GO:0030017~sarcomere	1.85	9.14E-02	4.12E-01
GO:0046456~icosanoid biosynthetic process	2.89	9.19E-02	6.30E-01
GO:0048661~positive regulation of smooth muscle cell proliferation	2.89	9.19E-02	6.30E-01
GO:0051187~cofactor catabolic process	2.89	9.19E-02	6.30E-01
GO:0031988~membrane-bounded vesicle	1.27	9.29E-02	4.13E-01
GO:0046394~carboxylic acid biosynthetic process	1.62	9.31E-02	6.34E-01
GO:0016053~organic acid biosynthetic process	1.62	9.31E-02	6.34E-01
GO:0048608~reproductive structure development	1.70	9.38E-02	6.36E-01
GO:0000267~cell fraction	1.19	9.40E-02	4.13E-01
GO:0044449~contractile fiber part	1.76	9.44E-02	4.10E-01
GO:0043388~positive regulation of DNA binding	2.04	9.47E-02	6.38E-01
GO:0045637~regulation of myeloid cell differentiation	2.04	9.47E-02	6.38E-01
GO:0016903~oxidoreductase activity, acting on the aldehyde or oxo group of don	2.85	9.50E-02	7.15E-01
GO:0004601~peroxidase activity	2.85	9.50E-02	7.15E-01
GO:0016684~oxidoreductase activity, acting on peroxide as acceptor	2.85	9.50E-02	7.15E-01
GO:0031406~carboxylic acid binding	1.65	9.59E-02	7.14E-01
GO:0016811~hydrolase activity, acting on carbon-nitrogen (but not peptide) bonc	2.20	9.60E-02	7.09E-01
GO:0031669~cellular response to nutrient levels	2.44	9.69E-02	6.46E-01
GO:0044057~regulation of system process	1.39	9.71E-02	6.45E-01
GO:0022804~active transmembrane transporter activity	1.36	9.72E-02	7.09E-01
GO:0002685~regulation of leukocyte migration	3.58	9.75E-02	6.46E-01
GO:0050830~defense response to Gram-positive bacterium	3.58	9.75E-02	6.46E-01
GO:0006805~xenobiotic metabolic process	3.58	9.75E-02	6.46E-01
GO:0048678~response to axon injury	3.58	9.75E-02	6.46E-01
GO:0050660~FAD binding	2.03	9.78E-02	7.07E-01
GO:0045137~development of primary sexual characteristics	1.69	9.78E-02	6.46E-01
GO:0009055~electron carrier activity	1.49	9.82E-02	7.04E-01
