# Supplementary material for: Plasticity of Airway Epithelial Cell Transcriptome in Response to Flagellin
Source: PLoS One. 2015 Feb 10;10(2):e0115486. doi: 10.1371/journal.pone.0115486 (PMC4323341; doi:10.1371/journal.pone.0115486)
Supplement: S3 Table — (PDF) [file pone.0115486.s004.pdf]

Supplementary Table 3. Complete results from functional enrichment analysis of differentially expressed genes following exposure to flagellin in monolayer AEC cultures as identified by exon microarrays.

Gene Ontology Annotation	Fold Enrichment	P-value	Adjusted P-value
GO:0002376~immune system process	3.75	1.21E-29	3.05E-26
GO:0006955~immune response	4.20	1.79E-25	2.25E-22
GO:0006952~defense response	4.32	6.09E-24	5.12E-21
GO:0005125~cytokine activity	7.79	3.68E-21	2.29E-18
GO:0005615~extracellular space	3.74	1.64E-18	4.74E-16
GO:0006954~inflammatory response	5.20	4.86E-18	3.06E-15
GO:0044421~extracellular region part	3.11	2.28E-17	3.29E-15
GO:0051707~response to other organism	5.41	2.39E-17	1.20E-14
GO:0009611~response to wounding	3.95	4.04E-17	1.70E-14
GO:0009605~response to external stimulus	2.99	4.87E-16	1.60E-13
GO:0006950~response to stress	2.32	1.24E-15	3.85E-13
GO:0051704~multi-organism process	3.31	5.78E-15	1.62E-12
GO:0009607~response to biotic stimulus	4.29	1.16E-14	2.91E-12
GO:0050896~response to stimulus	1.75	2.49E-14	5.72E-12
GO:0005576~extracellular region	2.14	5.83E-14	5.61E-12
GO:0005102~receptor binding	2.90	1.03E-13	3.22E-11
GO:0008009~chemokine activity	13.75	1.60E-12	3.32E-10
GO:0009617~response to bacterium	5.63	1.88E-12	3.95E-10
GO:0042379~chemokine receptor binding	12.91	4.22E-12	6.57E-10
GO:0042221~response to chemical stimulus	2.32	8.03E-12	1.56E-09
GO:0006935~chemotaxis	5.53	4.39E-10	7.90E-08
GO:0042330~taxis	5.53	4.39E-10	7.90E-08
GO:0001817~regulation of cytokine production	5.11	7.48E-10	1.26E-07
GO:0001664~G-protein-coupled receptor binding	6.46	7.23E-09	9.00E-07
GO:0006915~apoptosis	2.74	1.28E-08	2.02E-06
GO:0012501~programmed cell death	2.70	1.94E-08	2.88E-06
GO:0002237~response to molecule of bacterial origin	7.01	2.31E-08	3.24E-06
GO:0002682~regulation of immune system process	3.24	2.95E-08	3.92E-06
GO:0051239~regulation of multicellular organismal process	2.27	3.28E-08	4.14E-06
GO:0042981~regulation of apoptosis	2.40	3.46E-08	4.15E-06
GO:0043067~regulation of programmed cell death	2.38	4.65E-08	5.32E-06
GO:0001775~cell activation	3.64	5.18E-08	5.67E-06
GO:0010941~regulation of cell death	2.37	5.23E-08	5.50E-06
GO:0045087~innate immune response	5.25	5.41E-08	5.45E-06
GO:0010646~regulation of cell communication	2.17	6.00E-08	5.82E-06
GO:0008219~cell death	2.46	7.39E-08	6.90E-06
GO:0016265~death	2.44	9.03E-08	8.13E-06
GO:0031347~regulation of defense response	5.06	9.18E-08	7.98E-06
GO:0048518~positive regulation of biological process	1.74	1.19E-07	1.00E-05
GO:0048583~regulation of response to stimulus	2.85	1.83E-07	1.48E-05
GO:0007610~behavior	2.83	2.23E-07	1.75E-05
GO:0007626~locomotory behavior	3.52	3.48E-07	2.66E-05
GO:0032496~response to lipopolysaccharide	6.79	3.86E-07	2.86E-05
GO:0002684~positive regulation of immune system process	3.72	5.03E-07	3.62E-05
GO:0034097~response to cytokine stimulus	6.62	5.15E-07	3.60E-05
GO:0010033~response to organic substance	2.34	5.85E-07	3.98E-05
GO:0045321~leukocyte activation	3.66	6.61E-07	4.38E-05
GO:0002526~acute inflammatory response	5.75	8.61E-07	5.57E-05
GO:0050776~regulation of immune response	3.74	8.99E-07	5.66E-05
GO:0040011~locomotion	2.80	1.11E-06	6.80E-05

GO:0080134~regulation of response to stress	3.38	1.33E-06	7.99E-05
GO:0001819~positive regulation of cytokine production	5.81	2.16E-06	1.26E-04
GO:0048522~positive regulation of cellular process	1.70	2.22E-06	1.27E-04
GO:0030595~leukocyte chemotaxis	9.78	2.72E-06	1.52E-04
GO:0009966~regulation of signal transduction	2.11	2.73E-06	1.49E-04
GO:0009615~response to virus	5.17	2.93E-06	1.57E-04
GO:0005515~protein binding	1.22	3.05E-06	3.16E-04
GO:0042742~defense response to bacterium	5.03	3.99E-06	2.09E-04
GO:0060326~cell chemotaxis	9.28	4.14E-06	2.13E-04
GO:0006953~acute-phase response	9.05	5.07E-06	2.55E-04
GO:0051094~positive regulation of developmental process	3.18	6.07E-06	3.00E-04
GO:0048513~organ development	1.69	6.54E-06	3.17E-04
GO:0048584~positive regulation of response to stimulus	3.41	6.81E-06	3.24E-04
GO:0050900~leukocyte migration	7.06	1.01E-05	4.69E-04
GO:0070482~response to oxygen levels	4.28	1.08E-05	4.93E-04
GO:0051240~positive regulation of multicellular organismal process	3.30	1.10E-05	4.93E-04
GO:0031349~positive regulation of defense response	6.06	1.20E-05	5.29E-04
GO:0008329~pattern recognition receptor activity	18.08	1.21E-05	1.07E-03
GO:0006916~anti-apoptosis	3.51	1.51E-05	6.54E-04
GO:0002520~immune system development	3.06	1.86E-05	7.95E-04
GO:0048731~system development	1.54	1.96E-05	8.25E-04
GO:0032879~regulation of localization	2.24	2.24E-05	9.23E-04
GO:0030097~hemopoiesis	3.24	2.47E-05	1.00E-03
GO:0005149~interleukin-1 receptor binding	15.82	2.53E-05	1.97E-03
GO:0048534~hemopoietic or lymphoid organ development	3.09	2.68E-05	1.07E-03
GO:0042127~regulation of cell proliferation	2.04	2.83E-05	1.11E-03
GO:0001666~response to hypoxia	4.20	2.84E-05	1.10E-03
GO:0050793~regulation of developmental process	2.15	2.92E-05	1.11E-03
GO:0048856~anatomical structure development	1.50	2.95E-05	1.11E-03
GO:0030234~enzyme regulator activity	2.01	4.20E-05	2.90E-03
GO:0004904~interferon receptor activity	42.18	5.15E-05	3.20E-03
GO:0019961~interferon binding	42.18	5.15E-05	3.20E-03
GO:0019221~cytokine-mediated signaling pathway	5.75	5.48E-05	2.03E-03
GO:0030593~neutrophil chemotaxis	13.41	5.98E-05	2.18E-03
GO:0070665~positive regulation of leukocyte proliferation	6.46	6.63E-05	2.38E-03
GO:0032946~positive regulation of mononuclear cell proliferation	6.46	6.63E-05	2.38E-03
GO:0065008~regulation of biological quality	1.67	6.80E-05	2.41E-03
GO:0042110~T cell activation	4.15	6.90E-05	2.41E-03
GO:0043066~negative regulation of apoptosis	2.61	7.54E-05	2.60E-03
GO:0048869~cellular developmental process	1.60	7.95E-05	2.70E-03
GO:0043069~negative regulation of programmed cell death	2.58	9.21E-05	3.09E-03
GO:0060548~negative regulation of cell death	2.57	9.59E-05	3.18E-03
GO:0030099~myeloid cell differentiation	4.76	1.00E-04	3.28E-03
GO:0002521~leukocyte differentiation	3.99	1.01E-04	3.25E-03
GO:0030574~collagen catabolic process	12.06	1.04E-04	3.31E-03
GO:0050727~regulation of inflammatory response	5.29	1.05E-04	3.31E-03
GO:0051384~response to glucocorticoid stimulus	5.16	1.29E-04	4.01E-03
GO:0046649~lymphocyte activation	3.23	1.33E-04	4.08E-03
GO:0060341~regulation of cellular localization	2.92	1.54E-04	4.68E-03
GO:0048519~negative regulation of biological process	1.55	1.55E-04	4.64E-03
GO:0040017~positive regulation of locomotion	4.51	1.56E-04	4.62E-03
GO:0051272~positive regulation of cell motion	4.51	1.56E-04	4.62E-03
GO:0032101~regulation of response to external stimulus	3.54	1.66E-04	4.86E-03
GO:0032103~positive regulation of response to external stimulus	5.66	1.74E-04	5.03E-03

GO:0002697~regulation of immune effector process	4.38	2.01E-04	5.73E-03
GO:0070663~regulation of leukocyte proliferation	4.79	2.29E-04	6.46E-03
GO:0032944~regulation of mononuclear cell proliferation	4.79	2.29E-04	6.46E-03
GO:0030154~cell differentiation	1.57	2.42E-04	6.74E-03
GO:0065007~biological regulation	1.18	2.47E-04	6.81E-03
GO:0031960~response to corticosteroid stimulus	4.73	2.51E-04	6.84E-03
GO:0032502~developmental process	1.37	2.75E-04	7.42E-03
GO:0030334~regulation of cell migration	3.33	3.03E-04	8.10E-03
GO:0002224~toll-like receptor signaling pathway	14.36	3.05E-04	8.07E-03
GO:0040012~regulation of locomotion	3.14	3.12E-04	8.16E-03
GO:0048545~response to steroid hormone stimulus	3.14	3.12E-04	8.16E-03
GO:0051249~regulation of lymphocyte activation	3.53	3.18E-04	8.23E-03
GO:0051270~regulation of cell motion	3.13	3.29E-04	8.43E-03
GO:0030335~positive regulation of cell migration	4.52	3.55E-04	8.99E-03
GO:0042102~positive regulation of T cell proliferation	7.22	3.64E-04	9.13E-03
GO:0044243~multicellular organismal catabolic process	9.28	3.90E-04	9.68E-03
GO:0050671~positive regulation of lymphocyte proliferation	5.85	3.96E-04	9.73E-03
GO:0008083~growth factor activity	3.41	4.50E-04	2.51E-02
GO:0042035~regulation of cytokine biosynthetic process	4.89	4.78E-04	1.16E-02
GO:0051092~positive regulation of NF-kappaB transcription factor activity	6.87	4.81E-04	1.16E-02
GO:0032494~response to peptidoglycan	22.98	4.91E-04	1.17E-02
GO:0048247~lymphocyte chemotaxis	22.98	4.91E-04	1.17E-02
GO:0009719~response to endogenous stimulus	2.28	5.00E-04	1.18E-02
GO:0051046~regulation of secretion	2.99	5.21E-04	1.22E-02
GO:0051223~regulation of protein transport	3.88	5.35E-04	1.24E-02
GO:0016477~cell migration	2.62	5.40E-04	1.24E-02
GO:0051049~regulation of transport	2.22	5.50E-04	1.25E-02
GO:0032963~collagen metabolic process	8.62	5.59E-04	1.26E-02
GO:0045595~regulation of cell differentiation	2.13	5.69E-04	1.27E-02
GO:0050870~positive regulation of T cell activation	4.76	5.73E-04	1.27E-02
GO:0010647~positive regulation of cell communication	2.44	5.78E-04	1.27E-02
GO:0002250~adaptive immune response	4.70	6.26E-04	1.36E-02
GO:0002460~adaptive immune response based on somatic recombination of immu	4.70	6.26E-04	1.36E-02
GO:0004866~endopeptidase inhibitor activity	3.49	6.65E-04	3.39E-02
GO:0048870~cell motility	2.49	6.68E-04	1.44E-02
GO:0051674~localization of cell	2.49	6.68E-04	1.44E-02
GO:0051251~positive regulation of lymphocyte activation	4.15	6.71E-04	1.43E-02
GO:0002221~pattern recognition receptor signaling pathway	11.83	6.85E-04	1.45E-02
GO:0032655~regulation of interleukin-12 production	11.83	6.85E-04	1.45E-02
GO:0008015~blood circulation	3.03	7.57E-04	1.59E-02
GO:0003013~circulatory system process	3.03	7.57E-04	1.59E-02
GO:0050729~positive regulation of inflammatory response	8.04	7.79E-04	1.62E-02
GO:0004222~metalloendopeptidase activity	4.06	7.96E-04	3.74E-02
GO:0006928~cell motion	2.12	8.01E-04	1.66E-02
GO:0007165~signal transduction	1.36	8.25E-04	1.69E-02
GO:0042129~regulation of T cell proliferation	5.19	8.27E-04	1.68E-02
GO:0048872~homeostasis of number of cells	4.02	8.37E-04	1.69E-02
GO:0070201~regulation of establishment of protein localization	3.66	8.54E-04	1.71E-02
GO:0009725~response to hormone stimulus	2.30	8.58E-04	1.70E-02
GO:0002694~regulation of leukocyte activation	3.15	8.92E-04	1.76E-02
GO:0044259~multicellular organismal macromolecule metabolic process	7.78	9.10E-04	1.78E-02
GO:0010740~positive regulation of protein kinase cascade	3.13	9.40E-04	1.82E-02
GO:0050778~positive regulation of immune response	3.33	9.76E-04	1.88E-02
GO:0042834~peptidoglycan binding	18.75	9.90E-04	4.31E-02

GO:0042592~homeostatic process	1.82	1.02E-03	1.95E-02
GO:0050670~regulation of lymphocyte proliferation	4.36	1.03E-03	1.95E-02
GO:0050794~regulation of cellular process	1.17	1.03E-03	1.94E-02
GO:0030414~peptidase inhibitor activity	3.31	1.04E-03	4.21E-02
GO:0002758~innate immune response-activating signal transduction	10.58	1.07E-03	2.00E-02
GO:0002218~activation of innate immune response	10.58	1.07E-03	2.00E-02
GO:0044060~regulation of endocrine process	17.87	1.14E-03	2.10E-02
GO:0009888~tissue development	1.87	1.16E-03	2.12E-02
GO:0002696~positive regulation of leukocyte activation	3.79	1.27E-03	2.31E-02
GO:0050830~defense response to Gram-positive bacterium	10.05	1.31E-03	2.37E-02
GO:0051050~positive regulation of transport	2.71	1.37E-03	2.45E-02
GO:0002573~myeloid leukocyte differentiation	7.10	1.40E-03	2.49E-02
GO:0050865~regulation of cell activation	2.99	1.41E-03	2.49E-02
GO:0007166~cell surface receptor linked signal transduction	1.45	1.42E-03	2.49E-02
GO:0004867~serine-type endopeptidase inhibitor activity	4.13	1.49E-03	5.65E-02
GO:0050789~regulation of biological process	1.16	1.53E-03	2.65E-02
GO:0001533~cornified envelope	9.69	1.54E-03	1.05E-01
GO:0008284~positive regulation of cell proliferation	2.14	1.59E-03	2.75E-02
GO:0007565~female pregnancy	3.66	1.65E-03	2.82E-02
GO:0007275~multicellular organismal development	1.33	1.67E-03	2.85E-02
GO:0045597~positive regulation of cell differentiation	2.63	1.75E-03	2.96E-02
GO:0050867~positive regulation of cell activation	3.62	1.75E-03	2.94E-02
GO:0005539~glycosaminoglycan binding	3.31	1.82E-03	6.45E-02
GO:0004896~cytokine receptor activity	5.37	1.83E-03	6.13E-02
GO:0044236~multicellular organismal metabolic process	6.52	2.07E-03	3.44E-02
GO:0045088~regulation of innate immune response	5.21	2.11E-03	3.48E-02
GO:0030155~regulation of cell adhesion	3.23	2.19E-03	3.59E-02
GO:0030855~epithelial cell differentiation	3.23	2.19E-03	3.59E-02
GO:0002253~activation of immune response	3.85	2.30E-03	3.75E-02
GO:0051174~regulation of phosphorus metabolic process	1.99	2.31E-03	3.73E-02
GO:0019220~regulation of phosphate metabolic process	1.99	2.31E-03	3.73E-02
GO:0032880~regulation of protein localization	3.21	2.31E-03	3.71E-02
GO:0043065~positive regulation of apoptosis	2.06	2.49E-03	3.98E-02
GO:0050863~regulation of T cell activation	3.44	2.52E-03	3.99E-02
GO:0034364~high-density lipoprotein particle	8.53	2.52E-03	1.36E-01
GO:0002764~immune response-regulating signal transduction	5.03	2.54E-03	4.01E-02
GO:0048523~negative regulation of cellular process	1.45	2.66E-03	4.16E-02
GO:0043068~positive regulation of programmed cell death	2.04	2.71E-03	4.20E-02
GO:0010942~positive regulation of cell death	2.03	2.88E-03	4.44E-02
GO:0009967~positive regulation of signal transduction	2.32	2.89E-03	4.43E-02
GO:0050708~regulation of protein secretion	4.85	3.04E-03	4.63E-02
GO:0033256~I-kappaB/NF-kappaB complex	31.99	3.17E-03	1.42E-01
GO:0008745~N-acetylmuramoyl-L-alanine amidase activity	31.64	3.24E-03	1.01E-01
GO:0008217~regulation of blood pressure	3.62	3.39E-03	5.11E-02
GO:0006959~humoral immune response	4.07	3.41E-03	5.11E-02
GO:0045785~positive regulation of cell adhesion	4.69	3.61E-03	5.38E-02
GO:0051091~positive regulation of transcription factor activity	4.69	3.61E-03	5.38E-02
GO:0010212~response to ionizing radiation	4.69	3.61E-03	5.38E-02
GO:0004175~endopeptidase activity	2.14	3.64E-03	1.07E-01
GO:0001871~pattern binding	3.01	3.65E-03	1.03E-01
GO:0030247~polysaccharide binding	3.01	3.65E-03	1.03E-01
GO:0005578~proteinaceous extracellular matrix	2.27	3.67E-03	1.41E-01
GO:0010627~regulation of protein kinase cascade	2.42	3.76E-03	5.56E-02
GO:0031424~keratinization	5.61	4.05E-03	5.93E-02

GO:0051090~regulation of transcription factor activity	3.51	4.06E-03	5.92E-02
GO:0008237~metallopeptidase activity	2.77	4.27E-03	1.14E-01
GO:0060429~epithelium development	2.48	4.42E-03	6.39E-02
GO:0051098~regulation of binding	2.89	4.84E-03	6.94E-02
GO:0008233~peptidase activity	1.84	5.04E-03	1.28E-01
GO:0043122~regulation of I-kappaB kinase/NF-kappaB cascade	3.38	5.11E-03	7.28E-02
GO:0007243~protein kinase cascade	2.07	5.13E-03	7.26E-02
GO:0004857~enzyme inhibitor activity	2.34	5.18E-03	1.26E-01
GO:0007167~enzyme linked receptor protein signaling pathway	2.12	5.23E-03	7.36E-02
GO:0016019~peptidoglycan receptor activity	25.31	5.32E-03	1.24E-01
GO:0030217~T cell differentiation	4.33	5.38E-03	7.51E-02
GO:0002274~myeloid leukocyte activation	5.25	5.42E-03	7.53E-02
GO:0045089~positive regulation of innate immune response	5.25	5.42E-03	7.53E-02
GO:0001816~cytokine production	5.25	5.42E-03	7.53E-02
GO:0042402~biogenic amine catabolic process	10.72	5.51E-03	7.61E-02
GO:0051047~positive regulation of secretion	3.32	5.71E-03	7.84E-02
GO:0030216~keratinocyte differentiation	4.27	5.79E-03	7.90E-02
GO:0070011~peptidase activity, acting on L-amino acid peptides	1.84	5.84E-03	1.31E-01
GO:0050707~regulation of cytokine secretion	6.70	6.12E-03	8.28E-02
GO:0032663~regulation of interleukin-2 production	6.70	6.12E-03	8.28E-02
GO:0008544~epidermis development	2.62	6.23E-03	8.38E-02
GO:0051222~positive regulation of protein transport	4.20	6.23E-03	8.34E-02
GO:0042325~regulation of phosphorylation	1.90	6.26E-03	8.33E-02
GO:0045121~membrane raft	2.98	6.50E-03	2.10E-01
GO:0042108~positive regulation of cytokine biosynthetic process	5.03	6.51E-03	8.60E-02
GO:0032501~multicellular organismal process	1.21	6.51E-03	8.56E-02
GO:0033280~response to vitamin D	10.05	6.66E-03	8.70E-02
GO:0031325~positive regulation of cellular metabolic process	1.60	6.76E-03	8.77E-02
GO:0008637~apoptotic mitochondrial changes	6.49	6.89E-03	8.89E-02
GO:0005625~soluble fraction	2.18	7.07E-03	2.04E-01
GO:0051716~cellular response to stimulus	1.62	7.43E-03	9.51E-02
GO:0051052~regulation of DNA metabolic process	3.17	7.45E-03	9.49E-02
GO:0031012~extracellular matrix	2.10	7.45E-03	1.94E-01
GO:0043388~positive regulation of DNA binding	4.02	7.70E-03	9.74E-02
GO:0051241~negative regulation of multicellular organismal process	2.70	7.80E-03	9.82E-02
GO:0007267~cell-cell signaling	1.74	7.96E-03	9.96E-02
GO:0042060~wound healing	2.53	8.17E-03	1.02E-01
GO:0022414~reproductive process	1.64	8.56E-03	1.06E-01
GO:0002755~MyD88-dependent toll-like receptor signaling pathway	20.11	8.61E-03	1.06E-01
GO:0034358~plasma lipoprotein particle	6.09	8.69E-03	2.05E-01
GO:0032994~protein-lipid complex	6.09	8.69E-03	2.05E-01
GO:0009913~epidermal cell differentiation	3.91	8.81E-03	1.08E-01
GO:0000003~reproduction	1.63	9.11E-03	1.10E-01
GO:0002757~immune response-activating signal transduction	4.64	9.12E-03	1.10E-01
GO:0065009~regulation of molecular function	1.54	9.80E-03	1.17E-01
GO:0043123~positive regulation of I-kappaB kinase/NF-kappaB cascade	3.32	1.04E-02	1.23E-01
GO:0051101~regulation of DNA binding	2.99	1.05E-02	1.24E-01
GO:0005138~interleukin-6 receptor binding	18.08	1.08E-02	2.22E-01
GO:0032755~positive regulation of interleukin-6 production	8.47	1.09E-02	1.28E-01
GO:0042219~cellular amino acid derivative catabolic process	8.47	1.09E-02	1.28E-01
GO:0007398~ectoderm development	2.43	1.09E-02	1.27E-01
GO:0001525~angiogenesis	2.72	1.15E-02	1.33E-01
GO:0043407~negative regulation of MAP kinase activity	5.59	1.17E-02	1.35E-01
GO:0032675~regulation of interleukin-6 production	5.59	1.17E-02	1.35E-01

GO:0009253~peptidoglycan catabolic process	17.24	1.19E-02	1.36E-01
GO:0000270~peptidoglycan metabolic process	17.24	1.19E-02	1.36E-01
GO:0009893~positive regulation of metabolic process	1.53	1.28E-02	1.45E-01
GO:0051099~positive regulation of binding	3.61	1.28E-02	1.45E-01
GO:0007596~blood coagulation	3.15	1.34E-02	1.50E-01
GO:0050817~coagulation	3.15	1.34E-02	1.50E-01
GO:0006917~induction of apoptosis	2.01	1.40E-02	1.56E-01
GO:0030098~lymphocyte differentiation	3.12	1.41E-02	1.56E-01
GO:0051701~interaction with host	5.29	1.41E-02	1.55E-01
GO:0012502~induction of programmed cell death	2.00	1.43E-02	1.57E-01
GO:0045429~positive regulation of nitric oxide biosynthetic process	7.66	1.44E-02	1.57E-01
GO:0001836~release of cytochrome c from mitochondria	7.66	1.44E-02	1.57E-01
GO:0031093~platelet alpha granule lumen	5.20	1.51E-02	3.06E-01
GO:0043627~response to estrogen stimulus	3.06	1.56E-02	1.68E-01
GO:0048514~blood vessel morphogenesis	2.29	1.64E-02	1.75E-01
GO:0008633~activation of pro-apoptotic gene products	7.31	1.64E-02	1.75E-01
GO:0051789~response to protein stimulus	3.01	1.71E-02	1.81E-01
GO:0042802~identical protein binding	1.65	1.78E-02	3.28E-01
GO:0007599~hemostasis	2.98	1.79E-02	1.88E-01
GO:0050714~positive regulation of protein secretion	4.90	1.83E-02	1.91E-01
GO:0002252~immune effector process	2.70	1.85E-02	1.92E-01
GO:0042531~positive regulation of tyrosine phosphorylation of STAT protein	6.99	1.85E-02	1.91E-01
GO:0010332~response to gamma radiation	6.99	1.85E-02	1.91E-01
GO:0050715~positive regulation of cytokine secretion	6.99	1.85E-02	1.91E-01
GO:0022415~viral reproductive process	3.89	1.86E-02	1.91E-01
GO:0048871~multicellular organismal homeostasis	3.31	1.89E-02	1.93E-01
GO:0060205~cytoplasmic membrane-bounded vesicle lumen	4.85	1.91E-02	3.49E-01
GO:0051051~negative regulation of transport	2.68	1.93E-02	1.96E-01
GO:0060589~nucleoside-triphosphatase regulator activity	1.84	1.93E-02	3.42E-01
GO:0016810~hydrolase activity, acting on carbon-nitrogen (but not peptide) bonds	2.93	1.94E-02	3.34E-01
GO:0016045~detection of bacterium	13.41	1.97E-02	1.99E-01
GO:0075136~response to host	13.41	1.97E-02	1.99E-01
GO:0052200~response to host defenses	13.41	1.97E-02	1.99E-01
GO:0052173~response to defenses of other organism during symbiotic interaction	13.41	1.97E-02	1.99E-01
GO:0048246~macrophage chemotaxis	13.41	1.97E-02	1.99E-01
GO:0046641~positive regulation of alpha-beta T cell proliferation	13.41	1.97E-02	1.99E-01
GO:0010574~regulation of vascular endothelial growth factor production	13.41	1.97E-02	1.99E-01
GO:0045765~regulation of angiogenesis	3.83	1.98E-02	1.99E-01
GO:0010243~response to organic nitrogen	3.83	1.98E-02	1.99E-01
GO:0007249~I-kappaB kinase/NF-kappaB cascade	3.83	1.98E-02	1.99E-01
GO:0051591~response to cAMP	4.79	1.98E-02	1.99E-01
GO:0044403~symbiosis, encompassing mutualism through parasitism	4.79	1.98E-02	1.99E-01
GO:0002443~leukocyte mediated immunity	3.27	1.99E-02	1.99E-01
GO:0051235~maintenance of location	3.77	2.11E-02	2.08E-01
GO:0034599~cellular response to oxidative stress	4.68	2.14E-02	2.11E-01
GO:0031983~vesicle lumen	4.64	2.22E-02	3.71E-01
GO:0001934~positive regulation of protein amino acid phosphorylation	3.16	2.32E-02	2.25E-01
GO:0001910~regulation of leukocyte mediated cytotoxicity	6.43	2.32E-02	2.24E-01
GO:0043331~response to dsRNA	6.43	2.32E-02	2.24E-01
GO:0010743~regulation of foam cell differentiation	6.43	2.32E-02	2.24E-01
GO:0046883~regulation of hormone secretion	3.66	2.37E-02	2.28E-01
GO:0033273~response to vitamin	3.66	2.37E-02	2.28E-01
GO:0016787~hydrolase activity	1.27	2.40E-02	3.86E-01
GO:0007169~transmembrane receptor protein tyrosine kinase signaling pathway	2.15	2.42E-02	2.31E-01

GO:0050829~defense response to Gram-negative bacterium	12.06	2.42E-02	2.30E-01
GO:0042088~T-helper 1 type immune response	12.06	2.42E-02	2.30E-01
GO:0043405~regulation of MAP kinase activity	2.57	2.43E-02	2.30E-01
GO:0044419~interspecies interaction between organisms	1.99	2.45E-02	2.31E-01
GO:0045766~positive regulation of angiogenesis	6.19	2.58E-02	2.41E-01
GO:0046427~positive regulation of JAK-STAT cascade	6.19	2.58E-02	2.41E-01
GO:0000018~regulation of DNA recombination	6.19	2.58E-02	2.41E-01
GO:0046635~positive regulation of alpha-beta T cell activation	6.19	2.58E-02	2.41E-01
GO:0005488~binding	1.05	2.76E-02	4.19E-01
GO:0005829~cytosol	1.38	2.80E-02	4.22E-01
GO:0007242~intracellular signaling cascade	1.38	2.83E-02	2.60E-01
GO:0045428~regulation of nitric oxide biosynthetic process	5.96	2.85E-02	2.61E-01
GO:0051302~regulation of cell division	4.28	2.87E-02	2.62E-01
GO:0031663~lipopolysaccharide-mediated signaling pathway	10.97	2.91E-02	2.64E-01
GO:0043330~response to exogenous dsRNA	10.97	2.91E-02	2.64E-01
GO:0005912~adherens junction	2.48	2.94E-02	4.17E-01
GO:0051259~protein oligomerization	2.31	2.96E-02	2.66E-01
GO:0045637~regulation of myeloid cell differentiation	3.45	2.97E-02	2.67E-01
GO:0048646~anatomical structure formation involved in morphogenesis	1.83	2.99E-02	2.67E-01
GO:0005887~integral to plasma membrane	1.40	3.02E-02	4.06E-01
GO:0014070~response to organic cyclic substance	2.66	3.10E-02	2.75E-01
GO:0030695~GTPase regulator activity	1.77	3.12E-02	4.50E-01
GO:0016032~viral reproduction	3.40	3.13E-02	2.76E-01
GO:0031341~regulation of cell killing	5.75	3.13E-02	2.75E-01
GO:0009653~anatomical structure morphogenesis	1.38	3.22E-02	2.81E-01
GO:0043549~regulation of kinase activity	1.80	3.31E-02	2.86E-01
GO:0042327~positive regulation of phosphorylation	2.90	3.35E-02	2.89E-01
GO:0030856~regulation of epithelial cell differentiation	5.55	3.43E-02	2.94E-01
GO:0050921~positive regulation of chemotaxis	5.55	3.43E-02	2.94E-01
GO:0014823~response to activity	10.05	3.43E-02	2.93E-01
GO:0002675~positive regulation of acute inflammatory response	10.05	3.43E-02	2.93E-01
GO:0031328~positive regulation of cellular biosynthetic process	1.53	3.51E-02	2.98E-01
GO:0010604~positive regulation of macromolecule metabolic process	1.45	3.52E-02	2.98E-01
GO:0008285~negative regulation of cell proliferation	1.78	3.57E-02	3.00E-01
GO:0050864~regulation of B cell activation	3.94	3.73E-02	3.10E-01
GO:0032649~regulation of interferon-gamma production	5.36	3.74E-02	3.10E-01
GO:0010562~positive regulation of phosphorus metabolic process	2.82	3.80E-02	3.13E-01
GO:0045937~positive regulation of phosphate metabolic process	2.82	3.80E-02	3.13E-01
GO:0005924~cell-substrate adherens junction	2.82	3.81E-02	4.64E-01
GO:0051291~protein heterooligomerization	3.87	3.96E-02	3.23E-01
GO:0009891~positive regulation of biosynthetic process	1.50	3.99E-02	3.24E-01
GO:0010744~positive regulation of foam cell differentiation	9.28	3.99E-02	3.23E-01
GO:0045086~positive regulation of interleukin-2 biosynthetic process	9.28	3.99E-02	3.23E-01
GO:0046640~regulation of alpha-beta T cell proliferation	9.28	3.99E-02	3.23E-01
GO:0034612~response to tumor necrosis factor	9.28	3.99E-02	3.23E-01
GO:0042509~regulation of tyrosine phosphorylation of STAT protein	5.19	4.07E-02	3.27E-01
GO:0043900~regulation of multi-organism process	5.19	4.07E-02	3.27E-01
GO:0050920~regulation of chemotaxis	5.19	4.07E-02	3.27E-01
GO:0031226~intrinsic to plasma membrane	1.37	4.08E-02	4.69E-01
GO:0009308~amine metabolic process	1.71	4.15E-02	3.32E-01
GO:0031091~platelet alpha granule	3.81	4.17E-02	4.60E-01
GO:0001568~blood vessel development	1.97	4.22E-02	3.35E-01
GO:0042493~response to drug	2.05	4.27E-02	3.38E-01
GO:0007178~transmembrane receptor protein serine/threonine kinase signaling pa	2.73	4.29E-02	3.38E-01

GO:0030168~platelet activation	5.03	4.41E-02	3.45E-01
GO:0015293~symporter activity	2.46	4.42E-02	5.62E-01
GO:0032355~response to estradiol stimulus	3.72	4.46E-02	3.47E-01
GO:0006919~activation of caspase activity	3.72	4.46E-02	3.47E-01
GO:0002706~regulation of lymphocyte mediated immunity	3.72	4.46E-02	3.47E-01
GO:0019955~cytokine binding	2.71	4.47E-02	5.56E-01
GO:0008283~cell proliferation	1.66	4.47E-02	3.46E-01
GO:0051338~regulation of transferase activity	1.73	4.47E-02	3.46E-01
GO:0044057~regulation of system process	1.82	4.48E-02	3.45E-01
GO:0045069~regulation of viral genome replication	8.62	4.58E-02	3.50E-01
GO:0006027~glycosaminoglycan catabolic process	8.62	4.58E-02	3.50E-01
GO:0046632~alpha-beta T cell differentiation	8.62	4.58E-02	3.50E-01
GO:0043281~regulation of caspase activity	3.05	4.63E-02	3.52E-01
GO:0019962~type I interferon binding	42.18	4.67E-02	5.63E-01
GO:0019964~interferon-gamma binding	42.18	4.67E-02	5.63E-01
GO:0004905~type I interferon receptor activity	42.18	4.67E-02	5.63E-01
GO:0004906~interferon-gamma receptor activity	42.18	4.67E-02	5.63E-01
GO:0002822~regulation of adaptive immune response based on somatic recombina	3.66	4.72E-02	3.57E-01
GO:0022603~regulation of anatomical structure morphogenesis	2.02	4.73E-02	3.56E-01
GO:0045639~positive regulation of myeloid cell differentiation	4.87	4.77E-02	3.58E-01
GO:0030055~cell-substrate junction	2.67	4.77E-02	4.90E-01
GO:0009991~response to extracellular stimulus	2.01	4.78E-02	3.58E-01
GO:0050790~regulation of catalytic activity	1.43	4.80E-02	3.57E-01
GO:0016811~hydrolase activity, acting on carbon-nitrogen (but not peptide) bonds,	3.64	4.81E-02	5.63E-01
GO:0070427~nucleotide-binding oligomerization domain containing 1 signaling pat	40.22	4.90E-02	3.63E-01
GO:0070163~regulation of adiponectin secretion	40.22	4.90E-02	3.63E-01
GO:0034142~toll-like receptor 4 signaling pathway	40.22	4.90E-02	3.63E-01
GO:0001944~vasculature development	1.92	4.90E-02	3.62E-01
GO:0045859~regulation of protein kinase activity	1.75	4.91E-02	3.61E-01
GO:0070161~anchoring junction	2.23	4.94E-02	4.86E-01
GO:0016323~basolateral plasma membrane	2.10	4.96E-02	4.72E-01
GO:0002819~regulation of adaptive immune response	3.59	4.98E-02	3.65E-01
GO:0051054~positive regulation of DNA metabolic process	3.59	4.98E-02	3.65E-01
GO:0048732~gland development	2.38	5.10E-02	3.71E-01
GO:0051173~positive regulation of nitrogen compound metabolic process	1.50	5.11E-02	3.70E-01
GO:0048520~positive regulation of behavior	4.73	5.14E-02	3.71E-01
GO:0006979~response to oxidative stress	2.21	5.18E-02	3.72E-01
GO:0002687~positive regulation of leukocyte migration	8.04	5.20E-02	3.72E-01
GO:0010165~response to X-ray	8.04	5.20E-02	3.72E-01
GO:0032642~regulation of chemokine production	8.04	5.20E-02	3.72E-01
GO:0031625~ubiquitin protein ligase binding	4.69	5.27E-02	5.88E-01
GO:0052548~regulation of endopeptidase activity	2.94	5.29E-02	3.76E-01
GO:0043235~receptor complex	2.57	5.49E-02	4.93E-01
GO:0006575~cellular amino acid derivative metabolic process	2.18	5.49E-02	3.87E-01
GO:0010638~positive regulation of organelle organization	2.91	5.52E-02	3.87E-01
GO:0042803~protein homodimerization activity	1.77	5.52E-02	5.95E-01
GO:0007568~aging	2.56	5.59E-02	3.90E-01
GO:0006519~cellular amino acid and derivative metabolic process	1.71	5.66E-02	3.93E-01
GO:0007611~learning or memory	2.54	5.79E-02	3.99E-01
GO:0044093~positive regulation of molecular function	1.51	5.80E-02	3.99E-01
GO:0043280~positive regulation of caspase activity	3.41	5.83E-02	4.00E-01
GO:0010952~positive regulation of peptidase activity	3.41	5.83E-02	4.00E-01
GO:0032088~negative regulation of NF-kappaB transcription factor activity	7.54	5.85E-02	3.99E-01
GO:0043535~regulation of blood vessel endothelial cell migration	7.54	5.85E-02	3.99E-01

GO:0001776~leukocyte homeostasis	4.47	5.91E-02	4.02E-01
GO:0002263~cell activation during immune response	4.47	5.91E-02	4.02E-01
GO:0046634~regulation of alpha-beta T cell activation	4.47	5.91E-02	4.02E-01
GO:0031331~positive regulation of cellular catabolic process	4.47	5.91E-02	4.02E-01
GO:0046425~regulation of JAK-STAT cascade	4.47	5.91E-02	4.02E-01
GO:0002366~leukocyte activation during immune response	4.47	5.91E-02	4.02E-01
GO:0007584~response to nutrient	2.30	5.98E-02	4.04E-01
GO:0030141~secretory granule	2.13	6.12E-02	5.18E-01
GO:0050878~regulation of body fluid levels	2.28	6.16E-02	4.13E-01
GO:0009314~response to radiation	2.01	6.16E-02	4.12E-01
GO:0007154~cell communication	1.42	6.18E-02	4.12E-01
GO:0052547~regulation of peptidase activity	2.81	6.24E-02	4.14E-01
GO:0031214~biomineral formation	4.35	6.32E-02	4.17E-01
GO:0002703~regulation of leukocyte mediated immunity	3.30	6.44E-02	4.22E-01
GO:0009636~response to toxin	3.30	6.44E-02	4.22E-01
GO:0043200~response to amino acid stimulus	7.10	6.53E-02	4.25E-01
GO:0032732~positive regulation of interleukin-1 production	7.10	6.53E-02	4.25E-01
GO:0070555~response to interleukin-1	7.10	6.53E-02	4.25E-01
GO:0042107~cytokine metabolic process	7.10	6.53E-02	4.25E-01
GO:0032270~positive regulation of cellular protein metabolic process	1.90	6.53E-02	4.25E-01
GO:0001503~ossification	2.45	6.64E-02	4.29E-01
GO:0009986~cell surface	1.72	6.70E-02	5.38E-01
GO:0001818~negative regulation of cytokine production	4.23	6.74E-02	4.33E-01
GO:0006275~regulation of DNA replication	3.24	6.76E-02	4.33E-01
GO:0006302~double-strand break repair	3.24	6.76E-02	4.33E-01
GO:0006508~proteolysis	1.34	6.79E-02	4.33E-01
GO:0005899~insulin receptor complex	28.43	6.85E-02	5.32E-01
GO:0051726~regulation of cell cycle	1.70	6.92E-02	4.39E-01
GO:0003836~beta-galactoside alpha-2,3-sialyltransferase activity	28.12	6.93E-02	6.72E-01
GO:0005085~guanyl-nucleotide exchange factor activity	2.22	6.94E-02	6.64E-01
GO:0045619~regulation of lymphocyte differentiation	3.19	7.08E-02	4.45E-01
GO:0014075~response to amine stimulus	4.12	7.17E-02	4.49E-01
GO:0007613~memory	4.12	7.17E-02	4.49E-01
GO:0051781~positive regulation of cell division	4.12	7.17E-02	4.49E-01
GO:0007259~JAK-STAT cascade	4.12	7.17E-02	4.49E-01
GO:0046631~alpha-beta T cell activation	6.70	7.22E-02	4.50E-01
GO:0002763~positive regulation of myeloid leukocyte differentiation	6.70	7.22E-02	4.50E-01
GO:0009595~detection of biotic stimulus	6.70	7.22E-02	4.50E-01
GO:0060137~maternal process involved in parturition	26.81	7.26E-02	4.51E-01
GO:0001781~neutrophil apoptosis	26.81	7.26E-02	4.51E-01
GO:0014909~smooth muscle cell migration	26.81	7.26E-02	4.51E-01
GO:0033028~myeloid cell apoptosis	26.81	7.26E-02	4.51E-01
GO:0015837~amine transport	2.39	7.33E-02	4.53E-01
GO:0009628~response to abiotic stimulus	1.64	7.36E-02	4.53E-01
GO:0008585~female gonad development	3.14	7.41E-02	4.55E-01
GO:0015294~solute:cation symporter activity	2.66	7.46E-02	6.83E-01
GO:0044106~cellular amine metabolic process	1.71	7.81E-02	4.72E-01
GO:0030246~carbohydrate binding	1.67	7.90E-02	6.96E-01
GO:0045076~regulation of interleukin-2 biosynthetic process	6.35	7.95E-02	4.77E-01
GO:0050926~regulation of positive chemotaxis	6.35	7.95E-02	4.77E-01
GO:0050927~positive regulation of positive chemotaxis	6.35	7.95E-02	4.77E-01
GO:0003690~double-stranded DNA binding	2.61	7.99E-02	6.92E-01
GO:0009055~electron carrier activity	1.91	8.07E-02	6.87E-01
GO:0002761~regulation of myeloid leukocyte differentiation	3.92	8.08E-02	4.82E-01

GO:0051247~positive regulation of protein metabolic process	1.82	8.12E-02	4.83E-01
GO:0005886~plasma membrane	1.14	8.54E-02	6.02E-01
GO:0046651~lymphocyte proliferation	3.83	8.55E-02	5.00E-01
GO:0002768~immune response-regulating cell surface receptor signaling pathway	3.83	8.55E-02	5.00E-01
GO:0060348~bone development	2.29	8.56E-02	4.99E-01
GO:0032570~response to progesterone stimulus	6.03	8.69E-02	5.04E-01
GO:0002685~regulation of leukocyte migration	6.03	8.69E-02	5.04E-01
GO:0042417~dopamine metabolic process	6.03	8.69E-02	5.04E-01
GO:0043434~response to peptide hormone stimulus	2.09	8.89E-02	5.11E-01
GO:0009894~regulation of catabolic process	2.51	9.02E-02	5.15E-01
GO:0030218~erythrocyte differentiation	3.74	9.03E-02	5.14E-01
GO:0019058~viral infectious cycle	3.74	9.03E-02	5.14E-01
GO:0050660~FAD binding	2.93	9.07E-02	7.24E-01
GO:0005925~focal adhesion	2.51	9.10E-02	6.14E-01
GO:0022804~active transmembrane transporter activity	1.63	9.10E-02	7.17E-01
GO:0010557~positive regulation of macromolecule biosynthetic process	1.41	9.11E-02	5.17E-01
GO:0046545~development of primary female sexual characteristics	2.91	9.19E-02	5.19E-01
GO:0046660~female sex differentiation	2.91	9.19E-02	5.19E-01
GO:0016324~apical plasma membrane	2.24	9.21E-02	6.06E-01
GO:0046983~protein dimerization activity	1.48	9.33E-02	7.19E-01
GO:0006576~biogenic amine metabolic process	2.49	9.33E-02	5.23E-01
GO:0048608~reproductive structure development	2.23	9.34E-02	5.23E-01
GO:0031401~positive regulation of protein modification process	1.94	9.42E-02	5.25E-01
GO:0006026~aminoglycan catabolic process	5.75	9.45E-02	5.25E-01
GO:0050792~regulation of viral reproduction	5.75	9.45E-02	5.25E-01
GO:0070304~positive regulation of stress-activated protein kinase signaling pathwa	5.75	9.45E-02	5.25E-01
GO:0002673~regulation of acute inflammatory response	5.75	9.45E-02	5.25E-01
GO:0032403~protein complex binding	1.94	9.50E-02	7.18E-01
GO:0002699~positive regulation of immune effector process	3.66	9.52E-02	5.27E-01
GO:0032943~mononuclear cell proliferation	3.66	9.52E-02	5.27E-01
GO:0070661~leukocyte proliferation	3.66	9.52E-02	5.27E-01
GO:0032276~regulation of gonadotropin secretion	20.11	9.56E-02	5.27E-01
GO:0001780~neutrophil homeostasis	20.11	9.56E-02	5.27E-01
GO:0070423~nucleotide-binding oligomerization domain containing signaling pathv	20.11	9.56E-02	5.27E-01
GO:0032277~negative regulation of gonadotropin secretion	20.11	9.56E-02	5.27E-01
GO:0046882~negative regulation of follicle-stimulating hormone secretion	20.11	9.56E-02	5.27E-01
GO:0070431~nucleotide-binding oligomerization domain containing 2 signaling pat	20.11	9.56E-02	5.27E-01
GO:0046880~regulation of follicle-stimulating hormone secretion	20.11	9.56E-02	5.27E-01
GO:0002548~monocyte chemotaxis	20.11	9.56E-02	5.27E-01
GO:0070391~response to lipoteichoic acid	20.11	9.56E-02	5.27E-01
GO:0070498~interleukin-1-mediated signaling pathway	20.11	9.56E-02	5.27E-01
GO:0002449~lymphocyte mediated immunity	2.87	9.56E-02	5.26E-01
GO:0008201~heparin binding	2.46	9.72E-02	7.20E-01
GO:0051056~regulation of small GTPase mediated signal transduction	1.76	9.76E-02	5.33E-01
GO:0016209~antioxidant activity	3.59	9.94E-02	7.21E-01
