# Supplementary material for: Plasticity of Airway Epithelial Cell Transcriptome in Response to Flagellin
Source: PLoS One. 2015 Feb 10;10(2):e0115486. doi: 10.1371/journal.pone.0115486 (PMC4323341; doi:10.1371/journal.pone.0115486)
Supplement: S4 Table — (PDF) [file pone.0115486.s005.pdf]

Supplementary Table 4. Complete results from functional enrichment analysis of differentially expressed genes following exposure to flagellin in monolayer AEC cultures as identified by RNAseq.

Gene Ontology Annotation	Fold Enrichment	P-value	Adjusted P-value
GO:0002376~immune system process	2.80	3.01E-28	1.01E-24
GO:0006952~defense response	3.41	1.71E-27	2.85E-24
GO:0006955~immune response	3.07	3.58E-24	3.98E-21
GO:0006954~inflammatory response	4.17	2.04E-22	1.70E-19
GO:0009605~response to external stimulus	2.64	4.06E-22	2.71E-19
GO:0009611~response to wounding	3.27	1.36E-21	7.57E-19
GO:0005615~extracellular space	2.95	7.07E-21	2.58E-18
GO:0005125~cytokine activity	5.08	1.11E-19	9.95E-17
GO:0006950~response to stress	2.01	6.35E-19	3.03E-16
GO:0005102~receptor binding	2.46	2.89E-17	1.30E-14
GO:0044421~extracellular region part	2.40	3.13E-17	5.71E-15
GO:0051707~response to other organism	3.87	3.61E-17	1.51E-14
GO:0009607~response to biotic stimulus	3.31	5.49E-16	2.06E-13
GO:0042221~response to chemical stimulus	2.08	1.12E-15	3.71E-13
GO:0048522~positive regulation of cellular process	1.83	5.68E-15	1.72E-12
GO:0048518~positive regulation of biological process	1.77	1.48E-14	4.14E-12
GO:0050896~response to stimulus	1.52	5.75E-14	1.47E-11
GO:0042981~regulation of apoptosis	2.26	1.23E-12	2.92E-10
GO:0043067~regulation of programmed cell death	2.24	2.09E-12	4.65E-10
GO:0010941~regulation of cell death	2.23	2.58E-12	5.39E-10
GO:0009617~response to bacterium	3.95	4.96E-12	9.74E-10
GO:0002237~response to molecule of bacterial origin	5.91	6.16E-12	1.14E-09
GO:0010033~response to organic substance	2.29	9.64E-12	1.69E-09
GO:0051704~multi-organism process	2.33	1.10E-11	1.84E-09
GO:0006915~apoptosis	2.43	1.43E-11	2.27E-09
GO:0005515~protein binding	1.23	1.79E-11	5.35E-09
GO:0012501~programmed cell death	2.39	2.83E-11	4.30E-09
GO:0032496~response to lipopolysaccharide	6.05	3.42E-11	4.96E-09
GO:0051239~regulation of multicellular organismal process	2.06	5.11E-11	7.12E-09
GO:0001817~regulation of cytokine production	3.86	8.20E-11	1.10E-08
GO:0040011~locomotion	2.65	1.33E-10	1.71E-08
GO:0048513~organ development	1.69	2.24E-10	2.77E-08
GO:0045087~innate immune response	4.29	2.35E-10	2.80E-08
GO:0005576~extracellular region	1.65	2.49E-10	3.02E-08
GO:0008219~cell death	2.18	3.62E-10	4.17E-08
GO:0034097~response to cytokine stimulus	5.63	4.34E-10	4.83E-08
GO:0016265~death	2.16	4.96E-10	5.34E-08
GO:0042127~regulation of cell proliferation	2.10	6.28E-10	6.55E-08
GO:0008009~chemokine activity	7.49	1.13E-09	2.54E-07
GO:0048731~system development	1.54	1.53E-09	1.55E-07
GO:0002682~regulation of immune system process	2.64	1.96E-09	1.93E-07
GO:0048583~regulation of response to stimulus	2.46	2.12E-09	2.02E-07
GO:0016477~cell migration	2.99	2.62E-09	2.43E-07
GO:0042379~chemokine receptor binding	7.03	3.06E-09	5.50E-07
GO:0010646~regulation of cell communication	1.88	4.47E-09	4.04E-07
GO:0050793~regulation of developmental process	2.14	4.83E-09	4.25E-07
GO:0048870~cell motility	2.83	5.05E-09	4.33E-07
GO:0051674~localization of cell	2.83	5.05E-09	4.33E-07
GO:0080134~regulation of response to stress	2.94	7.27E-09	6.07E-07
GO:0070482~response to oxygen levels	3.90	9.13E-09	7.44E-07
GO:0008544~epidermis development	3.45	9.85E-09	7.83E-07
GO:0031347~regulation of defense response	3.85	1.23E-08	9.54E-07
GO:0007398~ectoderm development	3.30	1.58E-08	1.20E-06
GO:0004896~cytokine receptor activity	6.27	1.78E-08	2.67E-06

GO:0006935~chemotaxis	3.57	3.02E-08	2.24E-06
GO:0042330~taxis	3.57	3.02E-08	2.24E-06
GO:0009966~regulation of signal transduction	1.90	3.67E-08	2.66E-06
GO:0050900~leukocyte migration	5.94	3.80E-08	2.70E-06
GO:0048856~anatomical structure development	1.47	3.94E-08	2.74E-06
GO:0048545~response to steroid hormone stimulus	3.20	9.80E-08	6.68E-06
GO:0010647~positive regulation of cell communication	2.57	1.06E-07	7.08E-06
GO:0051094~positive regulation of developmental process	2.74	1.10E-07	7.23E-06
GO:0065007~biological regulation	1.18	1.26E-07	8.10E-06
GO:0009615~response to virus	4.08	1.55E-07	9.77E-06
GO:0002684~positive regulation of immune system process	2.85	2.77E-07	1.71E-05
GO:0051090~regulation of transcription factor activity	4.11	2.90E-07	1.76E-05
GO:0050794~regulation of cellular process	1.20	3.01E-07	1.79E-05
GO:0032502~developmental process	1.37	3.23E-07	1.89E-05
GO:0006916~anti-apoptosis	2.98	4.36E-07	2.51E-05
GO:0043065~positive regulation of apoptosis	2.26	4.36E-07	2.47E-05
GO:0051240~positive regulation of multicellular organismal process	2.78	4.81E-07	2.68E-05
GO:0043068~positive regulation of programmed cell death	2.25	5.17E-07	2.83E-05
GO:0032879~regulation of localization	2.01	5.77E-07	3.11E-05
GO:0010942~positive regulation of cell death	2.24	6.01E-07	3.19E-05
GO:0051272~positive regulation of cell motion	4.10	6.35E-07	3.32E-05
GO:0040017~positive regulation of locomotion	4.10	6.35E-07	3.32E-05
GO:0030595~leukocyte chemotaxis	6.86	6.60E-07	3.39E-05
GO:0048519~negative regulation of biological process	1.52	6.77E-07	3.42E-05
GO:0051050~positive regulation of transport	2.85	6.96E-07	3.47E-05
GO:0001775~cell activation	2.58	7.04E-07	3.46E-05
GO:0030335~positive regulation of cell migration	4.28	7.30E-07	3.53E-05
GO:0001664~G-protein-coupled receptor binding	3.88	7.49E-07	9.61E-05
GO:0007275~multicellular organismal development	1.38	8.38E-07	4.00E-05
GO:0001819~positive regulation of cytokine production	4.23	8.61E-07	4.05E-05
GO:0001944~vasculature development	2.70	8.96E-07	4.16E-05
GO:0001666~response to hypoxia	3.47	1.18E-06	5.42E-05
GO:0060326~cell chemotaxis	6.51	1.19E-06	5.36E-05
GO:0050789~regulation of biological process	1.18	1.20E-06	5.35E-05
GO:0040012~regulation of locomotion	2.98	1.21E-06	5.30E-05
GO:0030334~regulation of cell migration	3.13	1.31E-06	5.67E-05
GO:0009967~positive regulation of signal transduction	2.51	1.34E-06	5.73E-05
GO:0009719~response to endogenous stimulus	2.25	1.34E-06	5.68E-05
GO:0005488~binding	1.08	1.36E-06	1.52E-04
GO:0045595~regulation of cell differentiation	2.11	1.39E-06	5.79E-05
GO:0001568~blood vessel development	2.68	1.61E-06	6.63E-05
GO:0030855~epithelial cell differentiation	3.40	1.71E-06	6.98E-05
GO:0043066~negative regulation of apoptosis	2.33	1.88E-06	7.56E-05
GO:0048584~positive regulation of response to stimulus	2.69	2.23E-06	8.86E-05
GO:0008284~positive regulation of cell proliferation	2.20	2.38E-06	9.35E-05
GO:0043122~regulation of I-kappaB kinase/NF-kappaB cascade	3.76	2.41E-06	9.35E-05
GO:0019955~cytokine binding	3.75	2.50E-06	2.49E-04
GO:0043069~negative regulation of programmed cell death	2.30	2.64E-06	1.01E-04
GO:0006928~cell motion	2.09	2.82E-06	1.07E-04
GO:0060548~negative regulation of cell death	2.29	2.84E-06	1.07E-04
GO:0051101~regulation of DNA binding	3.50	3.68E-06	1.37E-04
GO:0010740~positive regulation of protein kinase cascade	3.04	3.69E-06	1.36E-04
GO:0004908~interleukin-1 receptor activity	18.46	4.10E-06	3.69E-04
GO:0009888~tissue development	1.88	4.13E-06	1.50E-04
GO:0051270~regulation of cell motion	2.85	4.35E-06	1.56E-04
GO:0009725~response to hormone stimulus	2.25	4.47E-06	1.59E-04
GO:0008083~growth factor activity	3.08	5.21E-06	4.26E-04
GO:0048534~hemopoietic or lymphoid organ development	2.52	5.46E-06	1.92E-04

GO:0032101~regulation of response to external stimulus	3.06	5.54E-06	1.93E-04
GO:0043433~negative regulation of transcription factor activity	5.64	5.55E-06	1.91E-04
GO:0007626~locomotory behavior	2.47	5.74E-06	1.96E-04
GO:0030097~hemopoiesis	2.60	6.57E-06	2.22E-04
GO:0031349~positive regulation of defense response	4.35	6.59E-06	2.20E-04
GO:0002520~immune system development	2.45	6.67E-06	2.20E-04
GO:0006917~induction of apoptosis	2.31	8.16E-06	2.67E-04
GO:0012502~induction of programmed cell death	2.31	8.74E-06	2.83E-04
GO:0008283~cell proliferation	2.09	8.74E-06	2.81E-04
GO:0032946~positive regulation of mononuclear cell proliferation	4.91	9.10E-06	2.89E-04
GO:0070665~positive regulation of leukocyte proliferation	4.91	9.10E-06	2.89E-04
GO:0051098~regulation of binding	3.04	1.02E-05	3.22E-04
GO:0050727~regulation of inflammatory response	4.18	1.07E-05	3.35E-04
GO:0045597~positive regulation of cell differentiation	2.59	1.08E-05	3.32E-04
GO:0043123~positive regulation of I-kappaB kinase/NF-kappaB cascade	3.71	1.13E-05	3.45E-04
GO:0051222~positive regulation of protein transport	4.42	1.23E-05	3.73E-04
GO:0007243~protein kinase cascade	2.17	1.31E-05	3.94E-04
GO:0048869~cellular developmental process	1.46	1.51E-05	4.51E-04
GO:0050867~positive regulation of cell activation	3.43	1.66E-05	4.92E-04
GO:0008285~negative regulation of cell proliferation	2.17	1.82E-05	5.34E-04
GO:0045785~positive regulation of cell adhesion	4.59	1.92E-05	5.57E-04
GO:0043392~negative regulation of DNA binding	4.98	2.01E-05	5.79E-04
GO:0019221~cytokine-mediated signaling pathway	4.23	2.02E-05	5.75E-04
GO:0042834~peptidoglycan binding	14.36	2.28E-05	1.70E-03
GO:0051223~regulation of protein transport	3.34	2.38E-05	6.73E-04
GO:0050776~regulation of immune response	2.53	2.43E-05	6.81E-04
GO:0019838~growth factor binding	3.49	2.54E-05	1.76E-03
GO:0030154~cell differentiation	1.46	2.61E-05	7.25E-04
GO:0009891~positive regulation of biosynthetic process	1.77	3.07E-05	8.47E-04
GO:0007165~signal transduction	1.32	3.35E-05	9.16E-04
GO:0007242~intracellular signaling cascade	1.53	3.50E-05	9.51E-04
GO:0002696~positive regulation of leukocyte activation	3.39	3.53E-05	9.49E-04
GO:0031325~positive regulation of cellular metabolic process	1.66	3.61E-05	9.64E-04
GO:0065008~regulation of biological quality	1.48	3.61E-05	9.56E-04
GO:0042035~regulation of cytokine biosynthetic process	4.00	3.74E-05	9.82E-04
GO:0032103~positive regulation of response to external stimulus	4.30	3.79E-05	9.88E-04
GO:0031328~positive regulation of cellular biosynthetic process	1.76	4.01E-05	1.04E-03
GO:0051049~regulation of transport	1.99	4.15E-05	1.07E-03
GO:0050671~positive regulation of lymphocyte proliferation	4.62	4.25E-05	1.08E-03
GO:0019966~interleukin-1 binding	12.92	4.38E-05	2.81E-03
GO:0051251~positive regulation of lymphocyte activation	3.49	4.61E-05	1.17E-03
GO:0009893~positive regulation of metabolic process	1.63	4.62E-05	1.16E-03
GO:0010627~regulation of protein kinase cascade	2.38	4.83E-05	1.20E-03
GO:0070201~regulation of establishment of protein localization	3.15	5.20E-05	1.29E-03
GO:0030216~keratinocyte differentiation	4.17	5.21E-05	1.28E-03
GO:0032501~multicellular organismal process	1.23	5.25E-05	1.28E-03
GO:0030234~enzyme regulator activity	1.67	5.59E-05	3.35E-03
GO:0001525~angiogenesis	2.86	6.82E-05	1.65E-03
GO:0002544~chronic inflammatory response	17.64	6.83E-05	1.64E-03
GO:0050708~regulation of protein secretion	4.38	7.12E-05	1.70E-03
GO:0060429~epithelium development	2.42	7.14E-05	1.69E-03
GO:0065009~regulation of molecular function	1.59	7.47E-05	1.76E-03
GO:0048523~negative regulation of cellular process	1.43	7.57E-05	1.77E-03
GO:0043281~regulation of caspase activity	3.75	7.59E-05	1.76E-03
GO:0050865~regulation of cell activation	2.66	7.84E-05	1.80E-03
GO:0006953~acute-phase response	5.29	8.04E-05	1.84E-03
GO:0005539~glycosaminoglycan binding	2.92	8.32E-05	4.66E-03
GO:0051100~negative regulation of binding	4.30	8.38E-05	1.90E-03

GO:0032655~regulation of interleukin-12 production	8.71	8.53E-05	1.92E-03
GO:0032880~regulation of protein localization	2.91	8.55E-05	1.91E-03
GO:0001871~pattern binding	2.80	9.37E-05	4.94E-03
GO:0030247~polysaccharide binding	2.80	9.37E-05	4.94E-03
GO:0051092~positive regulation of NF-kappaB transcription factor activity	5.16	9.88E-05	2.20E-03
GO:0050714~positive regulation of protein secretion	5.16	9.88E-05	2.20E-03
GO:0042060~wound healing	2.55	9.89E-05	2.18E-03
GO:0007610~behavior	1.90	1.04E-04	2.28E-03
GO:0019220~regulation of phosphate metabolic process	1.88	1.06E-04	2.31E-03
GO:0051174~regulation of phosphorus metabolic process	1.88	1.06E-04	2.31E-03
GO:0022603~regulation of anatomical structure morphogenesis	2.42	1.07E-04	2.32E-03
GO:0002694~regulation of leukocyte activation	2.68	1.09E-04	2.35E-03
GO:0030099~myeloid cell differentiation	3.41	1.11E-04	2.36E-03
GO:0052548~regulation of endopeptidase activity	3.61	1.13E-04	2.39E-03
GO:0030593~neutrophil chemotaxis	8.23	1.23E-04	2.60E-03
GO:0009913~epidermal cell differentiation	3.82	1.25E-04	2.63E-03
GO:0005887~integral to plasma membrane	1.52	1.44E-04	1.30E-02
GO:0032944~regulation of mononuclear cell proliferation	3.53	1.45E-04	3.02E-03
GO:0070663~regulation of leukocyte proliferation	3.53	1.45E-04	3.02E-03
GO:0032494~response to peptidoglycan	15.12	1.53E-04	3.18E-03
GO:0048247~lymphocyte chemotaxis	15.12	1.53E-04	3.18E-03
GO:0048514~blood vessel morphogenesis	2.41	1.60E-04	3.30E-03
GO:0031960~response to corticosteroid stimulus	3.49	1.64E-04	3.35E-03
GO:0051047~positive regulation of secretion	3.11	1.79E-04	3.64E-03
GO:0042325~regulation of phosphorylation	1.86	1.83E-04	3.69E-03
GO:0031226~intrinsic to plasma membrane	1.50	1.84E-04	1.33E-02
GO:0052547~regulation of peptidase activity	3.45	1.85E-04	3.71E-03
GO:0002526~acute inflammatory response	3.24	1.96E-04	3.91E-03
GO:0045321~leukocyte activation	2.27	1.96E-04	3.89E-03
GO:0051249~regulation of lymphocyte activation	2.72	2.11E-04	4.15E-03
GO:0050870~positive regulation of T cell activation	3.62	2.13E-04	4.18E-03
GO:0034612~response to tumor necrosis factor	9.77	2.16E-04	4.21E-03
GO:0032675~regulation of interleukin-6 production	5.29	2.20E-04	4.26E-03
GO:0032570~response to progesterone stimulus	7.41	2.37E-04	4.56E-03
GO:0048872~homeostasis of number of cells	3.17	2.43E-04	4.65E-03
GO:0030155~regulation of cell adhesion	2.78	2.46E-04	4.68E-03
GO:0042592~homeostatic process	1.63	2.51E-04	4.74E-03
GO:0002697~regulation of immune effector process	3.14	2.70E-04	5.09E-03
GO:0055093~response to hyperoxia	13.23	2.95E-04	5.52E-03
GO:0045787~positive regulation of cell cycle	4.08	2.95E-04	5.49E-03
GO:0008329~pattern recognition receptor activity	9.23	2.98E-04	1.48E-02
GO:0070304~positive regulation of stress-activated protein kinase signaling pathway	7.05	3.18E-04	5.89E-03
GO:0001533~cornified envelope	6.99	3.44E-04	2.07E-02
GO:0051173~positive regulation of nitrogen compound metabolic process	1.68	3.50E-04	6.44E-03
GO:0007167~enzyme linked receptor protein signaling pathway	1.98	3.82E-04	6.99E-03
GO:0019961~interferon binding	21.54	3.83E-04	1.80E-02
GO:0004904~interferon receptor activity	21.54	3.83E-04	1.80E-02
GO:0007259~JAK-STAT cascade	4.88	3.95E-04	7.18E-03
GO:0050707~regulation of cytokine secretion	5.64	3.96E-04	7.17E-03
GO:0050729~positive regulation of inflammatory response	5.64	3.96E-04	7.17E-03
GO:0043627~response to estrogen stimulus	3.02	4.06E-04	7.31E-03
GO:0045682~regulation of epidermis development	6.73	4.20E-04	7.52E-03
GO:0051347~positive regulation of transferase activity	2.20	4.23E-04	7.53E-03
GO:0005886~plasma membrane	1.23	4.50E-04	2.32E-02
GO:0051091~positive regulation of transcription factor activity	3.88	4.54E-04	8.03E-03
GO:0044093~positive regulation of molecular function	1.70	4.75E-04	8.36E-03
GO:0050670~regulation of lymphocyte proliferation	3.31	4.93E-04	8.63E-03
GO:0032874~positive regulation of stress-activated MAPK cascade	11.76	5.12E-04	8.91E-03

GO:0050715~positive regulation of cytokine secretion	6.44	5.46E-04	9.46E-03
GO:0051046~regulation of secretion	2.30	5.76E-04	9.93E-03
GO:0033674~positive regulation of kinase activity	2.20	5.86E-04	1.00E-02
GO:0005149~interleukin-1 receptor binding	8.08	6.01E-04	2.67E-02
GO:0042327~positive regulation of phosphorylation	3.05	6.16E-04	1.05E-02
GO:0060341~regulation of cellular localization	2.13	6.79E-04	1.15E-02
GO:0051241~negative regulation of multicellular organismal process	2.45	7.39E-04	1.25E-02
GO:0042742~defense response to bacterium	2.83	7.86E-04	1.32E-02
GO:0045121~membrane raft	2.61	7.90E-04	3.54E-02
GO:0010604~positive regulation of macromolecule metabolic process	1.53	8.00E-04	1.33E-02
GO:0032872~regulation of stress-activated MAPK cascade	10.58	8.21E-04	1.36E-02
GO:0045937~positive regulation of phosphate metabolic process	2.96	8.25E-04	1.36E-02
GO:0010562~positive regulation of phosphorus metabolic process	2.96	8.25E-04	1.36E-02
GO:0006919~activation of caspase activity	3.92	8.68E-04	1.42E-02
GO:0010557~positive regulation of macromolecule biosynthetic process	1.62	8.80E-04	1.44E-02
GO:0070555~response to interleukin-1	7.47	8.87E-04	1.44E-02
GO:0002221~pattern recognition receptor signaling pathway	7.47	8.87E-04	1.44E-02
GO:0002573~myeloid leukocyte differentiation	4.98	8.89E-04	1.44E-02
GO:0051130~positive regulation of cellular component organization	2.34	9.21E-04	1.48E-02
GO:0007169~transmembrane receptor protein tyrosine kinase signaling pathway	2.17	9.29E-04	1.49E-02
GO:0051384~response to glucocorticoid stimulus	3.26	1.03E-03	1.65E-02
GO:0004867~serine-type endopeptidase inhibitor activity	3.04	1.08E-03	4.53E-02
GO:0005161~platelet-derived growth factor receptor binding	9.79	1.16E-03	4.65E-02
GO:0050863~regulation of T cell activation	2.71	1.21E-03	1.92E-02
GO:0043330~response to exogenous dsRNA	9.62	1.24E-03	1.96E-02
GO:0018108~peptidyl-tyrosine phosphorylation	4.14	1.25E-03	1.96E-02
GO:0002521~leukocyte differentiation	2.58	1.30E-03	2.02E-02
GO:0051338~regulation of transferase activity	1.82	1.53E-03	2.37E-02
GO:0032755~positive regulation of interleukin-6 production	6.68	1.54E-03	2.38E-02
GO:0002758~innate immune response-activating signal transduction	6.68	1.54E-03	2.38E-02
GO:0002218~activation of innate immune response	6.68	1.54E-03	2.38E-02
GO:0046626~regulation of insulin receptor signaling pathway	6.68	1.54E-03	2.38E-02
GO:0051789~response to protein stimulus	2.77	1.56E-03	2.39E-02
GO:0043388~positive regulation of DNA binding	3.33	1.56E-03	2.39E-02
GO:0043549~regulation of kinase activity	1.84	1.60E-03	2.43E-02
GO:0019904~protein domain specific binding	1.89	1.61E-03	6.10E-02
GO:0043280~positive regulation of caspase activity	3.59	1.66E-03	2.52E-02
GO:0010952~positive regulation of peptidase activity	3.59	1.66E-03	2.52E-02
GO:0018212~peptidyl-tyrosine modification	3.97	1.67E-03	2.51E-02
GO:0043410~positive regulation of MAPKKK cascade	3.97	1.67E-03	2.51E-02
GO:0042108~positive regulation of cytokine biosynthetic process	3.97	1.67E-03	2.51E-02
GO:0014070~response to organic cyclic substance	2.62	1.68E-03	2.52E-02
GO:0010035~response to inorganic substance	2.17	1.69E-03	2.52E-02
GO:0010638~positive regulation of organelle organization	3.06	1.74E-03	2.58E-02
GO:0045935~positive regulation of nucleobase, nucleoside, nucleotide and nucleic acid	1.59	1.75E-03	2.59E-02
GO:0051051~negative regulation of transport	2.51	1.75E-03	2.59E-02
GO:0001818~negative regulation of cytokine production	4.46	1.77E-03	2.60E-02
GO:0002483~antigen processing and presentation of endogenous peptide antigen	14.11	1.88E-03	2.74E-02
GO:0019885~antigen processing and presentation of endogenous peptide antigen via M	14.11	1.88E-03	2.74E-02
GO:0045071~negative regulation of viral genome replication	14.11	1.88E-03	2.74E-02
GO:0050830~defense response to Gram-positive bacterium	6.35	1.98E-03	2.87E-02
GO:0030574~collagen catabolic process	6.35	1.98E-03	2.87E-02
GO:0030856~regulation of epithelial cell differentiation	5.11	2.02E-03	2.92E-02
GO:0045860~positive regulation of protein kinase activity	2.09	2.03E-03	2.92E-02
GO:0042102~positive regulation of T cell proliferation	4.34	2.07E-03	2.97E-02
GO:0048871~multicellular organismal homeostasis	2.99	2.11E-03	3.00E-02
GO:0044459~plasma membrane part	1.28	2.34E-03	9.05E-02
GO:0042110~T cell activation	2.52	2.46E-03	3.49E-02

GO:0045429~positive regulation of nitric oxide biosynthetic process	6.05	2.49E-03	3.52E-02
GO:0009628~response to abiotic stimulus	1.78	2.52E-03	3.53E-02
GO:0008217~regulation of blood pressure	2.75	2.58E-03	3.61E-02
GO:0050790~regulation of catalytic activity	1.48	2.59E-03	3.60E-02
GO:0046649~lymphocyte activation	2.13	2.78E-03	3.85E-02
GO:0002761~regulation of myeloid leukocyte differentiation	4.13	2.79E-03	3.85E-02
GO:0048661~positive regulation of smooth muscle cell proliferation	4.78	2.89E-03	3.96E-02
GO:0004866~endopeptidase inhibitor activity	2.38	3.00E-03	1.06E-01
GO:0001934~positive regulation of protein amino acid phosphorylation	2.85	3.04E-03	4.15E-02
GO:0009653~anatomical structure morphogenesis	1.38	3.07E-03	4.17E-02
GO:0008015~blood circulation	2.16	3.07E-03	4.15E-02
GO:0003013~circulatory system process	2.16	3.07E-03	4.15E-02
GO:0022804~active transmembrane transporter activity	1.78	3.09E-03	1.05E-01
GO:0048525~negative regulation of viral reproduction	12.09	3.17E-03	4.27E-02
GO:0060558~regulation of calcidiol 1-monooxygenase activity	12.09	3.17E-03	4.27E-02
GO:0002224~toll-like receptor signaling pathway	7.56	3.36E-03	4.51E-02
GO:0045069~regulation of viral genome replication	7.56	3.36E-03	4.51E-02
GO:0051099~positive regulation of binding	2.98	3.54E-03	4.72E-02
GO:0015291~secondary active transmembrane transporter activity	2.08	3.57E-03	1.16E-01
GO:0045088~regulation of innate immune response	3.53	3.58E-03	4.76E-02
GO:0032355~response to estradiol stimulus	3.53	3.58E-03	4.76E-02
GO:0045859~regulation of protein kinase activity	1.78	3.67E-03	4.85E-02
GO:0031424~keratinization	3.94	3.69E-03	4.86E-02
GO:0004857~enzyme inhibitor activity	1.91	3.70E-03	1.16E-01
GO:0032652~regulation of interleukin-1 production	5.52	3.81E-03	5.00E-02
GO:0009991~response to extracellular stimulus	2.02	3.84E-03	5.01E-02
GO:0006959~humoral immune response	2.95	3.89E-03	5.05E-02
GO:0051726~regulation of cell cycle	1.79	4.00E-03	5.17E-02
GO:0046983~protein dimerization activity	1.59	4.30E-03	1.29E-01
GO:0046627~negative regulation of insulin receptor signaling pathway	7.05	4.42E-03	5.68E-02
GO:0032642~regulation of chemokine production	7.05	4.42E-03	5.68E-02
GO:0015294~solute:cation symporter activity	2.72	4.44E-03	1.29E-01
GO:0002764~immune response-regulating signal transduction	3.40	4.51E-03	5.77E-02
GO:0015293~symporter activity	2.36	4.54E-03	1.27E-01
GO:0022600~digestive system process	4.36	4.68E-03	5.96E-02
GO:0050806~positive regulation of synaptic transmission	4.36	4.68E-03	5.96E-02
GO:0019883~antigen processing and presentation of endogenous antigen	10.58	4.90E-03	6.21E-02
GO:0030414~peptidase inhibitor activity	2.25	4.98E-03	1.35E-01
GO:0051336~regulation of hydrolase activity	1.76	5.08E-03	6.41E-02
GO:0016323~basolateral plasma membrane	2.06	5.27E-03	1.75E-01
GO:0045089~positive regulation of innate immune response	3.68	5.42E-03	6.80E-02
GO:0048660~regulation of smooth muscle cell proliferation	3.68	5.42E-03	6.80E-02
GO:0001816~cytokine production	3.68	5.42E-03	6.80E-02
GO:0045637~regulation of myeloid cell differentiation	3.02	5.44E-03	6.79E-02
GO:0045840~positive regulation of mitosis	5.08	5.57E-03	6.93E-02
GO:0051785~positive regulation of nuclear division	5.08	5.57E-03	6.93E-02
GO:0043331~response to dsRNA	5.08	5.57E-03	6.93E-02
GO:0033280~response to vitamin D	6.61	5.67E-03	7.02E-02
GO:0032088~negative regulation of NF-kappaB transcription factor activity	6.61	5.67E-03	7.02E-02
GO:0044057~regulation of system process	1.78	6.05E-03	7.44E-02
GO:0007584~response to nutrient	2.27	6.38E-03	7.80E-02
GO:0060137~maternal process involved in parturition	21.16	6.46E-03	7.87E-02
GO:0044243~multicellular organismal catabolic process	4.88	6.63E-03	8.05E-02
GO:0042493~response to drug	1.96	6.76E-03	8.16E-02
GO:0010212~response to ionizing radiation	3.17	6.89E-03	8.28E-02
GO:0045684~positive regulation of epidermis development	9.41	7.09E-03	8.49E-02
GO:0044060~regulation of endocrine process	9.41	7.09E-03	8.49E-02
GO:0016045~detection of bacterium	9.41	7.09E-03	8.49E-02

GO:0002474~antigen processing and presentation of peptide antigen via MHC class I	6.22	7.15E-03	8.52E-02
GO:0032732~positive regulation of interleukin-1 production	6.22	7.15E-03	8.52E-02
GO:0051971~positive regulation of transmission of nerve impulse	4.00	7.18E-03	8.53E-02
GO:0008047~enzyme activator activity	1.74	7.21E-03	1.84E-01
GO:0048585~negative regulation of response to stimulus	2.54	7.42E-03	8.77E-02
GO:0051052~regulation of DNA metabolic process	2.41	7.49E-03	8.82E-02
GO:0045177~apical part of cell	2.09	7.65E-03	2.25E-01
GO:0045428~regulation of nitric oxide biosynthetic process	4.70	7.83E-03	9.17E-02
GO:0008201~heparin binding	2.51	8.14E-03	2.00E-01
GO:0035295~tube development	1.92	8.17E-03	9.52E-02
GO:0043085~positive regulation of catalytic activity	1.55	8.30E-03	9.63E-02
GO:0016638~oxidoreductase activity, acting on the CH-NH2 group of donors	5.98	8.33E-03	1.98E-01
GO:0042129~regulation of T cell proliferation	3.07	8.39E-03	9.69E-02
GO:0006275~regulation of DNA replication	3.07	8.39E-03	9.69E-02
GO:0006814~sodium ion transport	2.28	8.40E-03	9.67E-02
GO:0050817~coagulation	2.49	8.58E-03	9.83E-02
GO:0007596~blood coagulation	2.49	8.58E-03	9.83E-02
GO:0050778~positive regulation of immune response	2.19	8.62E-03	9.84E-02
GO:0004222~metalloendopeptidase activity	2.49	8.73E-03	2.02E-01
GO:0005901~caveola	3.38	8.77E-03	2.35E-01
GO:0009595~detection of biotic stimulus	5.88	8.85E-03	1.01E-01
GO:0002763~positive regulation of myeloid leukocyte differentiation	5.88	8.85E-03	1.01E-01
GO:0048646~anatomical structure formation involved in morphogenesis	1.68	8.90E-03	1.01E-01
GO:0009986~cell surface	1.70	9.12E-03	2.27E-01
GO:0032963~collagen metabolic process	4.54	9.17E-03	1.03E-01
GO:0045740~positive regulation of DNA replication	4.54	9.17E-03	1.03E-01
GO:0048002~antigen processing and presentation of peptide antigen	4.54	9.17E-03	1.03E-01
GO:0007050~cell cycle arrest	2.47	9.21E-03	1.03E-01
GO:0045765~regulation of angiogenesis	3.02	9.22E-03	1.03E-01
GO:0010243~response to organic nitrogen	3.02	9.22E-03	1.03E-01
GO:0007249~I-kappaB kinase/NF-kappaB cascade	3.02	9.22E-03	1.03E-01
GO:0001894~tissue homeostasis	3.02	9.22E-03	1.03E-01
GO:0031646~positive regulation of neurological system process	3.80	9.31E-03	1.04E-01
GO:0015837~amine transport	2.33	9.77E-03	1.08E-01
GO:0045073~regulation of chemokine biosynthetic process	8.47	9.78E-03	1.08E-01
GO:0010829~negative regulation of glucose transport	8.47	9.78E-03	1.08E-01
GO:0034341~response to interferon-gamma	8.47	9.78E-03	1.08E-01
GO:0045123~cellular extravasation	8.47	9.78E-03	1.08E-01
GO:0001558~regulation of cell growth	1.96	1.04E-02	1.14E-01
GO:0007586~digestion	2.56	1.05E-02	1.15E-01
GO:0002757~immune response-activating signal transduction	3.26	1.06E-02	1.16E-01
GO:0009897~external side of plasma membrane	2.07	1.08E-02	2.46E-01
GO:0007267~cell-cell signaling	1.48	1.08E-02	1.17E-01
GO:0048008~platelet-derived growth factor receptor signaling pathway	5.57	1.08E-02	1.17E-01
GO:0051093~negative regulation of developmental process	1.80	1.10E-02	1.19E-01
GO:0033256~I-kappaB/NF-kappaB complex	16.48	1.16E-02	2.48E-01
GO:0031667~response to nutrient levels	1.93	1.20E-02	1.28E-01
GO:0033273~response to vitamin	2.89	1.21E-02	1.29E-01
GO:0008745~N-acetylmuramoyl-L-alanine amidase activity	16.16	1.21E-02	2.62E-01
GO:0042990~regulation of transcription factor import into nucleus	4.23	1.23E-02	1.30E-01
GO:0070431~nucleotide-binding oligomerization domain containing 2 signaling pathway	15.87	1.25E-02	1.32E-01
GO:0070423~nucleotide-binding oligomerization domain containing signaling pathway	15.87	1.25E-02	1.32E-01
GO:0070498~interleukin-1-mediated signaling pathway	15.87	1.25E-02	1.32E-01
GO:0014805~smooth muscle adaptation	15.87	1.25E-02	1.32E-01
GO:0007599~hemostasis	2.35	1.29E-02	1.36E-01
GO:0030055~cell-substrate junction	2.35	1.29E-02	2.57E-01
GO:0007567~parturition	7.70	1.30E-02	1.36E-01
GO:0031663~lipopolysaccharide-mediated signaling pathway	7.70	1.30E-02	1.36E-01

GO:0006805~xenobiotic metabolic process	5.29	1.30E-02	1.36E-01
GO:0042417~dopamine metabolic process	5.29	1.30E-02	1.36E-01
GO:0002685~regulation of leukocyte migration	5.29	1.30E-02	1.36E-01
GO:0019992~diacylglycerol binding	2.85	1.30E-02	2.73E-01
GO:0032813~tumor necrosis factor receptor superfamily binding	4.17	1.32E-02	2.69E-01
GO:0006800~oxygen and reactive oxygen species metabolic process	2.84	1.32E-02	1.37E-01
GO:0051591~response to cAMP	3.53	1.33E-02	1.38E-01
GO:0017124~SH3 domain binding	2.47	1.34E-02	2.67E-01
GO:0005096~GTPase activator activity	1.86	1.41E-02	2.73E-01
GO:0043900~regulation of multi-organism process	4.10	1.41E-02	1.45E-01
GO:0008637~apoptotic mitochondrial changes	4.10	1.41E-02	1.45E-01
GO:0044259~multicellular organismal macromolecule metabolic process	4.10	1.41E-02	1.45E-01
GO:0003700~transcription factor activity	1.35	1.45E-02	2.73E-01
GO:0005164~tumor necrosis factor receptor binding	5.13	1.46E-02	2.70E-01
GO:0045926~negative regulation of growth	2.31	1.47E-02	1.50E-01
GO:0007565~female pregnancy	2.31	1.47E-02	1.50E-01
GO:0044092~negative regulation of molecular function	1.65	1.53E-02	1.55E-01
GO:0050792~regulation of viral reproduction	5.04	1.55E-02	1.57E-01
GO:0001569~patterning of blood vessels	5.04	1.55E-02	1.57E-01
GO:0001836~release of cytochrome c from mitochondria	5.04	1.55E-02	1.57E-01
GO:0051054~positive regulation of DNA metabolic process	3.02	1.57E-02	1.58E-01
GO:0004175~endopeptidase activity	1.61	1.59E-02	2.84E-01
GO:0055080~cation homeostasis	1.70	1.66E-02	1.66E-01
GO:0008624~induction of apoptosis by extracellular signals	2.27	1.66E-02	1.66E-01
GO:0002675~positive regulation of acute inflammatory response	7.05	1.67E-02	1.66E-01
GO:0010039~response to iron ion	7.05	1.67E-02	1.66E-01
GO:0035239~tube morphogenesis	2.17	1.68E-02	1.67E-01
GO:0003714~transcription corepressor activity	2.08	1.73E-02	3.00E-01
GO:0005925~focal adhesion	2.37	1.75E-02	3.16E-01
GO:0016810~hydrolase activity, acting on carbon-nitrogen (but not peptide) bonds	2.25	1.77E-02	3.00E-01
GO:0016324~apical plasma membrane	2.15	1.81E-02	3.09E-01
GO:0008289~lipid binding	1.53	1.81E-02	3.00E-01
GO:0045639~positive regulation of myeloid cell differentiation	3.85	1.83E-02	1.79E-01
GO:0042326~negative regulation of phosphorylation	3.29	1.83E-02	1.79E-01
GO:0032147~activation of protein kinase activity	2.23	1.87E-02	1.82E-01
GO:0010038~response to metal ion	2.13	1.88E-02	1.83E-01
GO:0003702~RNA polymerase II transcription factor activity	1.77	1.91E-02	3.09E-01
GO:0016019~peptidoglycan receptor activity	12.92	1.95E-02	3.09E-01
GO:0031402~sodium ion binding	2.21	1.99E-02	3.08E-01
GO:0008093~cytoskeletal adaptor activity	6.63	2.00E-02	3.05E-01
GO:0002251~organ or tissue specific immune response	12.70	2.02E-02	1.94E-01
GO:0032695~negative regulation of interleukin-12 production	12.70	2.02E-02	1.94E-01
GO:0002230~positive regulation of defense response to virus by host	12.70	2.02E-02	1.94E-01
GO:0010827~regulation of glucose transport	3.73	2.06E-02	1.97E-01
GO:0000060~protein import into nucleus, translocation	3.73	2.06E-02	1.97E-01
GO:0048820~hair follicle maturation	6.51	2.10E-02	2.00E-01
GO:0045995~regulation of embryonic development	6.51	2.10E-02	2.00E-01
GO:0022604~regulation of cell morphogenesis	2.10	2.10E-02	1.99E-01
GO:0009410~response to xenobiotic stimulus	4.60	2.13E-02	2.01E-01
GO:0010332~response to gamma radiation	4.60	2.13E-02	2.01E-01
GO:0008034~lipoprotein binding	3.69	2.16E-02	3.20E-01
GO:0042802~identical protein binding	1.41	2.23E-02	3.23E-01
GO:0005924~cell-substrate adherens junction	2.28	2.23E-02	3.52E-01
GO:0051606~detection of stimulus	2.15	2.36E-02	2.20E-01
GO:0006468~protein amino acid phosphorylation	1.40	2.36E-02	2.20E-01
GO:0005912~adherens junction	1.98	2.45E-02	3.64E-01
GO:0045936~negative regulation of phosphate metabolic process	3.09	2.45E-02	2.27E-01
GO:0010563~negative regulation of phosphorus metabolic process	3.09	2.45E-02	2.27E-01

GO:0030246~carbohydrate binding	1.58	2.46E-02	3.44E-01
GO:0016641~oxidoreductase activity, acting on the CH-NH2 group of donors, oxygen as a	6.15	2.46E-02	3.40E-01
GO:0022414~reproductive process	1.36	2.48E-02	2.28E-01
GO:0000267~cell fraction	1.30	2.51E-02	3.57E-01
GO:0070161~anchoring junction	1.92	2.53E-02	3.46E-01
GO:0006575~cellular amino acid derivative metabolic process	1.91	2.55E-02	2.33E-01
GO:0051716~cellular response to stimulus	1.34	2.56E-02	2.33E-01
GO:0050716~positive regulation of interleukin-1 secretion	6.05	2.58E-02	2.34E-01
GO:0045604~regulation of epidermal cell differentiation	6.05	2.58E-02	2.34E-01
GO:0031012~extracellular matrix	1.59	2.58E-02	3.40E-01
GO:0046425~regulation of JAK-STAT cascade	3.53	2.59E-02	2.34E-01
GO:0007205~activation of protein kinase C activity by G-protein coupled receptor protei	3.53	2.59E-02	2.34E-01
GO:0006865~amino acid transport	2.35	2.59E-02	2.34E-01
GO:0040007~growth	1.85	2.66E-02	2.39E-01
GO:0005520~insulin-like growth factor binding	4.31	2.67E-02	3.57E-01
GO:0034101~erythrocyte homeostasis	3.02	2.69E-02	2.40E-01
GO:0000003~reproduction	1.35	2.72E-02	2.43E-01
GO:0051128~regulation of cellular component organization	1.48	2.74E-02	2.43E-01
GO:0009314~response to radiation	1.80	2.75E-02	2.43E-01
GO:0048878~chemical homeostasis	1.45	2.76E-02	2.44E-01
GO:0007346~regulation of mitotic cell cycle	1.95	2.77E-02	2.44E-01
GO:0032768~regulation of monooxygenase activity	4.23	2.82E-02	2.47E-01
GO:0006801~superoxide metabolic process	4.23	2.82E-02	2.47E-01
GO:0009055~electron carrier activity	1.75	2.84E-02	3.71E-01
GO:0060589~nucleoside-triphosphatase regulator activity	1.51	2.87E-02	3.68E-01
GO:0044236~multicellular organismal metabolic process	3.43	2.88E-02	2.51E-01
GO:0019722~calcium-mediated signaling	3.43	2.88E-02	2.51E-01
GO:0005625~soluble fraction	1.61	2.88E-02	3.59E-01
GO:0030277~maintenance of gastrointestinal epithelium	10.58	2.94E-02	2.55E-01
GO:0051797~regulation of hair follicle development	10.58	2.94E-02	2.55E-01
GO:0032735~positive regulation of interleukin-12 production	10.58	2.94E-02	2.55E-01
GO:0032230~positive regulation of synaptic transmission, GABAergic	10.58	2.94E-02	2.55E-01
GO:0042634~regulation of hair cycle	10.58	2.94E-02	2.55E-01
GO:0010669~epithelial structure maintenance	10.58	2.94E-02	2.55E-01
GO:0002755~MyD88-dependent toll-like receptor signaling pathway	10.58	2.94E-02	2.55E-01
GO:0050901~leukocyte tethering or rolling	10.58	2.94E-02	2.55E-01
GO:0042402~biogenic amine catabolic process	5.64	3.11E-02	2.67E-01
GO:0050931~pigment cell differentiation	5.64	3.11E-02	2.67E-01
GO:0030804~positive regulation of cyclic nucleotide biosynthetic process	5.64	3.11E-02	2.67E-01
GO:0030801~positive regulation of cyclic nucleotide metabolic process	5.64	3.11E-02	2.67E-01
GO:0050704~regulation of interleukin-1 secretion	5.64	3.11E-02	2.67E-01
GO:0002687~positive regulation of leukocyte migration	5.64	3.11E-02	2.67E-01
GO:0030810~positive regulation of nucleotide biosynthetic process	5.64	3.11E-02	2.67E-01
GO:0045981~positive regulation of nucleotide metabolic process	5.64	3.11E-02	2.67E-01
GO:0014031~mesenchymal cell development	2.90	3.20E-02	2.73E-01
GO:0048762~mesenchymal cell differentiation	2.90	3.20E-02	2.73E-01
GO:0046427~positive regulation of JAK-STAT cascade	4.07	3.21E-02	2.73E-01
GO:0045766~positive regulation of angiogenesis	4.07	3.21E-02	2.73E-01
GO:0051353~positive regulation of oxidoreductase activity	4.07	3.21E-02	2.73E-01
GO:0015370~solute:sodium symporter activity	2.90	3.24E-02	3.99E-01
GO:0040008~regulation of growth	1.55	3.27E-02	2.76E-01
GO:0046982~protein heterodimerization activity	1.76	3.28E-02	3.99E-01
GO:0048754~branching morphogenesis of a tube	2.60	3.29E-02	2.77E-01
GO:0002253~activation of immune response	2.25	3.31E-02	2.78E-01
GO:0001932~regulation of protein amino acid phosphorylation	1.83	3.46E-02	2.88E-01
GO:0050878~regulation of body fluid levels	1.95	3.46E-02	2.88E-01
GO:0060485~mesenchyme development	2.85	3.48E-02	2.88E-01
GO:0060090~molecular adaptor activity	2.57	3.50E-02	4.14E-01

GO:0014075~response to amine stimulus	3.26	3.52E-02	2.90E-01
GO:0051781~positive regulation of cell division	3.26	3.52E-02	2.90E-01
GO:0007613~memory	3.26	3.52E-02	2.90E-01
GO:0005578~proteinaceous extracellular matrix	1.58	3.55E-02	4.10E-01
GO:0045598~regulation of fat cell differentiation	5.29	3.70E-02	3.02E-01
GO:0031093~platelet alpha granule lumen	3.22	3.71E-02	4.12E-01
GO:0030695~GTPase regulator activity	1.49	3.73E-02	4.29E-01
GO:0033157~regulation of intracellular protein transport	2.80	3.78E-02	3.07E-01
GO:0016564~transcription repressor activity	1.57	3.78E-02	4.28E-01
GO:0042803~protein homodimerization activity	1.55	3.82E-02	4.26E-01
GO:0005138~interleukin-6 receptor binding	9.23	3.86E-02	4.25E-01
GO:0016709~oxidoreductase activity, acting on paired donors, with incorporation or red	3.85	3.87E-02	4.21E-01
GO:0051341~regulation of oxidoreductase activity	3.17	3.88E-02	3.13E-01
GO:0010632~regulation of epithelial cell migration	9.07	3.99E-02	3.19E-01
GO:0045080~positive regulation of chemokine biosynthetic process	9.07	3.99E-02	3.19E-01
GO:0009253~peptidoglycan catabolic process	9.07	3.99E-02	3.19E-01
GO:0000270~peptidoglycan metabolic process	9.07	3.99E-02	3.19E-01
GO:0050665~hydrogen peroxide biosynthetic process	9.07	3.99E-02	3.19E-01
GO:0030949~positive regulation of vascular endothelial growth factor receptor signaling	9.07	3.99E-02	3.19E-01
GO:0051968~positive regulation of synaptic transmission, glutamatergic	9.07	3.99E-02	3.19E-01
GO:0043114~regulation of vascular permeability	9.07	3.99E-02	3.19E-01
GO:0032722~positive regulation of chemokine production	9.07	3.99E-02	3.19E-01
GO:0007159~leukocyte adhesion	3.78	4.09E-02	3.26E-01
GO:0002683~negative regulation of immune system process	2.29	4.17E-02	3.30E-01
GO:0002700~regulation of production of molecular mediator of immune response	3.10	4.25E-02	3.35E-01
GO:0032844~regulation of homeostatic process	2.04	4.29E-02	3.37E-01
GO:0045893~positive regulation of transcription, DNA-dependent	1.42	4.31E-02	3.37E-01
GO:0046330~positive regulation of JNK cascade	4.98	4.34E-02	3.38E-01
GO:0042107~cytokine metabolic process	4.98	4.34E-02	3.38E-01
GO:0070302~regulation of stress-activated protein kinase signaling pathway	2.45	4.35E-02	3.38E-01
GO:0030324~lung development	2.14	4.39E-02	3.40E-01
GO:0001503~ossification	2.02	4.51E-02	3.47E-01
GO:0004713~protein tyrosine kinase activity	1.82	4.52E-02	4.67E-01
GO:0008237~metallopeptidase activity	1.77	4.56E-02	4.65E-01
GO:0050921~positive regulation of chemotaxis	3.65	4.57E-02	3.50E-01
GO:0010648~negative regulation of cell communication	1.62	4.58E-02	3.50E-01
GO:0019222~regulation of metabolic process	1.12	4.59E-02	3.49E-01
GO:0001942~hair follicle development	3.02	4.64E-02	3.52E-01
GO:0022404~molting cycle process	3.02	4.64E-02	3.52E-01
GO:0022405~hair cycle process	3.02	4.64E-02	3.52E-01
GO:0007154~cell communication	1.30	4.68E-02	3.54E-01
GO:0031968~organelle outer membrane	2.11	4.70E-02	4.78E-01
GO:0051254~positive regulation of RNA metabolic process	1.41	4.75E-02	3.57E-01
GO:0000082~G1/S transition of mitotic cell cycle	2.65	4.75E-02	3.56E-01
GO:0051783~regulation of nuclear division	2.65	4.75E-02	3.56E-01
GO:0009612~response to mechanical stimulus	2.65	4.75E-02	3.56E-01
GO:0048771~tissue remodeling	2.65	4.75E-02	3.56E-01
GO:0007088~regulation of mitosis	2.65	4.75E-02	3.56E-01
GO:0031965~nuclear membrane	2.41	4.76E-02	4.70E-01
GO:0060205~cytoplasmic membrane-bounded vesicle lumen	3.00	4.82E-02	4.63E-01
GO:0050678~regulation of epithelial cell proliferation	2.38	4.95E-02	3.68E-01
GO:0019915~lipid storage	4.70	5.03E-02	3.72E-01
GO:0043154~negative regulation of caspase activity	4.70	5.03E-02	3.72E-01
GO:0042345~regulation of NF-kappaB import into nucleus	4.70	5.03E-02	3.72E-01
GO:0003712~transcription cofactor activity	1.48	5.05E-02	4.96E-01
GO:0034599~cellular response to oxidative stress	2.95	5.06E-02	3.73E-01
GO:0042633~hair cycle	2.95	5.06E-02	3.73E-01
GO:0042303~molting cycle	2.95	5.06E-02	3.73E-01

GO:0030218~erythrocyte differentiation	2.95	5.06E-02	3.73E-01
GO:0004871~signal transducer activity	1.16	5.06E-02	4.92E-01
GO:0060089~molecular transducer activity	1.16	5.06E-02	4.92E-01
GO:0032649~regulation of interferon-gamma production	3.53	5.09E-02	3.73E-01
GO:0032663~regulation of interleukin-2 production	3.53	5.09E-02	3.73E-01
GO:0030323~respiratory tube development	2.07	5.14E-02	3.76E-01
GO:0019932~second-messenger-mediated signaling	1.62	5.24E-02	3.81E-01
GO:0006357~regulation of transcription from RNA polymerase II promoter	1.31	5.26E-02	3.81E-01
GO:0007166~cell surface receptor linked signal transduction	1.17	5.29E-02	3.82E-01
GO:0016814~hydrolase activity, acting on carbon-nitrogen (but not peptide) bonds, in cy	3.47	5.34E-02	5.06E-01
GO:0080135~regulation of cellular response to stress	2.05	5.41E-02	3.88E-01
GO:0008134~transcription factor binding	1.39	5.43E-02	5.07E-01
GO:0048732~gland development	1.88	5.44E-02	3.89E-01
GO:0020037~heme binding	1.96	5.45E-02	5.03E-01
GO:0015171~amino acid transmembrane transporter activity	2.56	5.48E-02	5.00E-01
GO:0005178~integrin binding	2.56	5.48E-02	5.00E-01
GO:0003018~vascular process in circulatory system	2.55	5.48E-02	3.91E-01
GO:0046822~regulation of nucleocytoplasmic transport	2.55	5.48E-02	3.91E-01
GO:0007588~excretion	2.55	5.48E-02	3.91E-01
GO:0042306~regulation of protein import into nucleus	2.89	5.50E-02	3.91E-01
GO:0042509~regulation of tyrosine phosphorylation of STAT protein	3.41	5.63E-02	3.98E-01
GO:0050920~regulation of chemotaxis	3.41	5.63E-02	3.98E-01
GO:0031983~vesicle lumen	2.87	5.66E-02	5.08E-01
GO:0010628~positive regulation of gene expression	1.35	5.67E-02	3.99E-01
GO:0006725~cellular aromatic compound metabolic process	1.87	5.68E-02	3.99E-01
GO:0050804~regulation of synaptic transmission	1.87	5.68E-02	3.99E-01
GO:0019867~outer membrane	2.03	5.72E-02	5.00E-01
GO:0006693~prostaglandin metabolic process	4.46	5.77E-02	4.03E-01
GO:0006692~prostanoid metabolic process	4.46	5.77E-02	4.03E-01
GO:0042219~cellular amino acid derivative catabolic process	4.46	5.77E-02	4.03E-01
GO:0051966~regulation of synaptic transmission, glutamatergic	4.46	5.77E-02	4.03E-01
GO:0031644~regulation of neurological system process	1.80	5.82E-02	4.05E-01
GO:0009968~negative regulation of signal transduction	1.63	5.91E-02	4.09E-01
GO:0031323~regulation of cellular metabolic process	1.11	5.95E-02	4.10E-01
GO:0001763~morphogenesis of a branching structure	2.29	5.95E-02	4.09E-01
GO:0055066~di-, tri-valent inorganic cation homeostasis	1.59	5.95E-02	4.09E-01
GO:0005856~cytoskeleton	1.21	5.97E-02	5.04E-01
GO:0009308~amine metabolic process	1.43	6.00E-02	4.11E-01
GO:0043020~NADPH oxidase complex	7.32	6.01E-02	4.96E-01
GO:0005099~Ras GTPase activator activity	2.13	6.01E-02	5.29E-01
GO:0043434~response to peptide hormone stimulus	1.79	6.06E-02	4.13E-01
GO:0055065~metal ion homeostasis	1.65	6.14E-02	4.16E-01
GO:0045582~positive regulation of T cell differentiation	3.31	6.21E-02	4.19E-01
GO:0030168~platelet activation	3.31	6.21E-02	4.19E-01
GO:0001933~negative regulation of protein amino acid phosphorylation	3.31	6.21E-02	4.19E-01
GO:0009790~embryonic development	1.34	6.26E-02	4.21E-01
GO:0060249~anatomical structure homeostasis	2.00	6.27E-02	4.21E-01
GO:0007179~transforming growth factor beta receptor signaling pathway	2.47	6.27E-02	4.20E-01
GO:0030522~intracellular receptor-mediated signaling pathway	2.26	6.31E-02	4.21E-01
GO:0008361~regulation of cell size	1.64	6.35E-02	4.22E-01
GO:0052173~response to defenses of other organism during symbiotic interaction	7.05	6.42E-02	4.25E-01
GO:0010574~regulation of vascular endothelial growth factor production	7.05	6.42E-02	4.25E-01
GO:0030947~regulation of vascular endothelial growth factor receptor signaling pathwa	7.05	6.42E-02	4.25E-01
GO:0051023~regulation of immunoglobulin secretion	7.05	6.42E-02	4.25E-01
GO:0035162~embryonic hemopoiesis	7.05	6.42E-02	4.25E-01
GO:0046325~negative regulation of glucose import	7.05	6.42E-02	4.25E-01
GO:0010717~regulation of epithelial to mesenchymal transition	7.05	6.42E-02	4.25E-01
GO:0042640~anagen	7.05	6.42E-02	4.25E-01

GO:0043536~positive regulation of blood vessel endothelial cell migration	7.05	6.42E-02	4.25E-01
GO:0048246~macrophage chemotaxis	7.05	6.42E-02	4.25E-01
GO:0052200~response to host defenses	7.05	6.42E-02	4.25E-01
GO:0050691~regulation of defense response to virus by host	7.05	6.42E-02	4.25E-01
GO:0075136~response to host	7.05	6.42E-02	4.25E-01
GO:0002274~myeloid leukocyte activation	2.76	6.43E-02	4.25E-01
GO:0005737~cytoplasm	1.06	6.48E-02	5.13E-01
GO:0019898~extrinsic to membrane	1.38	6.54E-02	5.06E-01
GO:0060348~bone development	1.89	6.54E-02	4.30E-01
GO:0045104~intermediate filament cytoskeleton organization	4.23	6.56E-02	4.30E-01
GO:0051385~response to mineralocorticoid stimulus	4.23	6.56E-02	4.30E-01
GO:0032651~regulation of interleukin-1 beta production	4.23	6.56E-02	4.30E-01
GO:0040013~negative regulation of locomotion	2.43	6.69E-02	4.36E-01
GO:0002703~regulation of leukocyte mediated immunity	2.43	6.69E-02	4.36E-01
GO:0009636~response to toxin	2.43	6.69E-02	4.36E-01
GO:0010876~lipid localization	1.75	6.80E-02	4.40E-01
GO:0050871~positive regulation of B cell activation	3.21	6.81E-02	4.40E-01
GO:0046324~regulation of glucose import	3.21	6.81E-02	4.40E-01
GO:0030308~negative regulation of cell growth	2.07	6.86E-02	4.42E-01
GO:0009987~cellular process	1.03	6.88E-02	4.42E-01
GO:0060541~respiratory system development	1.96	6.89E-02	4.42E-01
GO:0006690~icosanoid metabolic process	2.70	6.93E-02	4.43E-01
GO:0051302~regulation of cell division	2.70	6.93E-02	4.43E-01
GO:0050731~positive regulation of peptidyl-tyrosine phosphorylation	2.70	6.93E-02	4.43E-01
GO:0006793~phosphorus metabolic process	1.24	6.94E-02	4.43E-01
GO:0006796~phosphate metabolic process	1.24	6.94E-02	4.43E-01
GO:0006519~cellular amino acid and derivative metabolic process	1.44	7.06E-02	4.48E-01
GO:0002250~adaptive immune response	2.20	7.06E-02	4.47E-01
GO:0002460~adaptive immune response based on somatic recombination of immune re	2.20	7.06E-02	4.47E-01
GO:0022602~ovulation cycle process	2.39	7.13E-02	4.49E-01
GO:0043408~regulation of MAPKKK cascade	1.94	7.21E-02	4.53E-01
GO:0032994~protein-lipid complex	3.14	7.28E-02	5.35E-01
GO:0034358~plasma lipoprotein particle	3.14	7.28E-02	5.35E-01
GO:0016773~phosphotransferase activity, alcohol group as acceptor	1.28	7.32E-02	5.98E-01
GO:0010466~negative regulation of peptidase activity	4.03	7.39E-02	4.60E-01
GO:0002673~regulation of acute inflammatory response	4.03	7.39E-02	4.60E-01
GO:0050801~ion homeostasis	1.40	7.41E-02	4.60E-01
GO:0009712~catechol metabolic process	3.11	7.44E-02	4.61E-01
GO:0034311~diol metabolic process	3.11	7.44E-02	4.61E-01
GO:0006584~catecholamine metabolic process	3.11	7.44E-02	4.61E-01
GO:0048520~positive regulation of behavior	3.11	7.44E-02	4.61E-01
GO:0007568~aging	1.92	7.55E-02	4.65E-01
GO:0044419~interspecies interaction between organisms	1.50	7.63E-02	4.68E-01
GO:0001501~skeletal system development	1.46	7.64E-02	4.68E-01
GO:0046906~tetrapyrrole binding	1.84	7.68E-02	6.11E-01
GO:0050829~defense response to Gram-negative bacterium	6.35	7.78E-02	4.73E-01
GO:0032891~negative regulation of organic acid transport	6.35	7.78E-02	4.73E-01
GO:0042088~T-helper 1 type immune response	6.35	7.78E-02	4.73E-01
GO:0046685~response to arsenic	6.35	7.78E-02	4.73E-01
GO:0006875~cellular metal ion homeostasis	1.62	8.05E-02	4.84E-01
GO:0051235~maintenance of location	2.31	8.05E-02	4.84E-01
GO:0048145~regulation of fibroblast proliferation	3.02	8.10E-02	4.85E-01
GO:0045621~positive regulation of lymphocyte differentiation	3.02	8.10E-02	4.85E-01
GO:0018958~phenol metabolic process	3.02	8.10E-02	4.85E-01
GO:0051345~positive regulation of hydrolase activity	1.66	8.13E-02	4.85E-01
GO:0008633~activation of pro-apoptotic gene products	3.85	8.27E-02	4.91E-01
GO:0007157~heterophilic cell adhesion	3.85	8.27E-02	4.91E-01
GO:0045670~regulation of osteoclast differentiation	3.85	8.27E-02	4.91E-01

GO:0046824~positive regulation of nucleocytoplasmic transport	3.85	8.27E-02	4.91E-01
GO:0048286~lung alveolus development	3.85	8.27E-02	4.91E-01
GO:0045103~intermediate filament-based process	3.85	8.27E-02	4.91E-01
GO:0045941~positive regulation of transcription	1.31	8.32E-02	4.92E-01
GO:0032270~positive regulation of cellular protein metabolic process	1.54	8.41E-02	4.96E-01
GO:0007507~heart development	1.57	8.44E-02	4.96E-01
GO:0007264~small GTPase mediated signal transduction	1.46	8.47E-02	4.96E-01
GO:0005829~cytosol	1.19	8.51E-02	5.84E-01
GO:0008233~peptidase activity	1.31	8.65E-02	6.52E-01
GO:0051056~regulation of small GTPase mediated signal transduction	1.51	8.66E-02	5.04E-01
GO:0005083~small GTPase regulator activity	1.49	8.68E-02	6.49E-01
GO:0045596~negative regulation of cell differentiation	1.57	8.70E-02	5.04E-01
GO:0006979~response to oxidative stress	1.68	8.76E-02	5.06E-01
GO:0046942~carboxylic acid transport	1.73	8.78E-02	5.06E-01
GO:0051969~regulation of transmission of nerve impulse	1.73	8.78E-02	5.06E-01
GO:0031331~positive regulation of cellular catabolic process	2.94	8.79E-02	5.06E-01
GO:0031348~negative regulation of defense response	2.94	8.79E-02	5.06E-01
GO:0002263~cell activation during immune response	2.94	8.79E-02	5.06E-01
GO:0007585~respiratory gaseous exchange	2.94	8.79E-02	5.06E-01
GO:0002366~leukocyte activation during immune response	2.94	8.79E-02	5.06E-01
GO:0048019~receptor antagonist activity	5.87	8.95E-02	6.56E-01
GO:0030547~receptor inhibitor activity	5.87	8.95E-02	6.56E-01
GO:0010564~regulation of cell cycle process	1.86	8.97E-02	5.12E-01
GO:0009123~nucleoside monophosphate metabolic process	2.24	9.04E-02	5.14E-01
GO:0046883~regulation of hormone secretion	2.24	9.04E-02	5.14E-01
GO:0031324~negative regulation of cellular metabolic process	1.26	9.04E-02	5.14E-01
GO:0016310~phosphorylation	1.24	9.06E-02	5.14E-01
GO:0019964~interferon-gamma binding	21.54	9.06E-02	6.56E-01
GO:0004906~interferon-gamma receptor activity	21.54	9.06E-02	6.56E-01
GO:0019962~type I interferon binding	21.54	9.06E-02	6.56E-01
GO:0015111~iodide transmembrane transporter activity	21.54	9.06E-02	6.56E-01
GO:0004909~interleukin-1, Type I, activating receptor activity	21.54	9.06E-02	6.56E-01
GO:0004905~type I interferon receptor activity	21.54	9.06E-02	6.56E-01
GO:0008297~single-stranded DNA specific exodeoxyribonuclease activity	21.54	9.06E-02	6.56E-01
GO:0015849~organic acid transport	1.72	9.10E-02	5.15E-01
GO:0033559~unsaturated fatty acid metabolic process	2.49	9.14E-02	5.15E-01
GO:0050864~regulation of B cell activation	2.49	9.14E-02	5.15E-01
GO:0045727~positive regulation of translation	3.68	9.20E-02	5.17E-01
GO:0043271~negative regulation of ion transport	3.68	9.20E-02	5.17E-01
GO:0042531~positive regulation of tyrosine phosphorylation of STAT protein	3.68	9.20E-02	5.17E-01
GO:0034116~positive regulation of heterotypic cell-cell adhesion	21.16	9.21E-02	5.17E-01
GO:0034142~toll-like receptor 4 signaling pathway	21.16	9.21E-02	5.17E-01
GO:0034122~negative regulation of toll-like receptor signaling pathway	21.16	9.21E-02	5.17E-01
GO:0070163~regulation of adiponectin secretion	21.16	9.21E-02	5.17E-01
GO:0070427~nucleotide-binding oligomerization domain containing 1 signaling pathway	21.16	9.21E-02	5.17E-01
GO:0002439~chronic inflammatory response to antigenic stimulus	21.16	9.21E-02	5.17E-01
GO:0030850~prostate gland development	5.77	9.22E-02	5.16E-01
GO:0022409~positive regulation of cell-cell adhesion	5.77	9.22E-02	5.16E-01
GO:0045649~regulation of macrophage differentiation	5.77	9.22E-02	5.16E-01
GO:0045616~regulation of keratinocyte differentiation	5.77	9.22E-02	5.16E-01
GO:0080010~regulation of oxygen and reactive oxygen species metabolic process	5.77	9.22E-02	5.16E-01
GO:0009892~negative regulation of metabolic process	1.25	9.42E-02	5.23E-01
GO:0031214~biomineral formation	2.86	9.51E-02	5.26E-01
GO:0045792~negative regulation of cell size	1.92	9.55E-02	5.27E-01
GO:0042698~ovulation cycle	2.21	9.55E-02	5.26E-01
GO:0000165~MAPKKK cascade	1.61	9.57E-02	5.26E-01
GO:0019899~enzyme binding	1.32	9.60E-02	6.74E-01
GO:0019882~antigen processing and presentation	2.04	9.64E-02	5.28E-01

GO:0051291~protein heterooligomerization	2.44	9.74E-02	5.31E-01
GO:0042169~SH2 domain binding	3.59	9.76E-02	6.76E-01
