# Supplementary material for: Plasticity of Airway Epithelial Cell Transcriptome in Response to Flagellin
Source: PLoS One. 2015 Feb 10;10(2):e0115486. doi: 10.1371/journal.pone.0115486 (PMC4323341; doi:10.1371/journal.pone.0115486)
Supplement: S5 Table — (PDF) [file pone.0115486.s006.pdf]

Supplementary Table 5. Complete results from functional enrichment analysis of differentially expressed genes following exposure to flagellin in ALI AEC cultures as identified by exon microarrays.

Gene Ontology Annotation	Fold Enrichment	P-value	Adjusted P-value
GO:0008009~chemokine activity	246.90	2.70E-05	4.05E-04
GO:0040011~locomotion	32.75	2.83E-05	6.79E-03
GO:0042379~chemokine receptor binding	231.78	3.07E-05	2.30E-04
GO:0050900~leukocyte migration	185.74	4.79E-05	5.76E-03
GO:0005615~extracellular space	23.22	7.95E-05	1.67E-03
GO:0006955~immune response	20.46	1.16E-04	9.30E-03
GO:0001664~G-protein-coupled receptor binding	102.32	1.59E-04	7.95E-04
GO:0005102~receptor binding	17.09	2.00E-04	7.48E-04
GO:0044421~extracellular region part	16.57	2.19E-04	2.30E-03
GO:0002376~immune system process	14.14	3.52E-04	2.10E-02
GO:0006935~chemotaxis	66.17	3.80E-04	1.82E-02
GO:0042330~taxis	66.17	3.80E-04	1.82E-02
GO:0005125~cytokine activity	58.24	4.91E-04	1.47E-03
GO:0007626~locomotory behavior	38.64	1.11E-03	4.37E-02
GO:0016477~cell migration	38.36	1.13E-03	3.81E-02
GO:0048870~cell motility	34.49	1.39E-03	4.12E-02
GO:0051674~localization of cell	34.49	1.39E-03	4.12E-02
GO:0006954~inflammatory response	32.58	1.56E-03	4.10E-02
GO:0005576~extracellular region	7.91	2.01E-03	1.40E-02
GO:0045069~regulation of viral genome replication	504.14	2.97E-03	6.92E-02
GO:0048583~regulation of response to stimulus	22.77	3.18E-03	6.74E-02
GO:0007610~behavior	22.57	3.23E-03	6.29E-02
GO:0006928~cell motion	22.29	3.31E-03	5.97E-02
GO:0009611~response to wounding	19.98	4.12E-03	6.85E-02
GO:0050792~regulation of viral reproduction	336.10	4.46E-03	6.92E-02
GO:0006952~defense response	17.21	5.52E-03	8.00E-02
GO:0051704~multi-organism process	15.55	6.75E-03	9.15E-02
GO:0030595~leukocyte chemotaxis	190.76	7.84E-03	1.00E-01
GO:0060326~cell chemotaxis	180.97	8.27E-03	9.99E-02
GO:0009605~response to external stimulus	11.58	1.20E-02	1.36E-01
GO:0032103~positive regulation of response to external stimulus	110.28	1.35E-02	1.45E-01
GO:0050896~response to stimulus	4.03	1.53E-02	1.55E-01
GO:0007186~G-protein coupled receptor protein signaling pathway	9.43	1.80E-02	1.73E-01
GO:0040017~positive regulation of locomotion	72.02	2.07E-02	1.89E-01
GO:0042221~response to chemical stimulus	8.26	2.32E-02	2.02E-01
GO:0030155~regulation of cell adhesion	51.52	2.88E-02	2.38E-01
GO:0032101~regulation of response to external stimulus	44.39	3.34E-02	2.62E-01
GO:0006950~response to stress	6.28	3.93E-02	2.92E-01
GO:0040012~regulation of locomotion	36.76	4.03E-02	2.89E-01
GO:0009617~response to bacterium	36.57	4.05E-02	2.82E-01
GO:0007166~cell surface receptor linked signal transduction	5.70	4.73E-02	3.14E-01
GO:0048584~positive regulation of response to stimulus	29.91	4.93E-02	3.17E-01
GO:0045321~leukocyte activation	29.17	5.06E-02	3.15E-01
GO:0048518~positive regulation of biological process	5.21	5.62E-02	3.37E-01
GO:0001775~cell activation	24.59	5.98E-02	3.46E-01
GO:0051707~response to other organism	24.34	6.04E-02	3.41E-01
GO:0009607~response to biotic stimulus	18.38	7.94E-02	4.17E-01
GO:0002682~regulation of immune system process	18.33	7.96E-02	4.09E-01
