# Supplementary material for: Plasticity of Airway Epithelial Cell Transcriptome in Response to Flagellin
Source: PLoS One. 2015 Feb 10;10(2):e0115486. doi: 10.1371/journal.pone.0115486 (PMC4323341; doi:10.1371/journal.pone.0115486)
Supplement: S6 Table — (PDF) [file pone.0115486.s007.pdf]

Supplementary Table 6. Complete results from functional enrichment analysis of differentially expressed genes following exposure to flagellin in ALI AEC cultures as identified by RNAseq.

**Gene Ontology Annotation**	**Fold Enrichment**	**P-value**	**Adjusted P-value**
GO:0005125~cytokine activity	18.79	2.86E-14	5.41E-12
GO:0005615~extracellular space	7.51	3.07E-13	4.14E-11
GO:0044421~extracellular region part	5.85	2.89E-12	1.95E-10
GO:0005576~extracellular region	3.38	2.35E-09	1.06E-07
GO:0008009~chemokine activity	37.17	2.74E-08	2.59E-06
GO:0042379~chemokine receptor binding	34.89	4.06E-08	2.56E-06
GO:0005102~receptor binding	4.96	4.43E-08	2.09E-06
GO:0006955~immune response	5.35	4.85E-08	5.21E-05
GO:0009611~response to wounding	6.15	7.92E-08	4.25E-05
GO:0006954~inflammatory response	8.02	2.04E-07	7.29E-05
GO:0002376~immune system process	4.13	2.68E-07	7.19E-05
GO:0008544~epidermis development	10.62	1.70E-06	3.65E-04
GO:0009605~response to external stimulus	4.04	2.15E-06	3.84E-04
GO:0006952~defense response	4.94	3.04E-06	4.66E-04
GO:0007398~ectoderm development	9.82	3.05E-06	4.10E-04
GO:0001664~G-protein-coupled receptor binding	15.40	5.42E-06	2.05E-04
GO:0050900~leukocyte migration	22.86	5.71E-06	6.81E-04
GO:0048513~organ development	2.75	1.48E-05	1.58E-03
GO:0040011~locomotion	5.54	2.20E-05	2.14E-03
GO:0031424~keratinization	25.25	4.15E-05	3.71E-03
GO:0006935~chemotaxis	9.50	8.41E-05	6.92E-03
GO:0042330~taxis	9.50	8.41E-05	6.92E-03
GO:0006950~response to stress	2.58	1.13E-04	8.61E-03
GO:0048731~system development	2.24	1.31E-04	9.31E-03
GO:0042221~response to chemical stimulus	2.88	1.42E-04	9.48E-03
GO:0048856~anatomical structure development	2.15	1.60E-04	1.01E-02
GO:0030216~keratinocyte differentiation	16.45	2.24E-04	1.33E-02
GO:0016477~cell migration	6.29	2.43E-04	1.36E-02
GO:0050896~response to stimulus	1.86	2.76E-04	1.47E-02
GO:0001775~cell activation	6.05	3.08E-04	1.56E-02
GO:0009913~epidermal cell differentiation	15.08	3.14E-04	1.52E-02
GO:0048870~cell motility	5.66	4.62E-04	2.13E-02
GO:0051674~localization of cell	5.66	4.62E-04	2.13E-02
GO:0002573~myeloid leukocyte differentiation	25.55	4.81E-04	2.13E-02
GO:0030595~leukocyte chemotaxis	23.48	6.19E-04	2.62E-02
GO:0060326~cell chemotaxis	22.27	7.23E-04	2.94E-02
GO:0032502~developmental process	1.86	7.48E-04	2.93E-02
GO:0009888~tissue development	3.59	7.61E-04	2.88E-02
GO:0001568~blood vessel development	6.20	8.31E-04	3.03E-02
GO:0002761~regulation of myeloid leukocyte differentiation	21.19	8.38E-04	2.96E-02
GO:0001944~vasculature development	6.06	9.42E-04	3.21E-02
GO:0007275~multicellular organismal development	1.90	1.10E-03	3.63E-02
GO:0048583~regulation of response to stimulus	4.20	1.14E-03	3.66E-02
GO:0007626~locomotory behavior	5.55	1.48E-03	4.57E-02
GO:0009607~response to biotic stimulus	4.52	1.71E-03	5.11E-02
GO:0045069~regulation of viral genome replication	46.54	1.78E-03	5.17E-02
GO:0005515~protein binding	1.35	2.63E-03	7.96E-02
GO:0002521~leukocyte differentiation	8.29	2.93E-03	8.16E-02
GO:0002763~positive regulation of myeloid leukocyte differentiation	36.19	2.95E-03	8.02E-02

GO:0032103~positive regulation of response to external stimulus	13.57	3.04E-03	8.05E-02
GO:0030855~epithelial cell differentiation	7.93	3.44E-03	8.83E-02
GO:0030155~regulation of cell adhesion	7.93	3.44E-03	8.83E-02
GO:0019221~cytokine-mediated signaling pathway	12.41	3.92E-03	9.78E-02
GO:0045637~regulation of myeloid cell differentiation	12.41	3.92E-03	9.78E-02
GO:0008083~growth factor activity	7.59	4.01E-03	1.03E-01
GO:0050792~regulation of viral reproduction	31.02	4.02E-03	9.79E-02
GO:0007155~cell adhesion	3.10	4.11E-03	9.78E-02
GO:0022610~biological adhesion	3.10	4.15E-03	9.65E-02
GO:0048584~positive regulation of response to stimulus	5.52	4.28E-03	9.74E-02
GO:0001525~angiogenesis	7.34	4.53E-03	1.01E-01
GO:0045321~leukocyte activation	5.38	4.76E-03	1.03E-01
GO:0042531~positive regulation of tyrosine phosphorylation of STAT prote	28.33	4.81E-03	1.02E-01
GO:0050727~regulation of inflammatory response	11.43	4.94E-03	1.03E-01
GO:0010033~response to organic substance	3.01	4.99E-03	1.02E-01
GO:0010627~regulation of protein kinase cascade	5.23	5.37E-03	1.07E-01
GO:0006928~cell motion	3.66	5.55E-03	1.09E-01
GO:0030154~cell differentiation	2.12	5.59E-03	1.07E-01
GO:0032101~regulation of response to external stimulus	6.83	5.83E-03	1.10E-01
GO:0046427~positive regulation of JAK-STAT cascade	25.06	6.13E-03	1.13E-01
GO:0010740~positive regulation of protein kinase cascade	6.50	6.92E-03	1.25E-01
GO:0007166~cell surface receptor linked signal transduction	1.99	7.45E-03	1.31E-01
GO:0001934~positive regulation of protein amino acid phosphorylation	9.76	7.65E-03	1.33E-01
GO:0002682~regulation of immune system process	3.95	7.84E-03	1.33E-01
GO:0032501~multicellular organismal process	1.52	7.96E-03	1.33E-01
GO:0050729~positive regulation of inflammatory response	21.72	8.11E-03	1.34E-01
GO:0048869~cellular developmental process	2.04	8.15E-03	1.32E-01
GO:0051094~positive regulation of developmental process	4.69	8.46E-03	1.35E-01
GO:0030099~myeloid cell differentiation	9.34	8.63E-03	1.35E-01
GO:0042509~regulation of tyrosine phosphorylation of STAT protein	21.02	8.65E-03	1.34E-01
GO:0042127~regulation of cell proliferation	2.76	8.71E-03	1.33E-01
GO:0019838~growth factor binding	9.30	8.71E-03	1.87E-01
GO:0005581~collagen	20.05	9.51E-03	2.76E-01
GO:0042327~positive regulation of phosphorylation	8.96	9.68E-03	1.44E-01
GO:0045639~positive regulation of myeloid cell differentiation	19.74	9.76E-03	1.44E-01
GO:0045937~positive regulation of phosphate metabolic process	8.69	1.05E-02	1.52E-01
GO:0010562~positive regulation of phosphorus metabolic process	8.69	1.05E-02	1.52E-01
GO:0009967~positive regulation of signal transduction	4.42	1.08E-02	1.53E-01
GO:0051090~regulation of transcription factor activity	8.43	1.14E-02	1.59E-01
GO:0051704~multi-organism process	2.87	1.15E-02	1.58E-01
GO:0046425~regulation of JAK-STAT cascade	18.10	1.15E-02	1.57E-01
GO:0051789~response to protein stimulus	8.12	1.26E-02	1.68E-01
GO:0009615~response to virus	7.97	1.33E-02	1.74E-01
GO:0006916~anti-apoptosis	5.27	1.42E-02	1.83E-01
GO:0048514~blood vessel morphogenesis	5.15	1.53E-02	1.94E-01
GO:0010647~positive regulation of cell communication	3.96	1.66E-02	2.06E-01
GO:0048518~positive regulation of biological process	1.82	1.72E-02	2.10E-01
GO:0051101~regulation of DNA binding	7.18	1.75E-02	2.11E-01
GO:0042325~regulation of phosphorylation	3.26	1.87E-02	2.21E-01
GO:0050731~positive regulation of peptidyl-tyrosine phosphorylation	13.86	1.92E-02	2.24E-01
GO:0050776~regulation of immune response	4.80	1.92E-02	2.22E-01
GO:0007610~behavior	3.24	1.92E-02	2.20E-01
GO:0007167~enzyme linked receptor protein signaling pathway	3.81	1.93E-02	2.18E-01

GO:0060429~epithelium development	4.78	1.95E-02	2.18E-01
GO:0045597~positive regulation of cell differentiation	4.74	2.01E-02	2.22E-01
GO:0030097~hemopoiesis	4.60	2.22E-02	2.39E-01
GO:0019220~regulation of phosphate metabolic process	3.13	2.23E-02	2.38E-01
GO:0051174~regulation of phosphorus metabolic process	3.13	2.23E-02	2.38E-01
GO:0030851~granulocyte differentiation	86.87	2.25E-02	2.38E-01
GO:0005178~integrin binding	12.42	2.36E-02	3.94E-01
GO:0045071~negative regulation of viral genome replication	72.39	2.69E-02	2.75E-01
GO:0002544~chronic inflammatory response	72.39	2.69E-02	2.75E-01
GO:0031347~regulation of defense response	6.07	2.71E-02	2.74E-01
GO:0042981~regulation of apoptosis	2.43	2.82E-02	2.81E-01
GO:0043067~regulation of programmed cell death	2.41	2.97E-02	2.91E-01
GO:0048519~negative regulation of biological process	1.80	3.02E-02	2.93E-01
GO:0048534~hemopoietic or lymphoid organ development	4.18	3.02E-02	2.90E-01
GO:0010941~regulation of cell death	2.40	3.02E-02	2.88E-01
GO:0007179~transforming growth factor beta receptor signaling pathway	10.86	3.03E-02	2.86E-01
GO:0051716~cellular response to stimulus	2.38	3.12E-02	2.91E-01
GO:0048525~negative regulation of viral reproduction	62.05	3.13E-02	2.89E-01
GO:0051098~regulation of binding	5.68	3.22E-02	2.94E-01
GO:0048522~positive regulation of cellular process	1.76	3.48E-02	3.12E-01
GO:0080134~regulation of response to stress	3.96	3.56E-02	3.15E-01
GO:0002520~immune system development	3.93	3.64E-02	3.18E-01
GO:0050730~regulation of peptidyl-tyrosine phosphorylation	9.58	3.81E-02	3.28E-01
GO:0008284~positive regulation of cell proliferation	3.15	3.94E-02	3.34E-01
GO:0045684~positive regulation of epidermis development	48.26	4.01E-02	3.37E-01
GO:0006986~response to unfolded protein	9.18	4.12E-02	3.42E-01
GO:0009653~anatomical structure morphogenesis	2.00	4.20E-02	3.45E-01
GO:0051707~response to other organism	3.74	4.24E-02	3.45E-01
GO:0031349~positive regulation of defense response	8.92	4.33E-02	3.48E-01
GO:0048407~platelet-derived growth factor binding	44.41	4.34E-02	5.68E-01
GO:0001932~regulation of protein amino acid phosphorylation	5.02	4.38E-02	3.49E-01
GO:0045123~cellular extravasation	43.43	4.44E-02	3.51E-01
GO:0005578~proteinaceous extracellular matrix	3.66	4.59E-02	7.18E-01
GO:0042517~positive regulation of tyrosine phosphorylation of Stat3 prote	39.49	4.88E-02	3.76E-01
GO:0022409~positive regulation of cell-cell adhesion	39.49	4.88E-02	3.76E-01
GO:0005583~fibrillar collagen	38.99	4.94E-02	6.80E-01
GO:0031401~positive regulation of protein modification process	4.65	5.30E-02	3.98E-01
GO:0045885~positive regulation of survival gene product expression	36.19	5.31E-02	3.97E-01
GO:0050789~regulation of biological process	1.22	5.73E-02	4.18E-01
GO:0031012~extracellular matrix	3.39	5.75E-02	6.81E-01
GO:0009986~cell surface	3.36	5.90E-02	6.41E-01
GO:0019028~viral capsid	29.24	6.53E-02	6.37E-01
GO:0048523~negative regulation of cellular process	1.70	6.76E-02	4.71E-01
GO:0019012~virion	27.52	6.93E-02	6.21E-01
GO:0044423~virion part	27.52	6.93E-02	6.21E-01
GO:0045595~regulation of cell differentiation	2.65	7.19E-02	4.90E-01
GO:0031589~cell-substrate adhesion	6.65	7.29E-02	4.92E-01
GO:0042516~regulation of tyrosine phosphorylation of Stat3 protein	25.55	7.44E-02	4.96E-01
GO:0042044~fluid transport	25.55	7.44E-02	4.96E-01
GO:0048646~anatomical structure formation involved in morphogenesis	3.08	7.54E-02	4.98E-01
GO:0043066~negative regulation of apoptosis	3.07	7.66E-02	5.01E-01
GO:0002697~regulation of immune effector process	6.45	7.68E-02	4.99E-01
GO:0030593~neutrophil chemotaxis	24.13	7.86E-02	5.05E-01

GO:0019915~lipid storage	24.13	7.86E-02	5.05E-01
GO:0007178~transmembrane receptor protein serine/threonine kinase sig	6.33	7.94E-02	5.06E-01
GO:0043069~negative regulation of programmed cell death	3.02	7.97E-02	5.05E-01
GO:0060548~negative regulation of cell death	3.02	8.03E-02	5.05E-01
GO:0045860~positive regulation of protein kinase activity	3.90	8.04E-02	5.03E-01
GO:0065007~biological regulation	1.19	8.05E-02	5.00E-01
GO:0030198~extracellular matrix organization	6.26	8.08E-02	4.99E-01
GO:0008285~negative regulation of cell proliferation	3.01	8.10E-02	4.97E-01
GO:0050793~regulation of developmental process	2.26	8.42E-02	5.08E-01
GO:0043122~regulation of I-kappaB kinase/NF-kappaB cascade	6.09	8.48E-02	5.08E-01
GO:0044420~extracellular matrix part	6.00	8.71E-02	6.73E-01
GO:0033674~positive regulation of kinase activity	3.76	8.73E-02	5.16E-01
GO:0001533~cornified envelope	21.27	8.87E-02	6.48E-01
GO:0032270~positive regulation of cellular protein metabolic process	3.73	8.90E-02	5.21E-01
GO:0030968~endoplasmic reticulum unfolded protein response	20.68	9.11E-02	5.27E-01
GO:0045884~regulation of survival gene product expression	20.68	9.11E-02	5.27E-01
GO:0045429~positive regulation of nitric oxide biosynthetic process	20.68	9.11E-02	5.27E-01
GO:0034620~cellular response to unfolded protein	20.68	9.11E-02	5.27E-01
GO:0019216~regulation of lipid metabolic process	5.82	9.16E-02	5.27E-01
GO:0045682~regulation of epidermis development	19.74	9.52E-02	5.38E-01
GO:0007157~heterophilic cell adhesion	19.74	9.52E-02	5.38E-01
GO:0022407~regulation of cell-cell adhesion	19.74	9.52E-02	5.38E-01
GO:0045670~regulation of osteoclast differentiation	19.74	9.52E-02	5.38E-01
GO:0051347~positive regulation of transferase activity	3.62	9.52E-02	5.36E-01
GO:0051247~positive regulation of protein metabolic process	3.57	9.79E-02	5.44E-01
