# Supplementary material for: Plasticity of Airway Epithelial Cell Transcriptome in Response to Flagellin
Source: PLoS One. 2015 Feb 10;10(2):e0115486. doi: 10.1371/journal.pone.0115486 (PMC4323341; doi:10.1371/journal.pone.0115486)
Supplement: S1 Text — (PDF) [file pone.0115486.s008.pdf]

DETAILED MATERIALS AND METHODS

Ethics statement

The Institutional Review Board at Fred Hutchinson Cancer Research Center determined that since the tissue source was anonymous, it was not human research (NHR) and therefore waived the need for ethical review and informed consent. This policy was in accordance with Office for Human Research Protections guidelines (http://www.hhs.gov/ohrp/policy/cdebiol.html).

Human tracheal epithelial cell cultures and stimulation with flagellin

Human tracheal tissue was obtained from an anonymized lung transplant donor at University of Washington Medical Center and subjected to enzymatic digestion as described by Fulcher *et al.* [1] to isolate individual tracheal epithelial cells. Monolayer cells were grown on human placental collagen coated tissue culture plates with media changes every other day. Cells were grown until 90% confluent and then subcultured or harvested for cryopreservation. Monolayer cells were passaged no more than 3 times. To create ALI cultures, AECs were plated at 2.5×10^5 cells per cm^2 directly onto collagen-coated semipermeable supports that were 12 mm diameter and 0.4 µm pore size (Corning Transwell, Cambridge, MA). After incubation at 37ºC in a 5% CO_2 incubator, the apical media was removed to create an air-liquid interface (ALI) and cells were continuously cultured in Grays' Media [2] with media changes every two days. Approximately 4 weeks after plating, cells became well differentiated, containing both ciliated and mucous producing goblet cells with a transepithelial resistance > 500 $\Omega \cdot cm^2$.

When monolayer cultures reached ~ 90% confluency or the ALI cultures reached transepithelial electrical resistance > 500 $\Omega \cdot cm^2$, the AECs were stimulated with 1 µg/ml ultrapure flagellin (endotoxin contamination < 0.05 EU/µg) isolated from *Pseudomonas aeruginosa* (InvivoGen, San Diego, CA) for 4 hours at 37°C. The flagellin concentration of 1 µg/ml was based on previous reports [3,4], and our dose response experiments (0.1, 1, and 10 µg/ml) on ALI and monolayer cultures using an ELISA readout of IL8, a known flagellin-induced inflammatory cytokine, and CCL20, a differentially up-regulated product identified from the current study (Supplementary Figure 1). Conditioned media were collected for ELISA, and the epithelial cells were lysed in TRIzol and frozen at –80°C for RNA isolation. For all ELISA and RNA analyses, conditioned media and RNA were derived from one donor. The same RNA samples were used for PCR, microarrays and RNA-seq. All RNA-seq experiments were performed in duplicate, whereas the microarray experiments were either in triplicate (ALI cultures with and without flagellin exposure) or duplicate (monolayer cultures with and without flagellin exposure).

RNA isolation, qPCR and ELISA

Total RNA was isolated by TRIzol reagent (Invitrogen), quantified by NanoDrop 2000 (Thermo Scientific, Wilmington, DE), assessed for quality by Bioanalyzer (Agilent Technologies, Fort Worth, TX), and transcribed to cDNA by Superscriptase II (Invitrogen). Real time PCR was performed using TaqMan Gene Expression Master Mix, TaqMan primer probe sets (CCL2: Hs00234140_m1, CCL20: Hs01011368_m1, ICAM1: 00164932_m1, NOS2: Hs01075529_m1, DEFB4: Hs00175474_m1, 18s rRNA:

4333760F) in an ABI 7900 HT real time PCR system, and the cycle threshold (C_T) values were obtained by Sequence Detection System 2.3 (all from Applied Biosystems, Grand Island, NY). The 18S component of rRNA was used as the endogenous control and expression levels of the tested genes were normalized to its expression using the delta-delta approach [5]. Protein levels of CCL20 and IL8 in monolayer and ALI culture medium at baseline and following stimulation with flagellin were measured using ELISA kit (DuoSet DY360 and DY208 respectively from R&D System, Minneapolis, MN). The *P*-values for CCL20 and IL8 protein expression differences across samples was calculated using one-way ANOVA (GraphPad Software, La Jolla, CA).

Microarray

Total RNA (100 ng) from stimulated (flagellin 1 μg/ml) and unstimulated tracheal epithelial cells in either monolayer or ALI cultures was used to generate first strand cDNA after two cycles of *in vitro* transcription using Ambion's WT Expression Kit (Ambion, Grand Island, NY), and then fragmented and terminal-labeled by GeneChip WT Terminal Labeling Kit (Affymetrix, Santa Clara, CA). Samples were hybridized to Human Gene ST 1.0 microarrays (Affymetrix) at Fred Hutchinson Cancer Research Center's Genomics Resource Facility following the manufacturer's protocols. After hybridization and scanning, image acquisition was performed using the GeneChip Operating System (GCOS). Probe intensities were normalized using the RMA procedure as implemented in the aroma.affymetrix package for R (http://www.aroma-project.org/). Differential gene expression was determined using a Bayesian implementation of the parametric *t*-test applied to log-transformed probe intensities and optimized for low replicate numbers [6]. Correction for multiple testing was performed using Benjamini-Hochberg's method [7], with an adjusted *P*-value ≤ 0.01 designated as significant.

RNA-seq

Total RNA (1 μg) from stimulated (flagellin 1 μg/ml) and unstimulated tracheal epithelial cells in either monolayer or ALI cultures was used for library generation using Illumina's TruSeq RNA protocol (Illumina, San Diego, CA) at Fred Hutchinson Cancer Research Center's Genomics Resource. Next, image analysis and base calling were performed with Illumina's RTA (v1.12) software, followed by demultiplexing and FASTQ format file generation with Illumina's CASAVA (v1.8) software. After removing reads that did not pass Illumina's base call quality filter, alignment to UCSC's hg19 genome build was performed using TopHat (v1.3.1) [8]. The average number of alignments per run was approximately 20.5 million. Gene counts were generated using htseq-count (v0.5.1, http://www-huber.embl.de/users/anders/HTSeq/doc/count.html). For gene expression analysis, we initially removed all genes that had less than 1 count/million in at least half the samples. Differential gene expression analysis was performed using edgeR (v2.2.5, http://www.bioconductor.org/packages/2.8/bioc/html/edgeR.html) [9]. EdgeR is particularly suitable for analysis of experiments with low replicate numbers. Correction for multiple testing was implemented using Benjamini-Hochberg's method [7], with an adjusted *P*-value ≤ 0.01 designated as significant.

Correspondence and enrichment analyses

Multidimensional scaling using correspondence analysis was applied to compare variability in global gene expression across all samples as assessed by microarrays and RNA-seq [10,11]. This analysis was based on unique genes identified commonly between the microarray platform and RNA-seq.

Functional enrichment analysis of differentially expressed AEC genes in (i) unstimulated monolayer vs. unstimulated ALI, (ii) flagellin-exposed monolayer cultures, and (iii) flagellin-exposed ALI cultures, was performed using the DAVID online software tool (http://david.abcc.ncifcrf.gov/) and based on Gene Ontology annotations [12]. Since the number of differentially expressed genes in condition (i) was very large, we limited the enrichment analysis to the top 1000 most significant genes. Enrichment *P*-values were determined using a modified version of Fisher's exact test and corrected for multiple testing using Benjamini-Hochberg's method [7].

Data availability

Detailed microarray and RNA-seq information including access to all raw data, meeting Minimum Information About a Microarray Experiment (MIAME) requirements, has been deposited at Gene Expression Omnibus (http://www.ncbi.nlm.nih.gov/geo, GSE55460).

REFERENCES

1. Fulcher ML, Gabriel S, Burns KA, Yankaskas JR, Randell SH (2005) Well-differentiated human airway epithelial cell cultures. Methods in Molecular Medicine 107: 183-206.
2. Gray TE, Guzman K, Davis CW, Abdullah LH, Nettesheim P (1996) Mucociliary differentiation of serially passaged normal human tracheobronchial epithelial cells. American Journal of Respiratory Cell & Molecular Biology 14: 104-112.
3. Lopez-Boado YS, Wilson CL, Parks WC (2001) Regulation of matrilysin expression in airway epithelial cells by Pseudomonas aeruginosa flagellin. J Biol Chem 276: 41417-41423.
4. Tseng J, Do J, Widdicombe JH, Machen TE (2006) Innate immune responses of human tracheal epithelium to Pseudomonas aeruginosa flagellin, TNF-alpha, and IL-1beta. Am J Physiol Cell Physiol 290: C678-690.
5. Livak KJ, Schmittgen TD (2001) Analysis of relative gene expression data using real-time quantitative PCR and the 2(-Delta Delta C(T)) Method. Methods 25: 402-408.
6. Kayala MA, Baldi P (2012) Cyber-T web server: differential analysis of high-throughput data. Nucleic Acids Res 40: W553-559.
7. Benjamini Y, Hochberg Y (1995) Controlling the false discovery rate: a practical and powerful approach to multiple testing. Journal of the Royal Statistical Society Series B Methodological 57: 289-300
8. Trapnell C, Pachter L, Salzberg SL (2009) TopHat: discovering splice junctions with RNA-Seq. Bioinformatics 25: 1105-1111.
9. Robinson MD, McCarthy DJ, Smyth GK (2010) edgeR: a Bioconductor package for differential expression analysis of digital gene expression data. Bioinformatics 26: 139-140.
10. Saeed AI, Sharov V, White J, Li J, Liang W, et al. (2003) TM4: a free, open-source system for microarray data management and analysis. Biotechniques 34: 374-378.
11. Fellenberg K, Hauser NC, Brors B, Neutzner A, Hoheisel JD, et al. (2001) Correspondence analysis applied to microarray data. Proc Natl Acad Sci U S A 98: 10781-10786.
12. Huang da W, Sherman BT, Lempicki RA (2009) Systematic and integrative analysis of large gene lists using DAVID bioinformatics resources. Nat Protoc 4: 44-57.
